# The Synthesis, Crystal Structure, Modification, and Cytotoxic Activity of α-Hydroxy-Alkylphosphonates

**DOI:** 10.3390/molecules30020428

**Published:** 2025-01-20

**Authors:** Zsuzsanna Szalai, Anna Sára Kis, Angéla Takács, László Kőhidai, Konstantin Karaghiosoff, Mátyás Czugler, László Drahos, György Keglevich

**Affiliations:** 1 Department of Organic Chemistry and Technology, Faculty of Chemical Technology and Biotechnology, Budapest University of Technology and Economics, Műegyetem rkp. 3., 1111 Budapest, Hungary; szalai.zsuzsanna@edu.bme.hu (Z.S.); kisannasari@gmail.com (A.S.K.); czuglrem-pu@gmail.hu (M.C.); 2 Department of Genetics, Cell-and Immunobiology, Semmelweis University, Nagyvárad tér 4, 1089 Budapest, Hungary; takacs.angela@semmelweis.hu (A.T.); kohidai.laszlo@semmelweis.hu (L.K.); 3 Department Chemie, Ludwig-Maximilians-Universität München, Butenandtstr. 5-13, D-81377 München, Germany; klk@cup.uni-muenchen.de; 4 MS Proteomics Research Group, Research Centre for Natural Sciences, 1117 Budapest, Hungary

**Keywords:** α-hydroxy-alkylphosphonates, Pudovik reaction, acylation, sulfonylation, Arbuzov reaction, X-ray structure, cytotoxicity

## Abstract

A series of α-hydroxy-alkylphosphonates and α-hydroxy-alkylphosphine oxides were synthesized by the Pudovik reaction of acetaldehyde and acetone with dialkyl phosphites or diarylphosphine oxides. The additions were performed in three different ways: in liquid phase using triethylamine as the catalyst (1), on the surface of Al_2_O_3_/KF solid catalyst (2), or by a MW-assisted Na_2_CO_3_-catalyzed procedure (3). In most of the cases, our methods were more efficient and more robust than those applied in the literature. Two of the α-hydroxy-alkylphosphonates were subjected to single-crystal X-ray analysis, suggesting a dimeric and a chain supramolecular buildup in their respective crystals. Four α-hydroxy-alkylphosphonates and one α-hydroxy-ethylphosphine oxide were reacted with different acid chlorides to afford ten α-acyloxyphosphonates. Diethyl α-hydroxy-ethylphosphonate was transformed to the methanesulfonyloxy derivative that was useful in the Michaelis–Arbuzov reaction with triethyl phosphite and ethyl diphenylphosphinite to afford tetraethyl ethylidenebisphosphonate and diethyl α-(diphenylphosphinoyl)-ethylphosphonate, respectively. The α-hydroxyphosphonates and α-hydroxyphosphine oxides prepared were subjected to bioactivity studies, and the compounds tested exhibited limited cytotoxic effects on U266 cells with modest reductions in viability at a concentration of 100 μM.

## 1. Introduction

α-Hydroxyphosphonates and related derivatives are of importance for several reasons. This family of compounds may be easily synthesized by the Pudovik reaction invol-ving the addition of dialkyl phosphites or other >P(O)H reagents on the C=O group of oxo compounds, like aldehydes and ketones [1,2,3]. A wide choice of catalysts was described, typically including K_3_PO_4_, Ba(OH)_2_, MgO, Al_2_O_3_, Ti(O^i^Pr_4_), Na_2_CO_3_, MgCl_2_/Et_3_N, and Na-modified fluorapatite [4,5,6,7,8,9,10,11].

“Green” synthetic methods were also developed [12], such as microwave-assisted and/or solvent-free procedures [13]. The latter variation was performed on the surface of solid catalysts. However, the workup required a great quantity of different solvents due to extraction and purification by chromatography [14,15,16,17,18]. Keglevich and co-workers introduced a method that is indeed “green”. According to this, an equimolar mixture of benzaldehyde and dialkyl phosphite is refluxed in acetone in the presence of triethylamine as the catalyst. On cooling, the adduct crystallized out from the mixture [19].

Hydroxyphosphonates may be the subject of many kinds of reactions [2], including substitution at the α-carbon atom [20], the modification of the hydroxy function by acylation [21,22,23,24], phosphorylation [25], and sulfonylation [26]. 

The compounds under discussion and their derivatives may be of biological activity [27,28,29,30,31,32,33]. This is the consequence of their enzyme inhibitory properties. Some of them have antiviral [27], antibacterial [28], and antifungal [29] effects. They may also be used as insecticides [30] and pesticides [31]. In addition, a few of their derivatives are antioxidant [32,33]. Keglevich et al. investigated the anticancer activity of dibenzyl α-hydroxy-benzylphosphonates. It was found that a few representatives tested on the Mes-Sa parental and the Mes-Sa/Dx5 multidrug-resistant uterine sarcoma cell lines showed promising cytotoxic effects. Substituted α-hydroxy-benzylphosphonates, α-hydroxy-benzylphosphonic acids, and α-phosphinoyloxy-benzylphosphonates were tested against the Mes-Sa uterine sarcoma cell line. Cytotoxicity screening revealed that the dibenzyl α-hyd-roxy-benzylphosphonate and the dimethyl α-diphenylphosphinoyloxy-phosphonate were the most active compounds. They were toxic against the multidrug-resistant cell line. Furthermore, acyloxyphosphonates were tested against different tumor (breast, skin, prostate, colon and lung carcinomas, as well as melanoma and Kaposi’s sarcoma) cell lines. The benzoylated derivative showed higher anticancer activity. A few analogues were more toxic on multidrug-resistant cancer cells [34]. The *α*-hydroxy- and *α*-mesyloxy-3,5-di-*tert*-butylbenzylphosphonates showed significant cytostatic activity on human breast carcinoma and also on melanoma cell culture [26].

We aimed at the preparation of α-hydroxy-alkylphosphonates and their derivatives in order to make potentially bioactive species available.

## 2. Results and Discussion

### 2.1. Synthesis of α-Hydroxyphosphonates and α-Hydroxyphosphine Oxides

**Scheme 1:**
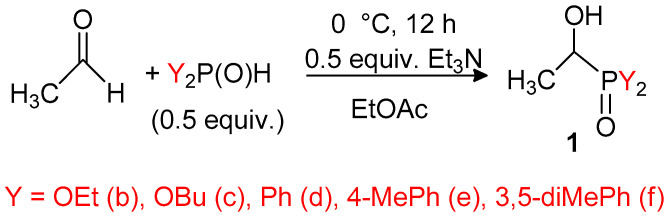
Synthesis of α-hydroxy-ethylphosphonates (**1b** and **1c**) and α-hydroxy-ethylphosphine oxides (**1d**–**f**).

In the first series of experiments, acetaldehyde used in a two-fold quantity due to its volatility was reacted with diethyl phosphite ((EtO)_2_P(O)H) and dibutyl phosphite ((BuO)_2_P(O)H) in the presence of 0.5 equiv. of triethylamine in ethyl acetate as the solvent (Scheme 1). After a 12 h’ stirring at 0 °C, the workup comprising purification by column chromatography gave α-hydroxy-ethylphosphonates **1b** and **1c** in yields of 93% and 76%, respectively. Acetaldehyde was also reacted with 0.5 equiv. of diarylphosphine oxides, such as diphenyl-, bis(4-methylphenyl)- and bis(3,5-dimethylphenyl)phosphine oxide, under the same conditions described above (Scheme 1). In these cases, the α-hydroxy-ethylphosphine oxides (**1d**–**f**) crystallized out from the mixture and were purified by recrystallization to afford products **1d**–**f** in 84–98% yields. 

Phosphonates **1b** and **1c**, along with phosphine oxides **1d**–**f** are known [35,36,37,38,39,40] compounds; **1f** was described by us [40]. These species were identified by ^31^P NMR and MS.

**Scheme 2:**
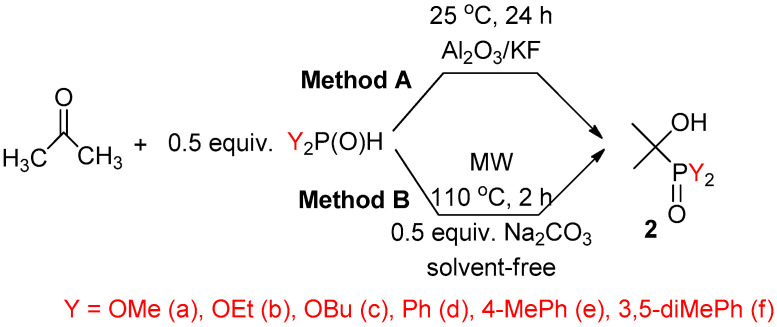
Preparation of α-hydroxy-α-methyl-ethylphosphonates (**2a**–**c**) and α-hydroxy-α-methyl-ethylphosphine oxides (**2d**–**f**).

Then, acetone also used in a two-fold quantity was involved in the Pudovik reaction with dimethyl, diethyl, and dibutyl phosphites on the surface of Al_2_O_3_/KF on the basis of analogies (Scheme 2, Method A) [41]. The workup comprised extraction followed by purification by column chromatography to provide α-hydroxy-α-methyl-ethylphosphonates **2a**–**c** in 65-76% yields. The acetone—secondary phosphine oxide adducts **2d**–**f** were prepared by the MW-assisted Na_2_CO_3_-catalyzed method elaborated by the Keglevich group earlier (Scheme 2, Method B) [13]. Product **2a**–**e** are known compounds [42,43,44,45,46], and were identified by ^31^P NMR and MS, while phosphine oxide **2f** is a new one; therefore, it was characterized by ^31^P, ^13^C, and ^1^H NMR, as well as MS. 

An earlier method for the preparation of diethyl α-hydroxy-ethylphosphonate (**1b**) started from ethyl alcohol applied as the solvent and the precursor for acetaldehyde at 80 °C using tetrabutyl hydroperoxide as the oxidant. The one-pot transformation also included the Pudovik reaction with (EtO)_2_P(O)H promoted by Na_2_CO_3_ and CuCl_2_ [35]. The outcome of the adduct (**1b**) was much lower (32%) than that from our variation (93%).

The dibutyl α-hydroxy-ethylphosphonate (**1c**) was synthesized from acetaldehyde and dibutyl phosphite in the presence of triethylamine on heating in a sealed tube. The yield of phosphonate **1c** was 54% [36] that was also lower than ours (76%). As regards the Pudovik addition of diphenylphosphine oxide (Ph_2_P(O)H) to acetaldehyde, this was described by converting the secondary phosphine oxide to the lithium salt by the reaction with n-butyl lithium at 0 °C or even below in tetrahydrofuran. In this way, the α-hydroxyethyl-diphenylphosphine oxide (**1d**) was obtained in 95% [37] and 45% [38] yields. The direct addition of Ph_2_P(O)H on the C=O group of acetaldehyde suggested by us is more practical. It is noted that the reaction of bis(4-methylphenyl)phosphine oxide with acetaldehyde was performed in water at 26 °C, applying the aldehyde in a 5 equiv.’ quantity. The corresponding adduct (**1e**) was isolated in 83% [39]. Our more direct method seems to be simpler. 

While we prepared the alkyl α-hydroxy-α-methyl-ethylphosphonates (**2a**, **2b,** and **2c**) on the surface of Al_2_O_3_/KF, the literature methods suggested liquid-phase accomplishments applying triethylamine, a hydrotalcite, or MgCl_2_ as the catalyst at 40–70 °C [42,43,44]. The yield for the dimethyl-, diethyl-, and dibutyl α-hydroxyphosphonates (**2a**, **2b**, and **2c**) was 88%, 68%, and 92%, respectively. Regarding the yields (65–73%), our method may be an alternative for the preparation of the P-esters under discussion. As regards the addition of secondary phosphine oxides on the C=O group of acetone, the addition of Ph_2_P(O)H was carried out on the surface of Al_2_O_3_/KF in an efficient way, as the corresponding product (**2d**) was obtained in 92% yield [45]. At the same time, the preparation of the acetone—bis(4-methylphenyl)phosphine oxide adduct (**2e**) could be performed in a low efficiency, in a yield of only 32% [46]. Our method applying Na_2_CO_3_ in a solvent-free manner under MW irradiation may be the method of choice furnishing adducts **2d** and **2e** in yields of 86% and 96%, respectively.

### 2.2. X-Ray Diffraction Studies of α-Hydroxyphosphonate ***2a*** and α-Hydroxyphosphine Oxide ***2d***

**Figure 1:**
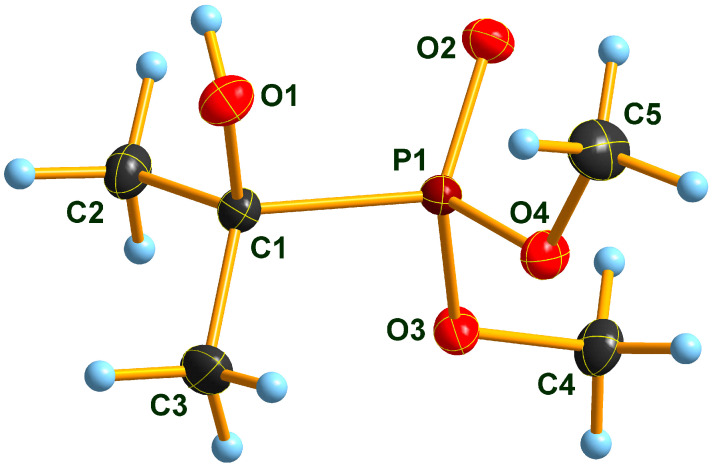
Molecular structure of hydroxyphosphonate **2a** in the crystal. DIAMOND [50] representation; thermal ellipsoids are drawn at 50% probability level.

**Figure 2:**
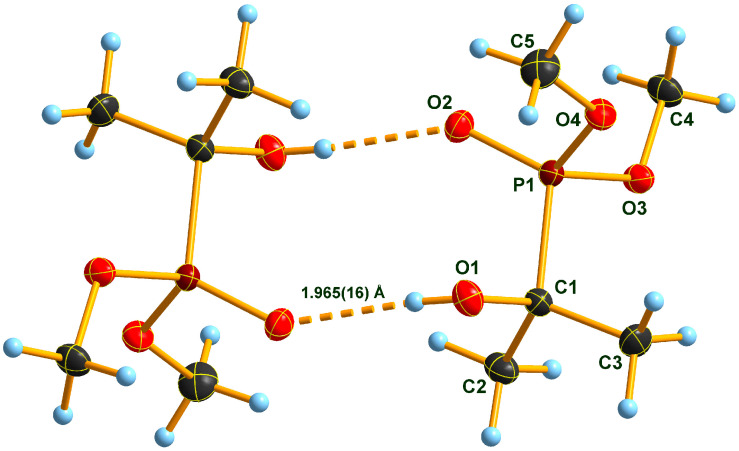
Crystal structure of hydroxyphosphonate **2a**; view of the hydrogen-bonded dimers in the crystal. DIAMOND [50] representation; thermal ellipsoids are drawn at 50% probability level. Symmetry code for the non-labeled molecule: 2 − x, −y, 1 − z.

**Figure 3:**
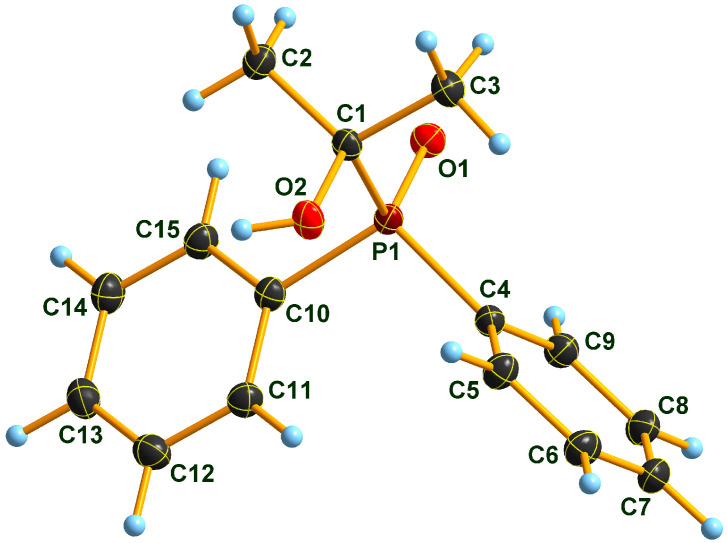
Molecular structure of hydroxyphosphine oxide **2d** in the crystal. DIAMOND [50] representation; thermal ellipsoids are drawn at 50% probability level.

**Figure 4:**
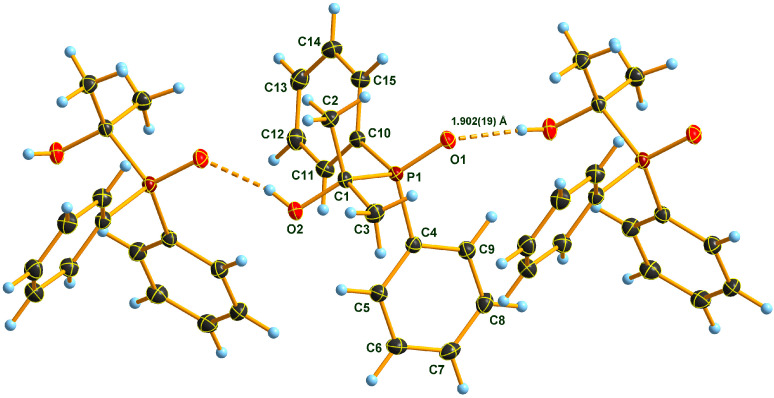
Crystal structure of hydroxyphosphine oxide **2d**. View of the hydrogen bonds in the crystal, resulting in the formation of chains. DIAMOND [50] representation; thermal ellipsoids are drawn at 50% probability level. Symmetry code for the left non-labeled molecule: x, 0.5 − y, −0.5 + z, and for the right non-labeled molecule: x, 0.5 − y, 0.5 + z.

X-ray diffraction provided information on the solid-state conformations and geometry of α-hydroxyphosphonate **2a** and α-hydroxyphosphine oxide **2d** in their single crystals (Figure 1, Figure 2, Figure 3 and Figure 4). The structure of α-hydroxyphosphine oxide **2d** has been published; the structural parameters of **2d** coincide well with those reported in the literature [47,48,49]. The structure of α-hydroxyphosphonate **2a** is to the best of our knowledge not yet described. Both derivatives **2a** and **2d** display usual and standard bond distances and angles (Figure 1 and Figure 3). The P=O bond and the OH unit are in a *syn*-clinal position in species **2a** (the HO–C–P==O, torsion angle is 60.8°), while the position of these functions is *anti*-periplanar in analog **2d** (the HO–C–P==O, torsion angle is 179.2°). This conformational difference may be related to the basic supramolecular hydrogen-bonding motif being a centrosymmetric dimer in hydroxyphosphonate **2a**, while hydroxyphosphine oxide **2d** forms “infinite” H-bridged chains (Figure 2 and Figure 4).

### 2.3. Modification of the α-Hydroxyphosphonates ***1b***, ***1c***, ***2b***, and ***2c*** and α-Hydroxyphosphine Oxide ***1d***

The α-hydroxy-alkylphosphonates (**1b**, **1c**, **2b**, and **2c**) and an α-hydroxy-ethylphosphine oxide (**1d**) were converted to the corresponding O-acyl derivatives (Table 1). The acetaldehyde—(EtO)_2_P(O)H adduct (**1b**) was reacted with 3 equiv. of acetyl chloride in toluene, in the presence of 1.5 equiv. of triethylamine at 25 °C for 1 day. The work-up followed by chromatography provided the O-acylated product (**3Ab**) in a yield of 65% (Table 1, entry 1). The acylation of phosphonate **1b** with propionyl chloride, butyryl chloride, and benzoyl chloride was performed similarly, but only 1.5 equiv. of the acid chloride was used, and at 25 °C, the reaction time was 3 days to afford the corresponding products **3Bb**, **3Cb**, and **3Db**, respectively, in yields of 67–73% (Table 1, entries 2–4). Modification of the acetaldehyde—(BuO)_2_P(O)H adduct (**1c**) and the phosphine oxide (**1d**) with 3 equiv. of acetyl chloride was carried out at 25 °C for 2 days, and at 60 °C for 3 days, respectively, to furnish products **3Ac** and **3Ad**, in yields of 65% and 85%, respectively (Table 1, entries 5 and 6). 

The acetone—(EtO)_2_P(O)H adduct (**2b**) was subjected to acylation with CH_3_C(O)Cl, C_2_H_5_-C(O)Cl, and C_3_H_7_-C(O)Cl similarly, but a higher temperature of 60 °C had to be applied to compensate the lower reactivity of hydroxyphosphonate **2b**, as compared to that of starting material **1b** (Table 1, entries 7–9). The lower reactivity of adduct **2b** is mainly the consequence of steric hindrance due to the extra methyl group. Products **4Ab**, **4Bb**, and **4Cb** were obtained in yields of 62-88%. The acetone—(BuO)_2_P(O)H adduct (**2c**) was (again) less reactive toward CH_3_C(O)Cl; therefore, the time of heating was 3 days (Table 1, entry 10).

All acylated products were purified by column chromatography. As acyloxyphosphonates **3Ab** and **3Db** are known compounds [51,52], they were identified by ^31^P NMR and MS. New species **3Bb**, **3Cb**, **3Ac**, **3Ad**, **4Ab**, **4Bb**, **4Cb**, and **4Ac** were characterized by ^31^P, ^13^C, and ^1^H NMR, as well as HRMS.

**Scheme 3:**
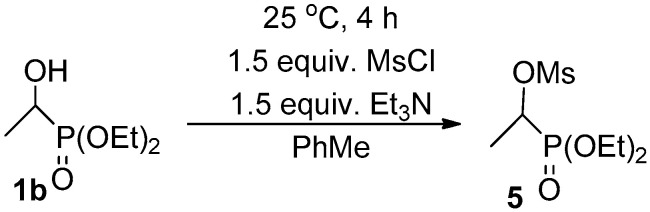
Preparation of α-mesyloxy-ethylphoshonate (**5**).

Then, to prepare suitable starting material for the Michaelis–Arbuzov reaction, the diethyl α-hydroxy-ethylphosphonate **1b** was converted to the sulfonyloxy derivative (**5**) (Scheme 3). Using 1.5 equiv. of methanesulfonyl chloride in toluene together with 1.5 equiv. of triethylamine at room temperature, the reaction time was 4 h. Product **5** was isolated in a 69% yield and was characterized.

The methanesulfonyloxy-ethylphosphonate **5** was then taken in the Michaelis–Arbuzov reaction with triethyl phosphite ((EtO)_3_P). Using the reagent in a five-fold quantity at 135 °C for 2 days, the conversion toward tetraethyl bisphosphonate **6a** was only 25% (Table 2, entry 1). Applying 9 equiv. of the P-reagent at 150 °C for 3 days, the conversion was improved to 50% (Table 2, entry 2). In the presence of 10% of NiCl_2_, the conversion was 62% (Table 2, entry 3). Extending the reaction time to 6 days did not result in a significant increase in conversion either with or without 10% NiCl_2_ catalyst. The conversions were 66 and 70%, respectively (Table 2, entry 4). In our previous work, mesyloxy-benzylphosphonates were subjected to the Arbuzov reaction with (EtO)_3_P, where the desired bisphosphonates were formed in a complete conversion within 3–4 days at 135 °C. It can be seen that the reactivity of mesyloxy-alkylphosphonates in the Arbuzov reaction is lower than that of the benzyl derivatives. Ethyl diphenylphosphinite was more reactive, as using it in a 5 equiv.’ quantity at 135 °C for 1 day, the conversion to phosphonate—phosphine oxide **6b** was 40% (Table 2, entry 5). In the presence of 10% of NiCl_2_ at 150 °C for 2 days, the conversion was 70% (Table 2, entry 6). Complete conversion was achieved after 3 days at 150 °C without the catalyst.

The crude products of the reactions that resulted in the best conversion were purified by column chromatography to obtain bisphosphonic derivatives (**6a** and **6b**) in yields of 50 and 75%, respectively (Table 2, entries 4 and 7). As they are known [53] compounds, they were identified by ^31^P NMR and MS.

### 2.4. Bioactivity Study

**Figure 5:**
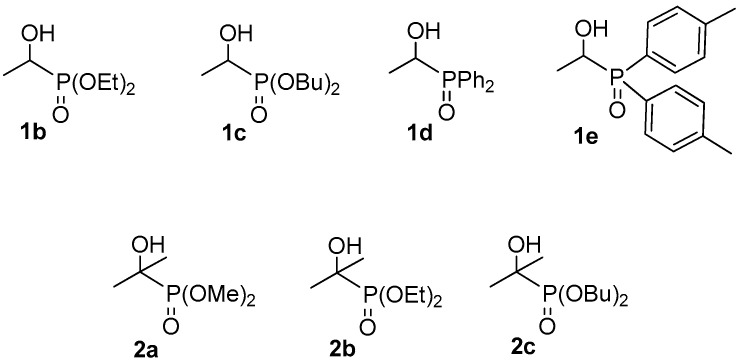
Hydroxyphosphonate derivatives (**1b**–**e** and **2a**–**c**) subjected to bioactivity study.

Hydroxyphosphonates **1b**, **1c**, and **2a**–**c**, as well as hydroxyphosphine oxides **1d** and **1e** were subjected to bioactivity study (Figure 5).

The cell viability assays for PANC-1 and U266 cell lines revealed that most compounds maintained viabilities close to 1.0 for all three concentrations, indicating low toxicity (Table 3). For U266 cells, greater variability was observed, particularly at 100 μM, where phosphonates **1b** and **2b** and phosphine oxide **2b** were somewhat toxic on this cell line, resulting in a reduced viability of 0.77–0.95. Hydroxyphosphonate **2b** displayed the highest toxicity marked by a cell death of 23%.

## 3. Experimental Section

### 3.1. General Information

The MW reactions were carried out in a CEM Discover (300 W) focused microwave reactor (CEM Microwave Technology Ltd., Buckingham, UK) equipped with a stirrer and a pressure controller using 80–100 W irradiation under isothermal conditions. The reaction mixtures were irradiated in sealed glass vessels (with a volume of 10 mL) available from the supplier of CEM. The reaction temperature was monitored by an external IR sensor.

The ^31^P, ^13^C, and ^1^H NMR spectra were measured on a Bruker DRX-500 or Bruker Avance-300 spectrometer operating at 202, 126, and 500 MHz or 122, 75, and 300 MHz, respectively (Bruker, Billerica, MA, USA). The couplings are given in Hz. The copies of the ^31^P, ^13^C and ^1^H NMR spectra for compounds **1b-f**, **2a-f**, **3Ab-Db**, **3Ac**, **3Ad**, **4Ab-Cb**, **4Ac**, **5**, **6a** and **6b** prepared can be seen in the Supplementary Materials. LC–MS measurements were performed with an Agilent 1200 liquid chromatography system, coupled with a 6130 quadrupole mass spectrometer equipped with an ESI ion source (Agilent Technologies, Palo Alto, CA, USA). High-resolution mass spectrometric measurements were performed using a Thermo (Waltham, MA, USA) Velos Pro Orbitrap Elite hybrid mass spectrometer with an ESI ion source in positive electrospray mode.

### 3.2. General Procedure for the Synthesis of Dialkyl α-Hydroxy-Ethylphosphonates (***1b*** and ***1c***) and α-Hydroxyethyl-Diarylphosphine Oxides (***1d***–***f***)

A mixture of 11.0 mmol (0.38 mL) of acetaldehyde, 5.5 mmol of dialkyl phosphite (diethyl phosphite: 0.71 mL, dibutyl phosphite: 1.1 mL) or 5.5 mmol of diarylphosphine oxide (diphenylphosphine oxide: 1.1 g, bis(4-methylphenyl)phosphine oxide: 1.3 g, bis(3,5-dimethylphenyl)phosphine oxide: 1.4 g) and 5.5 mmol (0.77 mL) of triethylamine was stirred in 5 mL of ethyl acetate at 0 °C for 12 h. In the case of products **1b** and **1c**, the solvent was evaporated under vacuum, and the crude product was purified by column chromatography (using ethyl acetate as the eluent on silica gel). Hydroxyphosphine oxides **1d**–**f** crystallized out from the reaction mixture. The solids were removed by filtration and purified by recrystallization from acetone. Products **1b** and **1c** are pale yellow oils, while hydroxyphosphine oxides **1d**–**f** are white crystalline compounds.

#### 3.2.1. Diethyl α-Hydroxy-Ethylphosphonate (**1b**)

Yield: 0.93 g (93%); pale yellow oil; ^31^P {^1^H} NMR (122 MHz, CDCl_3_) δ 25.9; δ_P,lit._ [35] 27.9; [M + H]^+^ = 183; HRMS (ESI) *m*/*z*: [M + Na]^+^ calculated for C_6_H_15_O_4_PNa 205.0606; found 205.0606.

#### 3.2.2. Dibutyl α-Hydroxy-Ethylphosphonate (**1c**)

Yield: 0.99 g (76%); pale yellow oil; ^31^P {^1^H} NMR (202 MHz, CDCl_3_) δ 25.8; δ_P,lit._ [36] 25.8; [M + H]^+^ = 239; HRMS (ESI) *m*/*z*: [M + Na]^+^ calculated for C_10_H_23_O_4_PNa 261.1232; found 261.1227.

#### 3.2.3. α-Hydroxyethyl-Diphenylphosphine Oxide (**1d**)

Yield: 1.3 g (97%); white crystals; m.p.: 130–131 °C; m.p. [37] 131–132 °C; ^31^P {^1^H} NMR (202 MHz, CDCl_3_) δ 33.2; δ_P,lit._ [38] 32.9; [M + H]^+^ = 247; HRMS (ESI) *m/z*: [M + Na]^+^ calculated for C_14_H_15_O_2_PNa 269.0707; found 269.0704. [M + H]^+^ calculated for C_14_H_16_O_2_P 247.0888; found 247.0878.

#### 3.2.4. α-Hydroxyethyl-bis(4-methylphenyl)phosphine Oxide (**1e**)

Yield: 1.3 g (84%); white crystals; m.p.: 100–101 °C; m.p. [39] 99.8–102.1 °C; ^31^P {^1^H} NMR (202 MHz, CDCl_3_) δ 33.8; δ_P,lit._ [39] 33.3; [M + H]^+^ = 275; HRMS (ESI) *m*/*z*: [M + Na]^+^ calculated for C_16_H_19_O_2_PNa 297.1020; found 297.1018; [M + H]^+^ calculated for C_16_H_20_O_2_PNa 275.1201; found 275.1198.

#### 3.2.5. α-Hydroxyethyl-bis(3,5-dimethylphenyl)phosphine Oxide (**1f**)

Yield: 1.6 g (98%); white crystals; m.p.: 192–193 °C; ^31^P {^1^H} NMR (202 MHz, CDCl_3_) δ 35.5; δ_P,lit._ [40] 35.5; [M + H]^+^ = 303; HRMS (ESI) *m*/*z*: [M + Na]^+^ calculated for C_18_H_23_O_2_PNa 325.1333; found 325.1330.

### 3.3. General Procedure for the Synthesis of Dialkyl α-Hydroxy-α-Methyl-Ethylphosphonates (***2a***–***c***) (Method A)

To 11.0 mmol (0.81 mL) of acetone and 5.5 mmol of dialkyl phosphite (dimethyl phosphite: 0.50 mL, diethyl phosphite: 0.70 mL, dibutyl phosphite: 1.0 mL), a mixture of finely powdered, 1.3 g of acidic Al_2_O_3_ (Brockmann I.) and 1.3 g of potassium fluoride was added. The reaction mixture was allowed to stand at 26 °C for 24 h. The product was extracted from the solid phase with 4 × 25 mL of ethyl acetate. The solvent was evaporated, and the crude product so obtained purified by column chromatography on silica gel applying ethyl acetate as the eluent. Compound **2a** was obtained as white crystals, while product **2b** and **2c** were oils.

#### 3.3.1. Dimethyl α-Hydroxy-α-Methyl-Ethylphosphonate (**2a**)

Yield: 1.1 g (65%); white crystals; m.p.: 73–74 °C; m.p. [42] 68.6–70.2 °C; ^31^P {^1^H} NMR (202 MHz, CDCl_3_) δ 29.7; δ_P,lit._ [42] 30.3 (dmso-d_6_); [M + H]^+^ = 169; HRMS (ESI) *m*/*z*: [M + Na]^+^ calculated for C_5_H_13_O_4_PNa 191.0449; found 191.0447.

#### 3.3.2. Diethyl α-Hydroxy-α-Methyl-Ethylphosphonate (**2b**)

Yield: 1.4 g (71%); colorless oil; ^31^P {^1^H} NMR (202 MHz, CDCl_3_) δ 27.6; δ_P,lit._ [43] 27.4; [M + H]^+^ = 197; HRMS (ESI) *m*/*z*: [M + Na]^+^ calculated for C_7_H_17_O_4_PNa 219.0762; found 219.0759.

#### 3.3.3. Dibutyl α-Hydroxy-α-Methyl-Ethylphosphonate (**2c**)

Yield: 1.9 g (76%); colorless oil; ^31^P {^1^H} NMR (202 MHz, CDCl_3_) δ 27.5; δ_P,lit._ [44] 27.3; [M + H]^+^ = 253; HRMS (ESI) *m*/*z*: [M + Na]^+^ calculated for C_11_H_25_O_4_PNa 275.1388; found 275.1391.

### 3.4. General Procedure for the Synthesis of Diaryl α-Hydroxy-α-Methyl-Ethylphosphine Oxides (***2d***–***f***) (Method B)

A mixture of 2.0 mmol (0.15 mL) of acetone, 1.0 mmol of diarylphosphine oxide (diphenylphosphine oxide: 0.21 g, bis(4-methylphenyl)phosphine oxide: 0.23 g, bis(3,5-dimethylphenyl)phosphine oxide: 0.26 g) and 1.0 mmol (0.11 g) of Na_2_CO_3_ was heated at 110 °C in a vial in a CEM Discover Microwave reactor for 2 h. The reaction mixture was extracted with 20 mL of ethyl acetate. The organic phase was evaporated, and the crude product so obtained purified by recrystallization from acetone. Products **2d**–**f** were obtained as white crystalline compounds.

#### 3.4.1. α-Hydroxy-α-Methyl-Ethyl-Diphenylphosphine Oxide (**2d**)

Yield: 0.22 g (86%); white crystals; m.p.: 136–137 °C; m.p. [45] 136–138 °C; ^31^P {^1^H} NMR (202 MHz, CDCl_3_) δ 34.5; δ_P,lit._ [45] 34.6; [M + H]^+^ = 261; HRMS (ESI) *m*/*z*: [M + Na]^+^ calculated for C_15_H_17_O_2_PNa 283.0864; found 283.0860.

#### 3.4.2. Bis(4-Methylphenyl)(α-Hydroxy-α-Methyl-Ethyl)Phosphine Oxide (**2e**)

Yield: 0.28 g (96%); white crystals; m.p.: 122–123 °C; m.p. [46] 125.2–125.6; ^31^P {^1^H} NMR (202 MHz, CDCl_3_) δ 35.3; δ_P,lit._ [46] 34.9; [M + H]^+^ = 289; HRMS (ESI) *m*/*z*: [M + Na]^+^ calculated for C_17_H_21_O_2_PNa 311.1177; found 311.1170.

#### 3.4.3. Bis(3,5-Dimethylphenyl)(α-Hydroxy-α-Methyl-Ethyl)Phosphine Oxide (**2f**)

Yield: 0.30 g (94%); white crystals; m.p.: 112–113 °C; ^31^P {^1^H} NMR (202 MHz, CDCl_3_) δ 34.9; ^13^C {^1^H} NMR (126 MHz, CDCl_3_) δ 21.3 (s, ArCH_3_), 25.3 (d, *J* = 6.9 Hz, C*C*H_3_), 72.1 (d, *J* = 86.8 Hz, CP), 129.9 (d, *J* = 8.2 Hz, C_β_), 130.2 (d, *J* = 89.9 Hz, C_α_), 133.4 (d, *J* = 2.6 Hz, C_δ_), 137.7 (d, *J* = 11.5 Hz, C_γ_); ^1^H NMR (500 MHz, CDCl_3_) δ 1.52 (d, *J* = 14.1 Hz, 6H, CCH_3_), 2.19 (s, 1H, OH), 2.39 (s, 12H, ArCH_3_), 7.23 (s, 2H, ArH_δ_), 7.61 (d, *J* = 10.9 Hz, 4H, ArH_β_); [M + H]^+^ = 317; HRMS (ESI) *m*/*z*: [M + Na]^+^ calculated for C_19_H_25_O_2_PNa 339.1498; found 339.1490.

### 3.5. General Procedure for the Synthesis of Acylated Diethyl and Dibutyl a-Hydroxyphosphonates and Diphenyl α-Hydroxyphosphine Oxide (***3Ab***–***Db***, ***3Ac***, ***3Ad***, ***4Ab***–***Cb***, and ***4Ac***)

To 1.2 mmol of α-hydroxyphosphonate (**1b**: 0.22 g, **1c**: 0.29 g, **1d**: 0.30 g, **2b**: 0.24 g, **2c**: 0.30 g), and 1.8 mmol (0.25 mL) of triethylamine in toluene (4.0 mL), 3.6 mmol (0.26 mL) of acetyl chloride, or 1.8 mmol of other acyl chlorides (propionyl chloride: 0.16 mL, butyryl chloride: 0.19 mL, benzoyl chloride: 0.21 mL) were added, and the mixture was kept at 25–60 °C for 1–3 days (See Table 1) in a sealed tube. The precipitated triethylamine hydrochloride was filtered off, and the volatile components were removed in vacuum. The crude product so obtained was purified by column chromatography on silica gel app-lying dichloromethane–methanol (97:3) as the eluent to give products **3Ab**–**Db**, **3Ac**, **3Ad**, **4Ab**–**Cb**, and **4Ac** in yields of 61–88% as oils.

#### 3.5.1. Diethyl α-Acetyloxy-Ethylphosphonate (**3Ab**)

Yield: 0.18 g (65%); pale yellow oil; ^31^P {^1^H} NMR (202 MHz, CDCl_3_) δ 21.4; δ_P,lit._ [51] 21.1; [M + H]^+^ = 225; HRMS (ESI) *m*/*z*: [M + Na]^+^ calculated for C_8_H_17_O_5_PNa 247.0711; found 247.0717.

#### 3.5.2. Diethyl α-Propionyloxy-Ethylphosphonate (**3Bb**)

Yield: 0.21 g (73%); pale yellow oil; ^31^P {^1^H} NMR (202 MHz, CDCl_3_) δ 21.6; ^13^C {^1^H} NMR (126 MHz, CDCl_3_) δ 9.0 (s, CH_2_*C*H_3_), 15.1 (s, CH*C*H_3_), 16.3 and 16.4 (d, *J* = 5.7 Hz, CH_2_*C*H_3_), 27.5 (s, *C*H_2_CH_3_), 62.6 and 62.8 (d, *J* = 6.6 Hz, OCH_2_), 64.2 (d, *J* = 171.5 Hz, CH), 173.1 (d, *J* = 7.6 Hz, C=O); ^1^H NMR (500 MHz, CDCl_3_) δ 1.16 (t, *J* = 7.6 Hz, 3H, CH_2_C*H*_3_), 1.33 and 1.34 (overlapped t, *J* = 6.9 Hz, 6H, CH_2_C*H*_3_), 1.46 (dd, *J*_1_ = 16.8 Hz, *J*_2_ = 7.1 Hz, 3H, CHC*H*_3_), 2.37–2.41 (m, 2H, C*H*_2_CH_3_), 4.13–4.21 (m, 4H, OCH_2_), 5.27–5.30 (m, 1H, CH); [M + H]^+^ = 239; HRMS (ESI) *m*/*z*: [M + Na]^+^ calculated for C_9_H_19_O_5_PNa 261.0868; found 261.0868.

#### 3.5.3. Diethyl α-Butyryloxy-Ethylphosphonate (**3Cb**)

Yield: 0.20 g (67%); pale yellow oil; ^31^P {^1^H} NMR (202 MHz, CDCl_3_) δ 21.6; ^13^C {^1^H} NMR (126 MHz, CDCl_3_) δ 13.5 (s, CH_2_*C*H_3_), 15.1 (s, CH*C*H_3_), 16.3 and 16.4 (d, *J* = 5.7 Hz, CH_2_*C*H_3_), 18.4 (s, *C*H_2_CH_3_) 36.0 (s, *C*H_2_CH_2_), 62.6 and 62.7 (d, *J* = 6.6 Hz, OCH_2_), 64.1 (d, *J* = 171.4 Hz, CH), 172.3 (d, *J* = 7.5 Hz, C=O); ^1^H NMR (500 MHz, CDCl_3_) δ 0.96 (t, *J* = 7.4 Hz, 3H, CH_2_C*H*_3_), 1.33 and 1.34 (overlapped t, *J* = 7.1 Hz, 6H, CH_2_C*H*_3_), 1.46 (dd, *J*_1_ = 16.8 Hz, *J*_2_ = 7.1 Hz, 3H, CHC*H*_3_), 1.64–1.71 (m, 2H, C*H*_2_CH_3_) 2.35 (t, *J* = 7.4 Hz, 2H, C(O)CH_2_), 4.13–4.20 (m, 4H, OCH_2_), 5.26–5.32 (m, 1H, CH); [M + H]^+^ = 253; HRMS (ESI) *m*/*z*: [M + Na]^+^ calculated for C_10_H_21_O_5_PNa 275.1024; found 275.1026.

#### 3.5.4. Diethyl α-Benzoyloxy-Ethylphosphonate (**3Db**)

Yield: 0.23 g (68%); pale yellow oil; ^31^P {^1^H} NMR (202 MHz, CDCl_3_) δ 21.4; δ_P,lit._ [52] 21.4; [M + H]^+^ = 287; HRMS (ESI) *m*/*z*: [M + Na]^+^ calculated for C_13_H_19_O_5_PNa 309.0868; found 309.0870.

#### 3.5.5. Dibutyl α-Acetyloxy-Ethylphosphonate (**3Ac**)

Yield: 0.15 g (65%); pale yellow oil; ^31^P {^1^H} NMR (202 MHz, CDCl_3_) δ 21.5; ^13^C {^1^H} NMR (126 MHz, CDCl_3_) δ 13.5 (s, CH_2_*C*H_3_), 15.1 (s, CH*C*H_3_), 18.6 (s, *C*H_2_CH_3_), 20.8 (s, C(O)*C*H_3_), 32.4 and 32.5 (d, *J* = 5.8 Hz, OCH_2_*C*H_2_), 64.4 (d, *J* = 172.1 Hz, CH), 66.4 and 66.6 (d, *J* = 6.9 Hz, OCH_2_), 169.6 (d, *J* = 8.1 Hz, C=O); ^1^H NMR (500 MHz, CDCl_3_) δ 0.96 (t, *J* = 7.4 Hz, 6H, CH_2_C*H*_3_), 1.38–1.44 (m, 4H, C*H*_2_CH_3_), 1.48 (dd, *J*_1_ = 16.7 Hz, *J*_2_ = 7.1 Hz, 3H, CHC*H*_3_), 1.64–1.71 (m, 4H, OCH_2_C*H*_2_), 2.13 (s, 3H, C(O)CH_3_), 4.09–4.14 (m, 4H, OCH_2_), 5.26–5.32 (m, 1H, C*H*CH_3_); [M + H]^+^ = 281; HRMS (ESI) *m*/*z*: [M + H]^+^ calculated for C_12_H_26_O_5_P 281.1513; found 281.1520.

#### 3.5.6. Diphenyl α-Acetyloxy-Ethylphosphine Oxide (**3Ad**)

Yield: 0.29 g (85%); pale yellow oil; ^31^P {^1^H} NMR (202 MHz, CDCl_3_) δ 31.4; ^13^C {^1^H} NMR (126 MHz, CDCl_3_) δ 14.0 (s, CH*C*H_3_), 20.7 (s, C(O)*C*H_3_), 67.2 (d, *J* = 88.9 Hz, CH), 128.58 and 130.0 (d, *J* = 99.4 Hz, C_α_), 128.6 and 128.8 (d, *J* = 11.8 Hz, C_β_), 131.2 and 131.8 (d, *J* = 9.4 Hz, C_γ_), 132.40 and 132.43 (d, *J* = 1.3 Hz, C_δ_), 169.5 (d, *J* = 6.7 Hz, C=O); ^1^H NMR (500 MHz, CDCl_3_) δ 1.50 (dd, *J*_1_ = 14.4 Hz, *J*_2_ = 7.1 Hz, 3H, CHC*H*_3_), 1.93 (s, 3H, C(O)CH_3_), 5.86–5.90 (m, 1H, C*H*CH_3_); 7.49–7.63, 7.77–7.81 and 7.89–7.93 (m, 10H, ArH); [M + H]^+^ = 289; HRMS (ESI) *m/z*: [M + H]^+^ calculated for C_16_H_18_O_3_P 289.0989; found 289.0975.

#### 3.5.7. Diethyl (α-Acetyloxy-α-Methyl-Ethyl)Phosphonate (**4Ab**)

Yield: 0.18 g (62%); pale yellow oil; ^31^P {^1^H} NMR (202 MHz, CDCl_3_) δ 23.1; ^13^C {^1^H} NMR (126 MHz, CDCl_3_) δ 16.4 (d, *J* = 5.5 Hz, CH_2_*C*H_3_), 21.6 (s, C(O)*C*H_3_), 22.2 (s, C*C*H_3_), 63.1 (d, *J* = 7.0 Hz, OCH_2_), 79.4 (d, *J* = 174.4 Hz, CP), 169.7 (d, *J* = 14.1 Hz, C=O); ^1^H NMR (500 MHz, CDCl_3_) δ 1.37 (t, *J* = 7.1 Hz, 6H, CH_2_C*H*_3_), 1.77 (d, *J* = 15.9 Hz, 6H, CCH_3_), 2.07 (s, 3H, C(O)CH_3_), 4.20–4.26 (m, 4H, OCH_2_); [M + H]^+^ = 239; HRMS (ESI) *m*/*z*: [M + H]^+^ calculated for C_9_H_20_O_5_P 239.1043; found 239.1052.

#### 3.5.8. Diethyl (α-Propionyloxy-α-Methyl-Ethyl)Phosphonate (**4Bb**)

Yield: 0.19 g (62%); pale yellow oil; ^31^P {^1^H} NMR (202 MHz, CDCl_3_) δ 23.2; ^13^C {^1^H} NMR (126 MHz, CDCl_3_) δ 8.9 (s, CH_2_*C*H_3_), 16.5 (d, *J* = 5.6 Hz, CH_2_*C*H_3_), 21.6 (s, C*C*H_3_), 28.5 (s, C(O)*C*H_2_), 63.1 (d, *J* = 7.1 Hz, OCH_2_), 79.2 (d, *J* = 174.4 Hz, CP), 173.3 (d, *J* = 14.0 Hz, C=O); ^1^H NMR (500 MHz, CDCl_3_) δ 1.14 (t, *J* = 7.6 Hz, 3H, CH_2_C*H*_3_), 1.37 (t, *J* = 7.1 Hz, 6H, CH_2_C*H*_3_), 1.77 (d, *J* = 15.9 Hz, 6H, CCH_3_), 2.32–2.37 (m, 2H, C(O)CH_2_), 4.20–4.25 (m, 4H, OCH_2_); [M + H]^+^ = 253; HRMS (ESI) *m*/*z*: [M + H]^+^ calculated for C_10_H_22_O_5_P 253.1200; found 253.1204.

#### 3.5.9. Diethyl (α-Butyryloxy-α-Methyl-Ethyl)Phosphonate (**4Cb**)

Yield: 0.28 g (88%); pale yellow oil; ^31^P {^1^H} NMR (202 MHz, CDCl_3_) δ 23.2; ^13^C {^1^H} NMR (126 MHz, CDCl_3_) δ 13.5 (s, CH_2_*C*H_3_), 16.5 (d, *J* = 5.6 Hz, CH_2_*C*H_3_), 18.4 (s, *C*H_2_CH_3_), 21.6 (s, C*C*H_3_), 37.1 (s, C(O)*C*H_2_), 63.1 (d, *J* = 7.2 Hz, OCH_2_), 79.1 (d, *J* = 174.1 Hz, CP), 172.4 (d, *J* = 13.8 Hz, C=O); ^1^H NMR (500 MHz, CDCl_3_) δ 0.88 (t, *J* = 7.4 Hz, 3H, CH_2_C*H*_3_), 1.28 (t, *J* = 7.1 Hz, 6H, CH_2_C*H*_3_), 1.53–1.63 (m, 2H, C*H*_2_CH_3_), 1.68 (d, *J* = 15.9 Hz, 6H, CCH_3_), 2.21 (t, *J* = 7.4 Hz, 2H, C(O)CH_2_), 4.10–4.16 (m, 4H, OCH_2_); [M + H]^+^ = 267; HRMS (ESI) *m*/*z*: [M + H]^+^ calculated for C_11_H_24_O_5_P 267.1356; found 267.1366.

#### 3.5.10. Dibutyl (α-Acetyloxy-α-Methyl-Ethyl)Phosphonate (**4Ac**)

Yield: 0.22 g (61%); pale yellow oil; ^31^P {^1^H} NMR (202 MHz, CDCl_3_) δ 22.9; ^13^C {^1^H} NMR (126 MHz, CDCl_3_) δ 13.6 (s, CH_2_*C*H_3_), 18.7 (s, *C*H_2_CH_3_), 21.6 (s, C(O)*C*H_3_), 22.2 (s, C*C*H_3_) 32.6 (d, *J* = 5.8 Hz, OCH_2_*C*H_2_), 66.7 (d, *J* = 7.3 Hz, OCH_2_), 79.6 (d, *J* = 174.7 Hz, CP), 169.8 (d, *J* = 14.3 Hz, C=O); ^1^H NMR (500 MHz, CDCl_3_) δ 0.97 (t, *J* = 7.4 Hz, 6H, CH_2_C*H*_3_), 1.40–1.48 (m, 4H, C*H*_2_CH_3_), 1.67–1.72 (m, 4H, OCH_2_C*H*_2_), 1.77 (d, J = 15.9 Hz, 6H, CCH_3_), 2.06 (s, 3H, C(O)CH_3_), 4.13–4.17 (m, 4H, OCH_2_); [M + H]^+^ = 295; HRMS (ESI) *m*/*z*: [M + H]^+^ calculated for C_13_H_28_O_5_P 295.1669; found 295.1676.

### 3.6. Synthesis of Diethyl α-Methanesulfonyloxy-Ethylphosphonate (***5***)

A mixture of 1.0 mmol (0.21 g) of diethyl *α*-hydroxy-ethylphosphonate, 1.5 mmol (0.12 mL) of methanesulfonyl chloride, and 1.5 mmol (0.21 mL) of triethylamine in 5 mL of toluene was stirred at room temperature for 4 h. The precipitated triethylamine hydrochloride salt was filtered off, the filtrate was evaporated under vacuum, and the crude product so obtained purified by column chromatography (using DCM−MeOH 97:3 as the eluent on silica gel). The product was obtained as a yellow oil. 

Yield: 0.18 g (69%); yellow oil; ^31^P {^1^H} NMR (202 MHz, CDCl_3_) δ 18.2; ^13^C {^1^H} NMR (126 MHz, CDCl_3_) δ 16.4 (t, *J* =5.5 Hz, CH_2_*C*H_3_), 16.7 (s, CH*C*H_3_), 38.9 (s, SCH_3_), 63.3 (d, *J* = 7.3 Hz, OCH_2_), 72.3 (d, *J* = 172.1 Hz, CH); ^1^H NMR (500 MHz, CDCl_3_) δ 1.39 (t, *J* = 7.1 Hz, 6H, CH_2_C*H*_3_), 1.63 and 1.67 (d, *J* = 7.1 Hz, 3H, CHC*H*_3_), 4.20–4.26 (m, 4H, OCH_2_), 4.91–4.97 (m, 1H, CH); [M + H]^+^ = 261; HRMS *m*/*z*: [M + H]^+^ calculated for C_7_H_18_O_6_PS 261.0557; found 261.0557.

### 3.7. Synthesis of Tetraethyl Ethylidenebisphosphonate (***6a***) and Diethyl α-(Diphenylphosphinoyl)-Ethylphosphonate (***6b***)

A mixture of 1.0 mmol (0.26 g) of diethyl α-methanesulfonyloxy-ethylphosphonate (**5**), 9.0 mmol (1.5 mL) of triethyl phosphite, or 5.0 mmol (1.1 mL) of ethyl diphenylphosphinite was stirred at 150 °C for 3–6 days in a sealed tube (for the details see Table 2). The crude product was purified by column chromatography (using DCM–MeOH 97:3 as the eluent on silica gel).

The following products were thus prepared:

#### 3.7.1. Tetraethyl Ethylidenebisphosphonate (**6a**)

Yield: 0.15 g (50%); colorless oil; ^31^P {^1^H} NMR (202 MHz, CDCl_3_) δ 24.0; δ_P,lit._ [53] 21.4; [M + H]^+^ = 303; HRMS (ESI) *m*/*z*: [M + H]^+^ calculated for C_10_H_25_O_6_P_2_ 303.1121; found 303.1121.

#### 3.7.2. Diethyl α-(Diphenylphosphinoyl)-Ethylphosphonate (**6b**)

Yield: 0.27 g (75%); colorless oil; ^31^P {^1^H} NMR (202 MHz, CDCl_3_) δ 24.2 and 30.1 (d, *J* = 5.5 Hz); δ_P,lit_ [53] 21.5 and 26.5; [M + H]^+^ = 367; HRMS (ESI) *m*/*z*: [M + H]^+^ calculated for C_18_H_25_O_4_P_2_ 367.1223; found 367.1227.

### 3.8. Single-Crystal X-Ray Diffraction Studies

The single crystals of compounds **2a** and **2d**, suitable for X-ray diffraction, were obtained by the slow evaporation of CDCl_3_ (**2a**) or acetone (**2d**) solution. The crystals were introduced into perfluorinated oil, and a suitable single crystal was carefully mounted on the top of a thin glass wire. Data collection was performed with an Oxford Xcalibur 3 diffractometer equipped with a Spellman generator (50 kV, 40 mA) and a Kappa CCD detector, operating with Mo-K_α_ radiation (λ = 0.71071 Ǻ).

Data collection and data reduction were performed with the CrysAlisPro version 1.171.40.82a software [54]. Absorption correction using the multiscan method [54] was applied. The structures were solved with the SHELXS-97 Program for Crystal Structure Solution [55], refined with the SHELXL-97 Program for the Refinement of Crystal Structures [56] and finally checked using PLATON [57]. The structure was depicted with the DIAMOND evaluation program [50]. Details for data collection and structure refinement are summarized in Table 4. CCDC-2408109 (**2a**) and CCDC-2408108 (**2d**) contain supplementary crystallographic data for these compounds. These data can be obtained free of charge from The Cambridge Crystallographic Data Centre via www.ccdc.cam.ac.uk/data_request/cif (accessed on 5 December 2024).

Crystal structures of hydroxyphosphonate **2a** and hydroxyphosphine oxide **2d**; view of the unit cell along the a-axis, b-axis and c-axis are shown in Figures S1–S7. The selected bond lengths (Å), bond angles (°) and torsion angles (°) of hydroxyphosphonate **2a** are listed in Tables S1–S3, while the data for hydroxyphosphine oxide are listed in Tables S4–S6 in the Supplementary Materials.

### 3.9. Bioactivity Experimental

#### 3.9.1. Cell Culturing

The tested concentrations of the compounds were investigated on two cell lines: U266 human myeloma and PANC-1 human pancreatic ductal adenocarcinoma, which were obtained from the European Collection of Authenticated Cell Cultures (ECACC, Salisbury, UK). The PANC-1 cell line is an adherent cell culture (87092802 ECACC), whereas U266 cells (85051003 ECACC) grow in suspension. PANC-1 cells were maintained in DMEM medium (Sigma Ltd., St. Louis, MO, USA), while U266 cells were cultured in RPMI 1640 (Sigma Ltd., St. Louis, MO, USA). Both media were supplemented with fetal bovine serum (10%, Invitrogen Corporation, New York, NY, USA), L-glutamine (1%, Invitrogen Corporation, New York, NY, USA), and penicillin/streptomycin (1%, Invitrogen Corporation, New York, NY, USA).

#### 3.9.2. Cell Viability Assays

The tested compounds were dissolved in dimethyl sulfoxide (DMSO; AppliChem GmbH, Darmstadt, Germany) with a stock concentration of 10^−1^ M, keeping the DMSO concentration below 1% (*v*/*v*). Stock solutions were stored at −80 °C, with fresh solutions prepared for each experiment. Four compounds (**1f**, **2d**, **2e**, and **2f**) had poor solubility in DMSO; therefore, these were excluded from the cell viability studies. The viability of the PANC-1 cells was measured by the non-invasive xCELLigence system (ACEA Biosciences, San Diego, CA, USA), while to describe the viability of the U266 cells, the CellTiter-Glo Luminescent Cell Viability Assay (Promega, Madison, WI, USA) was applied. Both tests were conducted as previously described in our paper [58]. The cells were treated with the tested compounds at 1, 10, and 100 μM concentrations, as well as appropriate controls (medium and DMSO). All experiments were conducted in triplicate, with results normalized to the untreated medium control and presented as mean ± SD.

## 4. Conclusions

Robust synthetic methods were developed for the preparation of the series of α-hydroxy-alkylphosphonates and α-hydroxy-alkylphosphine oxides by different variations for the Pudovik reaction of acetaldehyde and acetone with different >P(O)H reagents including procedures, like the triethylamine-catalyzed liquid phase accomplishment, the solid phase addition on the surface of Al_2_O_3_/KF, and a Na_2_CO_3_-promoted MW synthesis. Single-crystal X-ray analyses revealed dimeric or linear chain structures in the solid phase. Four α-hydroxy-alkylphosphonates and a related phosphine oxide were converted to the corresponding acylated derivatives. One methylsulfonated species was also prepared that was a suitable reagent in the Michaelis–Arbuzov reaction with triethyl phosphite and ethyl diphenylphosphinite to provide bisphosphonic and phosphine oxide—phosphonate derivatives. The α-hydroxyphosphonates and α-hydroxyphosphine oxides synthesized exhibited limited cytotoxic effects on U266 cell lines with modest reductions in viability at a higher concentration.

## Figures and Tables

**Table 1:** Acylation of α-hydroxy-alkylphosphonate derivatives (**1b**–**d**, **2b**, and **2c**).

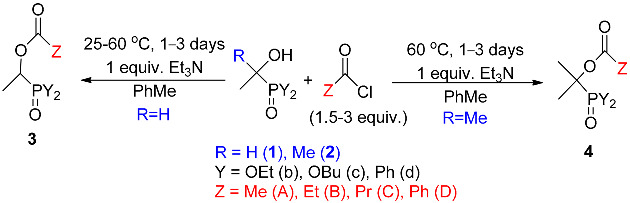									
Entry	R	Y	Starting Material	Z	ZC(O)Cl [Equiv.]	t [day]	T [°C]	Yield [%]	Product
1	H	OEt	**1b**	Me	3	1	25	65	**3Ab**
2	H	OEt	**1b**	Et	1.5	3	25	73	**3Bb**
3	H	OEt	**1b**	Pr	1.5	3	25	67	**3Cb**
4	H	OEt	**1b**	Ph	1.5	3	25	68	**3Db**
5	H	OBu	**1c**	Me	3	2	25	65	**3Ac**
6	H	Ph	**1d**	Me	3	3	60 ^1^	85	**3Ad**
7	Me	OEt	**2b**	Me	3	1	60 ^1^	62	**4Ab**
8	Me	OEt	**2b**	Et	1.5	3	60	62	**4Bb**
9	Me	OEt	**2b**	Pr	1.5	3	60	88	**4Cb**
10	Me	OBu	**2c**	Me	3	3	60 ^1^	61	**4Ac**

^1^ In a sealed tube.

**Table 2:** Synthesis of tetraethyl ethylidenebisphosphonate (**6a**) and diethyl α-(diphenylphosphinoyl)-ethylphosphonate (**6b**).

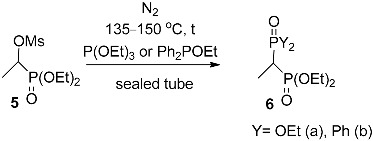							
Entry	P Reagent	P reagent [Equiv.]	t [day]	T [°C]	Conversion ^1^ [%]	Yield [%]	Product
1	P(OEt)_3_	5	2	135	25	-	**6a**
2		9	3	150	50	35	
3		9	3	150	62 ^2^	-	
4		9	6	150	66 (70 ^2^)	50 ^2^	
5	Ph_2_POEt	5	1	135	40	36	**6b**
6		5	2	150	70 ^2^	-	
7		5	3	150	100	75	

^1^ On the basis of the ^31^P NMR relative intensities. ^2^ In the presence of 10% of NiCl_2_.

**Table 3:** Cell viability of U266 myeloma and PANC-1 pancreatic adenocarcinoma cell lines following long-term treatment (72 h) with hydroxyphosphonate derivatives at 1, 10, or 100 μM. The data are expressed as mean ± standard deviation (SD), with a sample size of *n* = 3. Statistical analysis was performed using a one-way ANOVA followed by Fisher’s LSD post hoc test. Significance levels are denoted as follows: x: *p* < 0.05.

	PANC-1			U266		
Compound	1 μM	10 μM	100 μM	1 μM	10 μM	100 μM
**1b**	0.95 ± 0.04	1.08 ± 0.01	1.02 ± 0.03	0.88 ± 0.12	0.90 ± 0.03	0.85 ± 0.01
**1c**	0.97 ± 0.03	1.02 ± 0.04	0.94 ± 0.05	1.23 ± 0.26 ^x^	0.94 ± 0.03	0.91 ± 0.02
**1d**	1.07 ± 0.09	1.07 ± 0.01	1.02 ± 0.01	1.09 ± 0.09 ^x^	0.97 ± 0.03	0.85 ± 0.02
**1e**	1.09 ± 0.11 ^x^	1.07 ± 0.03	1.01 ± 0.02	1.14 ± 0.01 ^x^	1.00 ± 0.02	0.85 ± 0.04
**2a**	1.04 ± 0.06	1.02 ± 0.04	1.02 ± 0.06	0.95 ± 0.07	1.00 ± 0.05	0.84 ± 0.08
**2b**	0.94 ± 0.08	1.07 ± 0.02	0.99 ± 0.06	0.91 ± 0.04	1.01 ± 0.01	0.77 ± 0.06
**2c**	0.96 ± 0.10	1.11 ± 0.04	1.00 ± 0.04	1.01 ± 0.02	0.99 ± 0.05	0.95 ± 0.07

**Table 4:** Details for X-ray data collection and structure refinement for hydroxyphosphonate **2a** and hydroxyphosphine oxide **2d**.

	2a	2d
Empirical formula	C_5_H_13_O_4_P	C_15_H_17_O_2_P
Formula mass	168.12	348.39
T [K]	123(2)	123(2)
Crystal size [mm]	0.35 × 0.30 × 0.20	0.40 × 0.15 × 0.10
Crystal description	colorless block	colorless block
Crystal system	orthorhombic	monoclinic
Space group	*Pbca*	*P*21/*c*
a [Ǻ]	7.8977(2)	11.0791(3)
b [Ǻ]	11.5547(2)	10.3343(3)
c [Ǻ]	17.7550(4)	11.5536(3)
α [°]	90.0	90.0
β [°]	90.0	96.055(2)
γ [°]	90.0	90.0
V [Ǻ^3^]	1620.24(6)	1315.45(6)
Z	8	4
ρ_calcd._ [g cm^−3^]	1.378	1.314
μ [mm^−1^]	0.299	0.200
*F*(000)	720	552
Θ range [°]	3.33–25.24	1.85–25.24
Index ranges	−11 ≤ *h* ≤ 11	−15 ≤ *h* ≤ 15
	−16 ≤ *k* ≤ 16	−14 ≤ *k* ≤ 14
	−25 ≤ *l* ≤ 25	−16 ≤ *l* ≤ 16
Reflns. collected	30,439	25,660
Reflns. obsd.	2228	3327
Reflns. unique	2472(R_int_ = 0.0275)	4016(R_int_ = 0.0380)
*R*_1_, *wR*_2_ (2σ data)	0.0250, 0.0697	0.0357, 0.0913
*R*_1_, *wR*_2_ (all data)	0.0289, 0.0721	0.0461, 0.0993
GOOF on *F*^2^	1.047	1.054
Peak/hole [e Ǻ^−3^]	0.427/−0.272	0.377/−0.334

## Data Availability

The data presented in this study are available on request from the corresponding authors.

## Supplementary Materials

The following supporting information can be downloaded at: https://www.mdpi.com/article/10.3390/molecules30020428/s1, X-ray data for compounds **2a** and **2d**; ^31^P, ^13^C and ^1^H NMR spectra of all of the compounds prepared.
