# Supplementary material for: The Synthesis, Crystal Structure, Modification, and Cytotoxic Activity of α-Hydroxy-Alkylphosphonates
Source: Molecules. 2025 Jan 20;30(2):428. doi: 10.3390/molecules30020428 (PMC11767484; doi:10.3390/molecules30020428)

Supplementary Materials

The Synthesis, Crystal Structure, Modification, and Cytotoxic Activity of α-Hydroxy-Alkylphosphonates

Zsuzsanna Szalai,[1] Anna Sára Kis,[1] Angéla Takács,[2] László Kőhidai,[2] Konstantin Karaghiosoff,[3] Mátyás Czugler,[1] László Drahos,[4] György Keglevich[1]*

[1] *Department of Organic Chemistry and Technology, Faculty of Chemical Technology and Biotechnology, Budapest University of Technology and Economics, 1111 Budapest, Műegyetem rkp. 3., Hungary; szalai.zsuzsanna@edu.bme.hu; kisannasari@gmail.com; czuglrem-pu@gmail.hu; keglevich.gyorgy@vbk.bme.hu**

[2] *Department of Genetics, Cell-and Immunobiology, Semmelweis University, Nagyvárad tér 4, 1089 Budapest, Hungary; takacs.angela@semmelweis.hu; kohidai.laszlo@semmelweis.hu*

[3] *Department Chemie, Ludwig-Maximilians-Universität München, Butenandtstr. 5-13, D-81377 München, Germany, klk@cup.uni-muenchen.de*

[4] *MS Proteomics Research Group, Research Centre for Natural Sciences, 1117 Budapest, Hungary; drahos.laszlo@ttk.hu*

Correspondence: keglevich.gyorgy@vbk.bme.hu; Tel.: +36-1-463-1111 (ext. 5883)

Table of Contents

1. Principal X-ray geometry data for hydroxyphosphonate **2a** and hydroxyphosphine oxide **2d** obtained from the X-ray measurements S2

2. Spectra for the compounds **1b-f**, **2a-f**, **3Ab-Db**, **3Ac**, **3Ad**, **4Ab-Cb,** **4Ac**, **5**, **6a** and **6b** synthesized S11

1. Principal X-ray geometry data for hydroxyphosphonate 2a and hydroxyphosphine oxide 2d obtained from the X-ray measurements

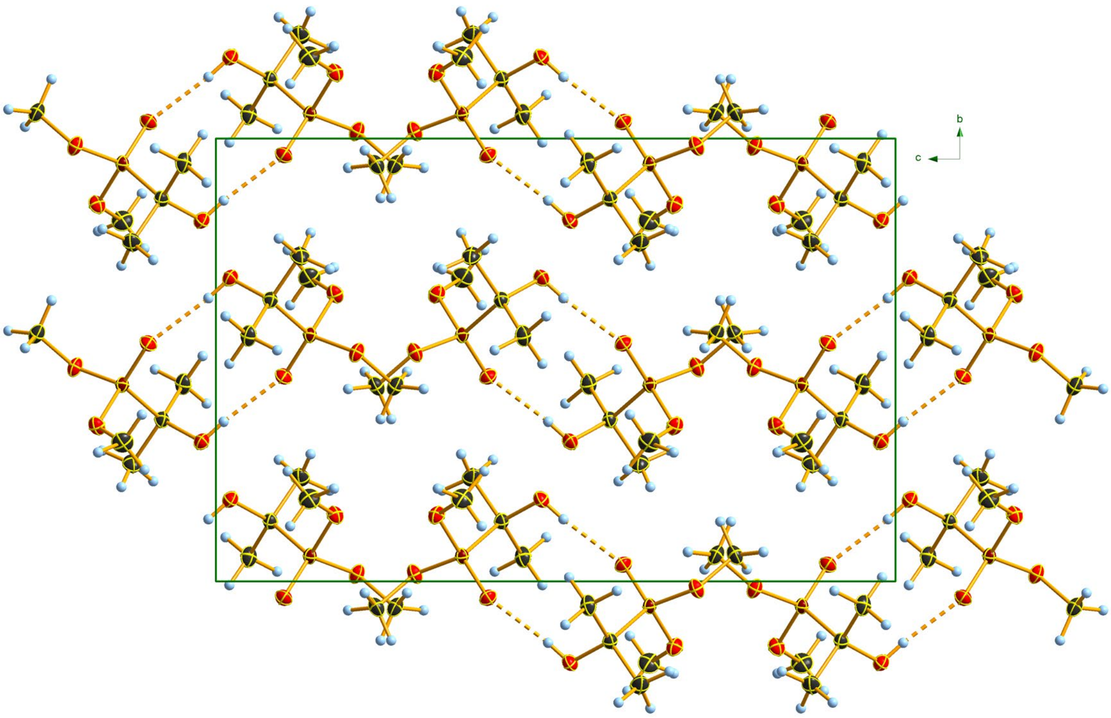

Figure S1. Crystal structure of hydroxyphosphonate **2a**; view of the unit cell along the *a*-axis. DIAMOND [50] representation; thermal ellipsoids are drawn at 50% probability level.

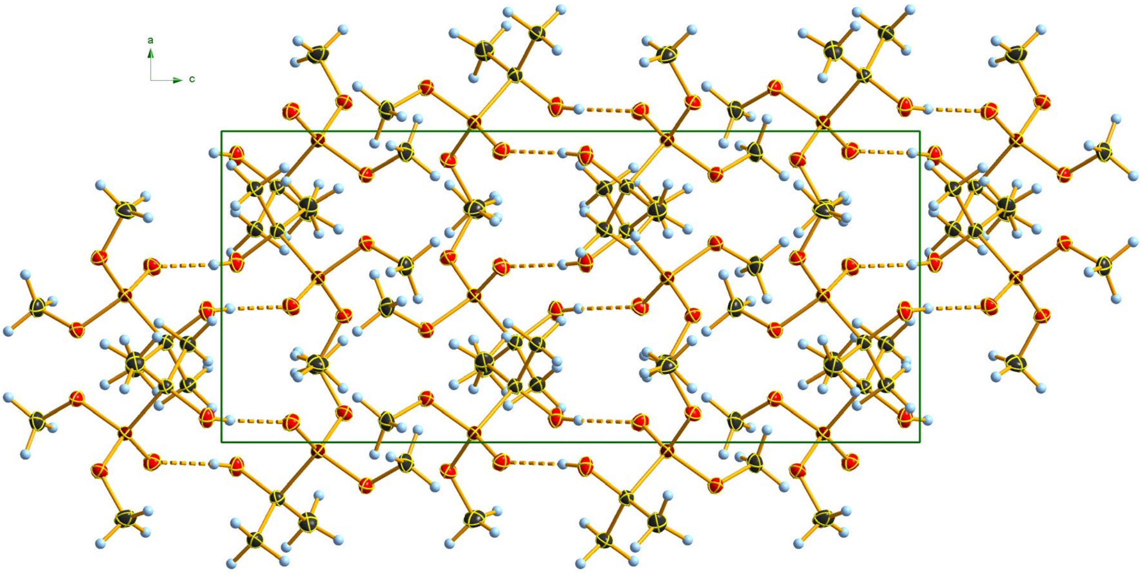

Figure S2. Crystal structure of hydroxyphosphonate **2a**; view of the unit cell along the *b*-axis. DIAMOND [50] representation; thermal ellipsoids are drawn at 50% probability level.

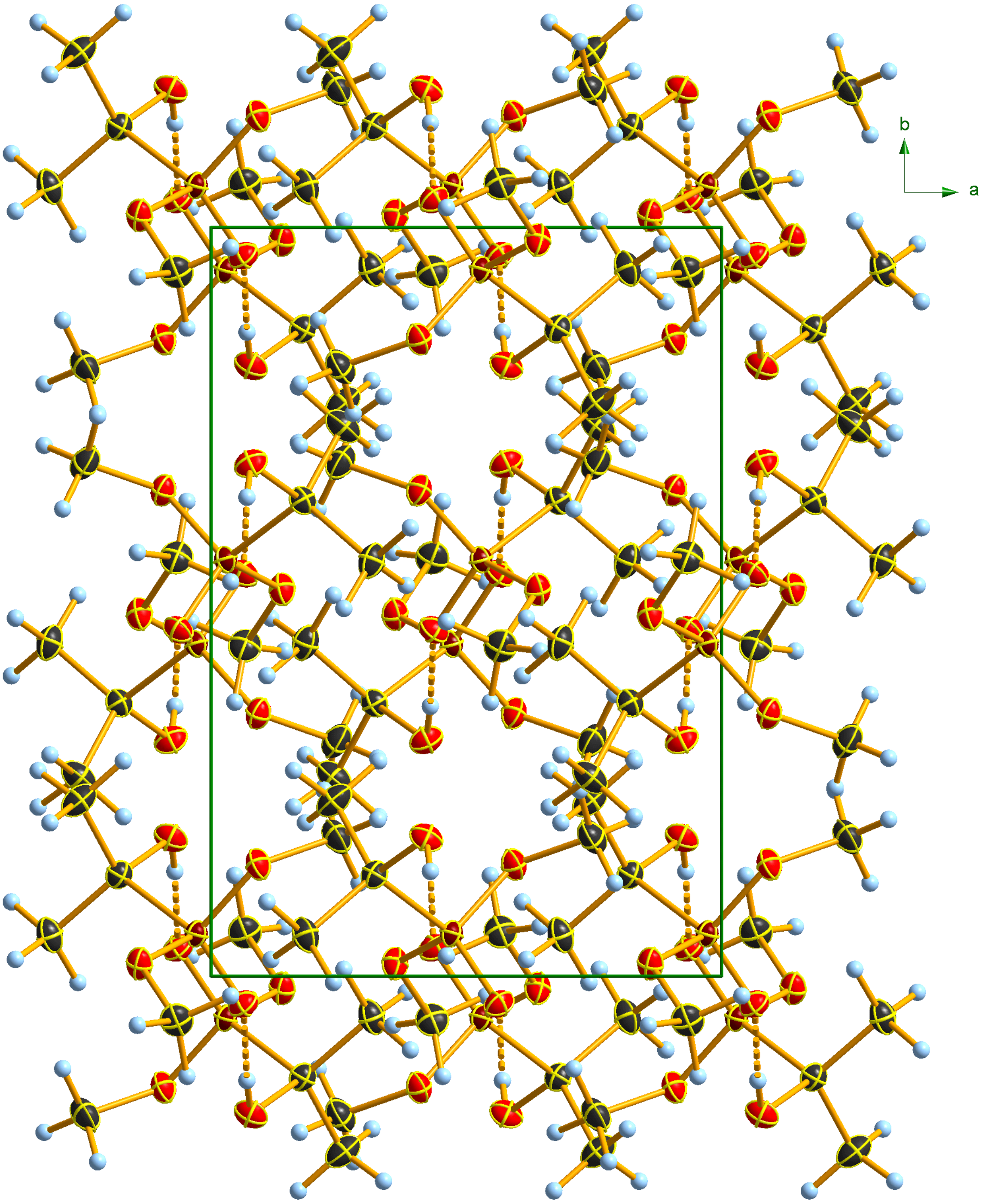

Figure S3. Crystal structure of hydroxyphosphonate **2a**; view of the unit cell along the *c*-axis. DIAMOND [50] representation; thermal ellipsoids are drawn at 50% probability level.

Table S1. Selected bond lengths (Å) of hydroxyphosphonate **2a**.

P1 – O2	1.476(1)	O3 – C4	1.448(1)
P1 – O3	1.576(1)	O4 – C5	1.446(1)
P1 – O4	1.581(1)	C1 – C3	1.520(1)
P1 – C1	1.824(1)	C1 – C2	1.526(1)
O1 – C1	1.432(1)		

Table S2. Selected bond angles (°) of hydroxyphosphonate **2a**.

O2 – P1 – O3	115.0(1)	C5 – O4 – P1	121.0(1)
O2 – P1 – O4	112.9(1)	O1 – C1 – C3	107.5(1)
O3 – P1 – O4	102.2(1)	O1 – C1 – C2	111.4(1)
O2 – P1 – C1	115.3(1)	C3 – C1 – C2	111.3(1)
O3 – P1 – C1	103.0(1)	O1 – C1 – P1	104.4(1)
O4 – P1 – C1	107.1(1)	C3 – C1 – P1	110.7(1)
C4 – O3 – P1	119.6(1)	C2 – C1 – P1	111.3(1)

Table S3. Selected torsion angles (°) of hydroxyphosphonate **2a**.

O2 – P1 – O3 – C4	48.2(1)	O4 – P1 – C1 – O1	65.8(1)
O4 – P1 – O3 – C4	-74.6(1)	O2 – P1 – C1 – C3	-176.2(1)
C1 – P1 – O3 – C4	174.4(1)	O3 – P1 – C1 – C3	57.8(1)
O2 – P1 – O4 – C5	38.9(1)	O4 – P1 – C1 – C3	-49.6(1)
O3 – P1 – O4 – C5	163.1(1)	O2 – P1 – C1 – C2	59.6(1)
C1 – P1 – O4 – C5	-89.0(1)	O3 – P1 – C1 – C2	-66.5(1)
O2 – P1 – C1 – O1	-60.8(1)	O4 – P1 – C1 – C2	-173.9(1)
O3 – P1 – C1 – O1	173.2(1)		

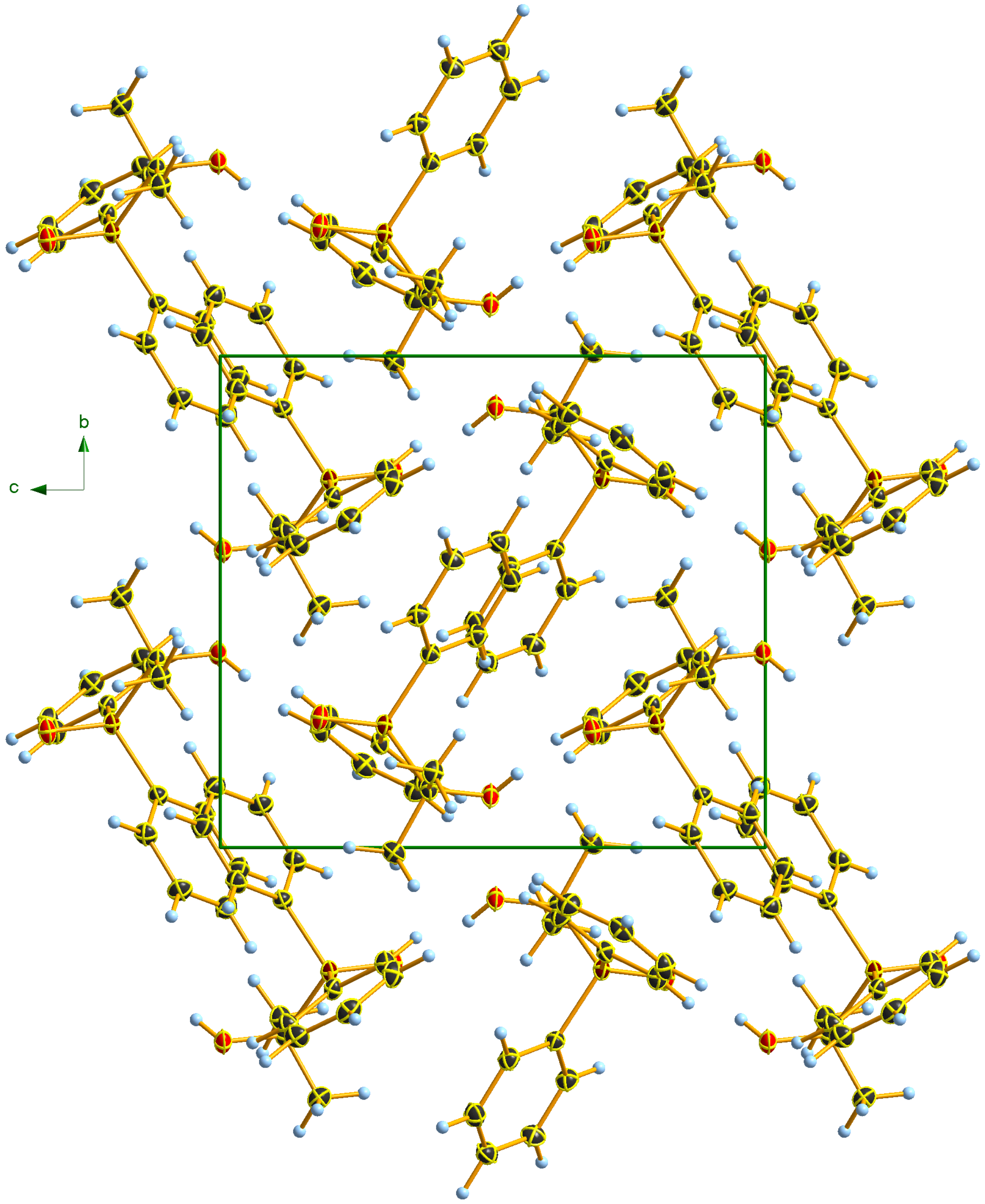

Figure S4. Crystal structure of hydroxyphosphine oxide **2d**; view of the unit cell along the *a*-axis. DIAMOND [50] representation; thermal ellipsoids are drawn at 50% probability level.

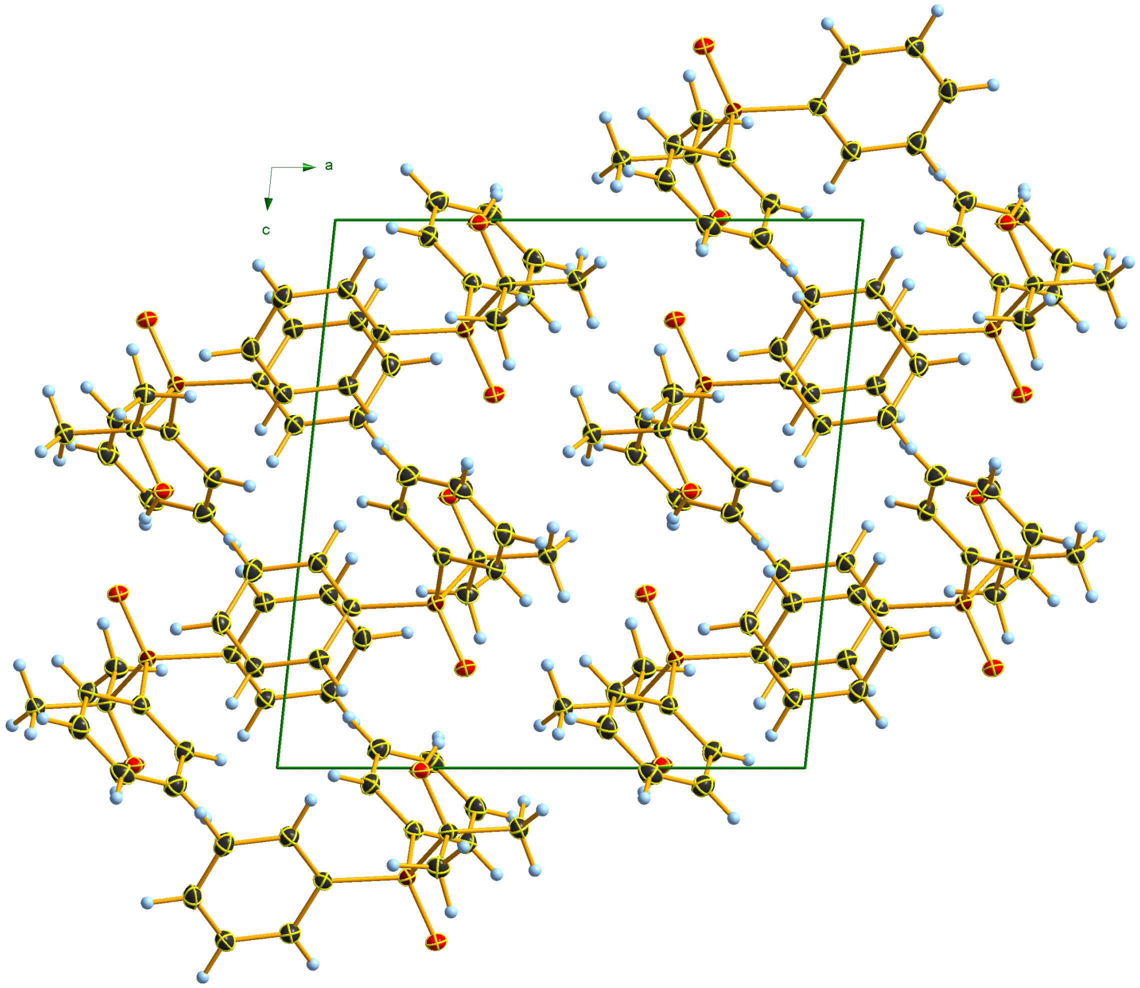

Figure S5. Crystal structure of hydroxyphosphine oxide **2d**; view of the unit cell along the *b*-axis. DIAMOND [50] representation; thermal ellipsoids are drawn at 50% probability level.

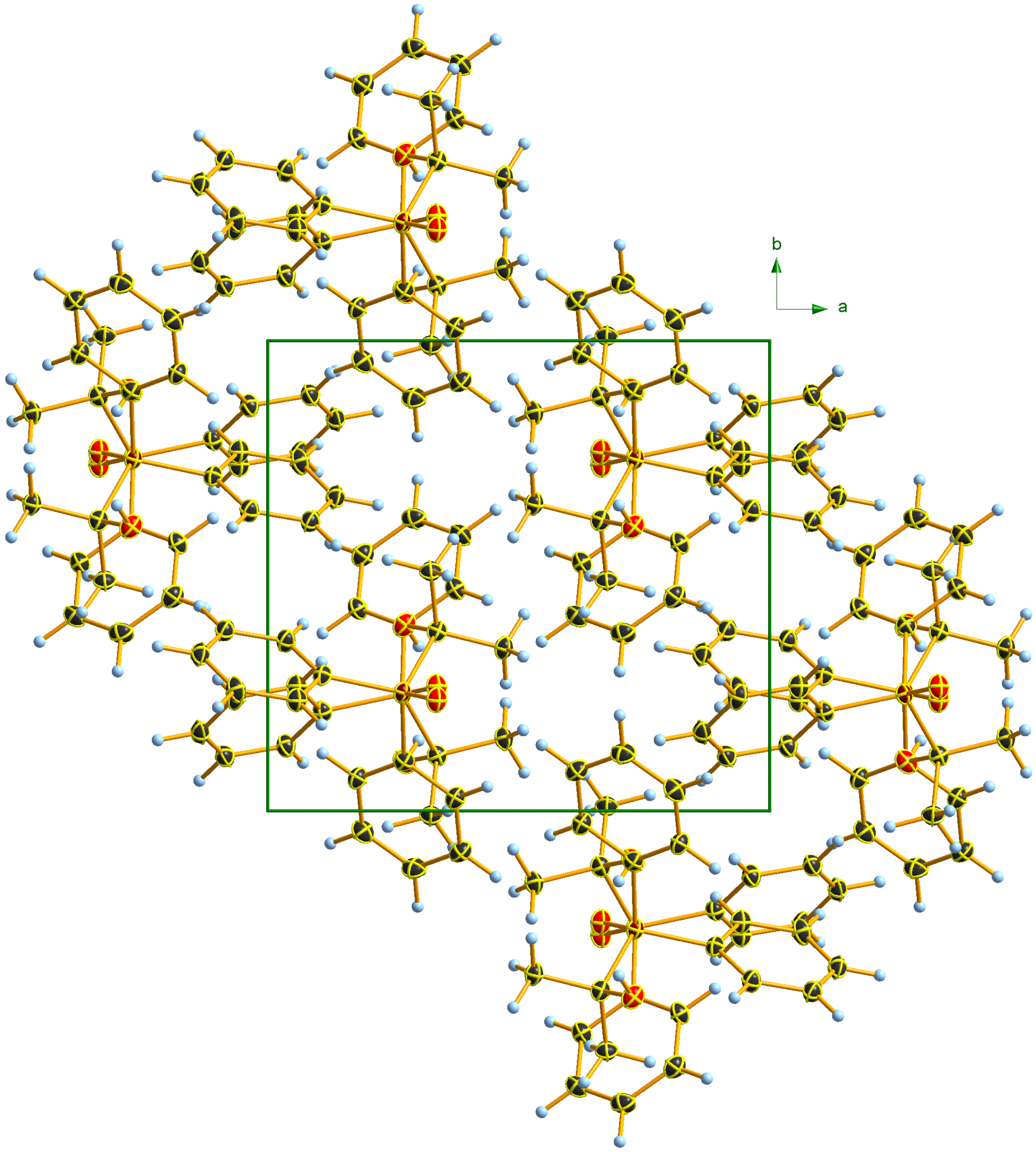

Figure S6. Crystal structure of hydroxyphosphine oxide **2d**; view of the unit cell along the *c*-axis. DIAMOND [50] representation; thermal ellipsoids are drawn at 50% probability level.

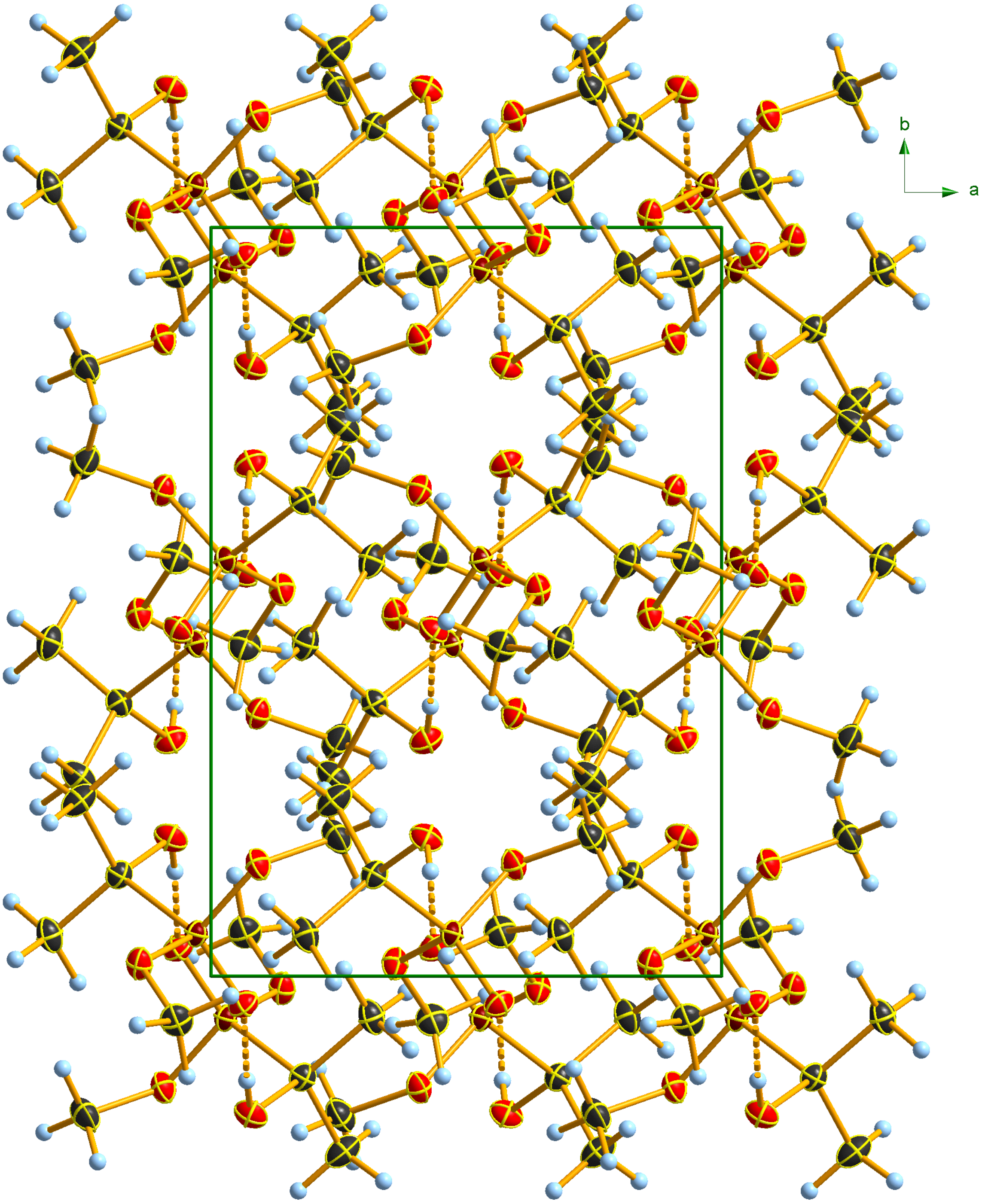

Figure S7. Crystal structure of hydroxyphosphine oxide **2d**; view of the unit cell along the *c*-axis. DIAMOND [50] representation; thermal ellipsoids are drawn at 50% probability level.

Table S4. Selected bond lengths (Å) of hydroxyphosphine oxide **2d**.

P1 – O1	1.499(1)	C14 – C15	1.389(2)
P1 – C10	1.803(1)	C14 – C13	1.389(2)
P1 – C4	1.808(1)	C11 – C12	1.391(2)
P1 – C1	1.858(1)	C9 – C8	1.389(2)
O2 – C1	1.425(1)	C12 – C13	1.392(2)
C1 – C2	1.526(2)	C8 – C7	1.387(2)
C1 – C3	1.528(2)	C6 – C5	1.393(2)
C4 – C9	1.398(2)	C10 – C11	1.395(2)
C4 – C5	1.400(2)	C10 – C15	1.398(2)
C6 – C7	1.389(2)		

Table S5. Selected bond angles (°) of hydroxyphosphine oxide **2d**.

O1 – P1 – C10	111.6(1)	C14 – C15 – C10	120.4(1)
O1 – P1 – C4	112.5(1)	C12 – C11 – C10	120.1(1)
C10 – P1 – C4	106.0(1)	C8 – C9 – C4	120.6(1)
O1 – P1 – C1	110.4(1)	C11 – C12 – C13	120.0(1)
C10 – P1 – C1	106.8(1)	C14 – C13 – C12	120.3(1)
C4 – P1 – C1	109.4(1)	C7 – C8 – C9	120.1(1)
O2 – C1 – C2	111.6(1)	C8 – C7 – C6	120.0(1)
O2 – C1 – C3	107.4(1)	C6 – C5 – C4	120.2(1)
C2 – C1 – C3	111.6(1)	C5 – C4 – P1	125.1(1)
O2 – C1 – P1	110.0(1)	C7 – C6 – C5	120.1(1)
C2 – C1 – P1	107.9(1)	C11 – C10 – C15	119.4(1)
C3 – C1 – P1	108.3(1)	C11 – C10 – P1	123.6(1)
C9 – C4 – C5	119.0(1)	C15 – C10 – P1	117.0(1)
C9 – C4 – P1	115.9(1)	C15 – C14 – C13	119.8(1)

Table S6. Selected torsion angles (°) of hydroxyphosphine oxide **2d**.

O1 – P1 – C1 – O2	179.2(1)	C4 – P1 – C10 – C15	-152.5(1)
C10 – P1 – C1 – O2	57.8(1)	C1 – P1 – C10 – C15	91.0(1)
C4 – P1 – C1 – O2	-56.5(1)	C13 – C14 – C15 – C10	-0.1(2)
O1 – P1 – C1 – C2	57.3(1)	C11 – C10 – C15 – C14	0.4(2)
C10 – P1 – C1 – C2	-64.1(1)	P1 – C10 – C15 – C14	-179.8(1)
C4 – P1 – C1 – C2	-178.4(1)	C15 – C10 – C11 – C12	-0.5(2)
O1 – P1 – C1 – C3	-63.7(1)	P1 – C10 – C11 – C12	179.7(1)
C10 – P1 – C1 – C3	174.9(1)	C5 – C4 – C9 – C8	-0.6(2)
C4 – P1 – C1 – C3	60.6(1)	P1 – C4 – C9 – C8	179.8(1)
O1 – P1 – C4 – C9	-30.7(1)	C10 – C11 – C12 – C13	0.3(2)
C10 – P1 – C4 – C9	91.5(1)	C15 – C14 – C13 – C12	-0.2(2)
C1 – P1 – C4 – C9	-153.7(1)	C11 – C12 – C13 – C14	0.0(2)
O1 – P1 – C4 – C5	149.7(1)	C4 – C9 – C8 – C7	-0.4(2)
C10 – P1 – C4 – C5	-88.1(1)	C9 – C8 – C7 – C6	1.3(2)
C1 – P1 – C4 – C5	26.7(1)	C5 – C6 – C7 – C8	-1.1(2)
O1 – P1 – C10 – C11	150.1(1)	C7 – C6 – C5 – C4	0.1(2)
C4 – P1 – C10 – C11	27.3(1)	C9 – C4 – C5 – C6	0.8(2)
C1 – P1 – C10 – C11	-89.3(1)	P1 – C4 – C5 – C6	-179.7(1)
O1 – P1 – C10 – C15	-29.7(1)		

2. Spectra for the compounds 1b-f, 2a-f, 3Ab-Db, 3Ac, 3Ad, 4Ab-Cb, 4Ac, 5, 6a and 6b synthesized

^{31}P {^{1}H} NMR (122 MHz, $CDCl_3$) spectra for 1b

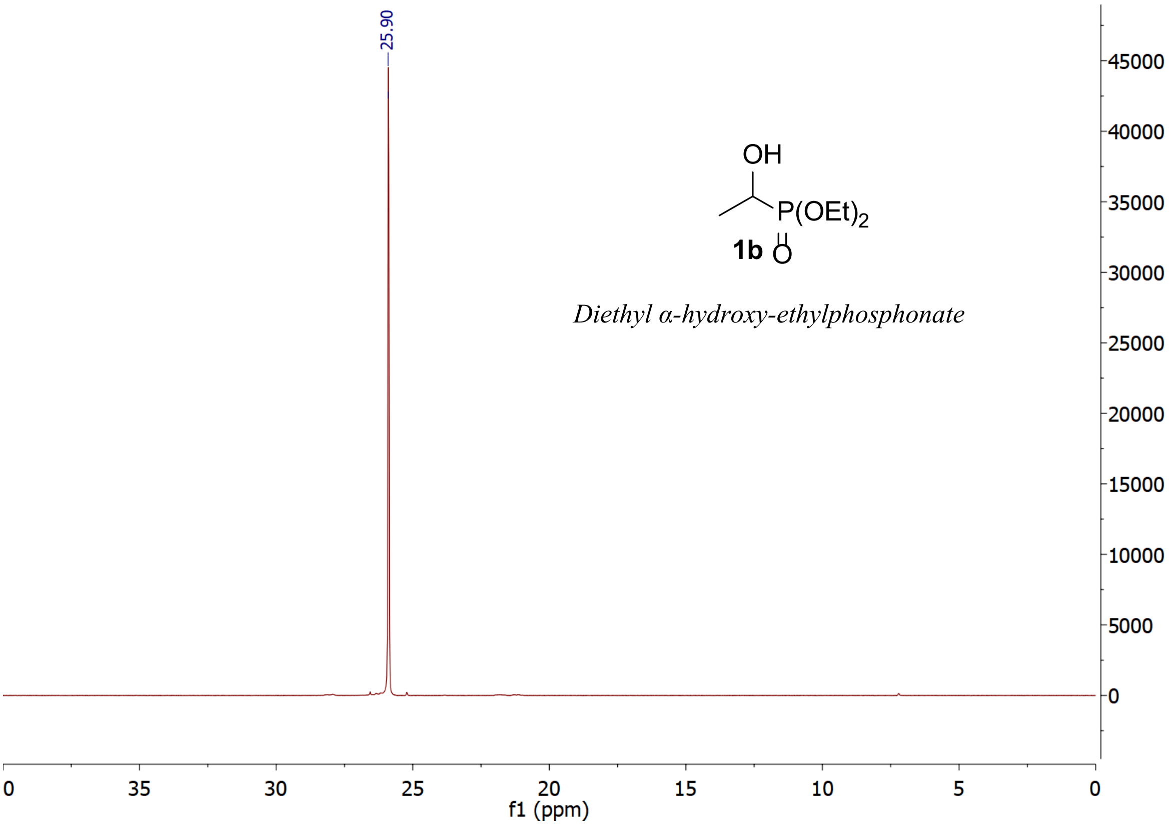

^{31}P {^{1}H} NMR (202 MHz, $CDCl_3$) spectra for 1c

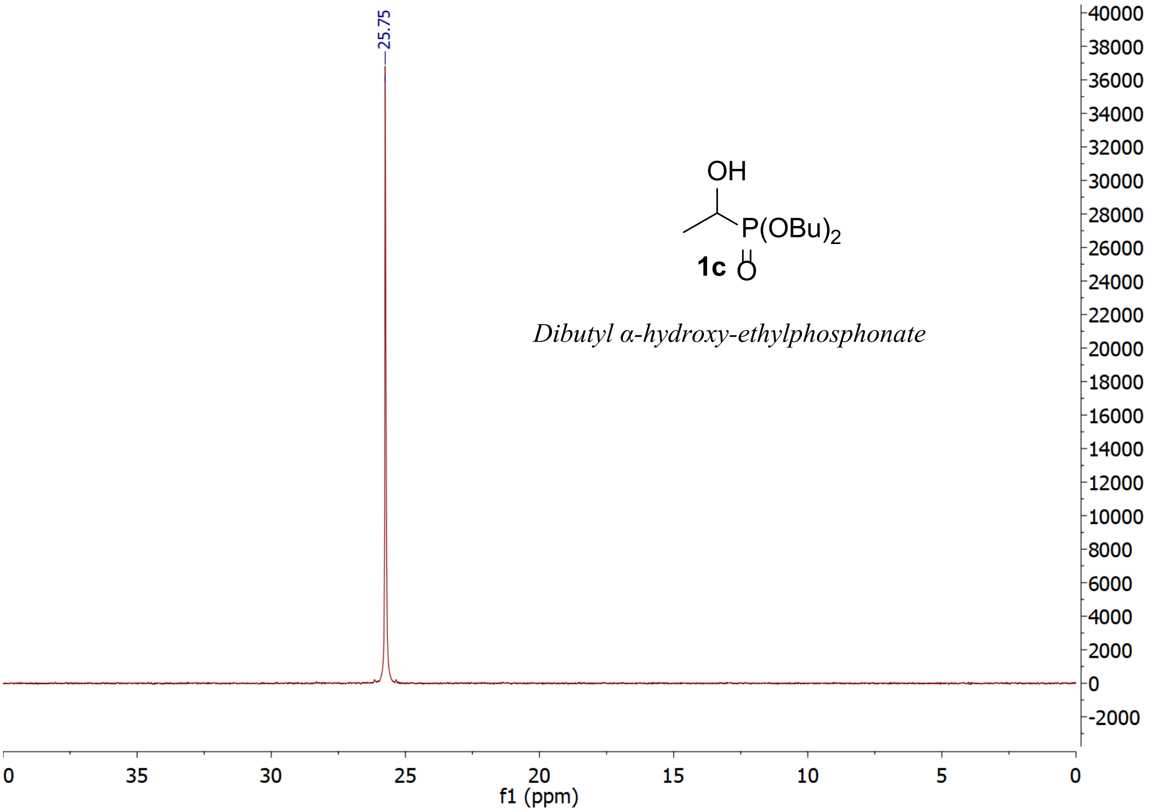

^{31}P {^{1}H} NMR (202 MHz, $CDCl_3$) spectra for 1d

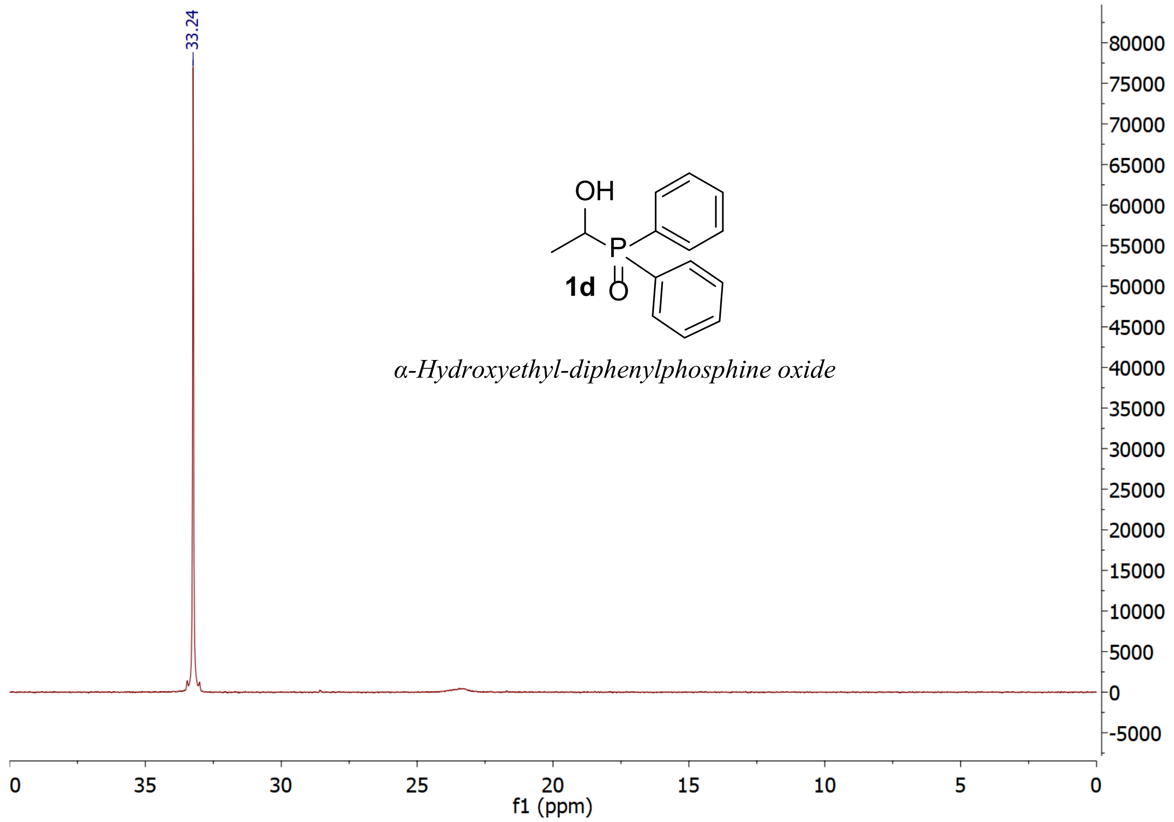

^{31}P {^{1}H} NMR (202 MHz, $CDCl_3$) spectra for 1e

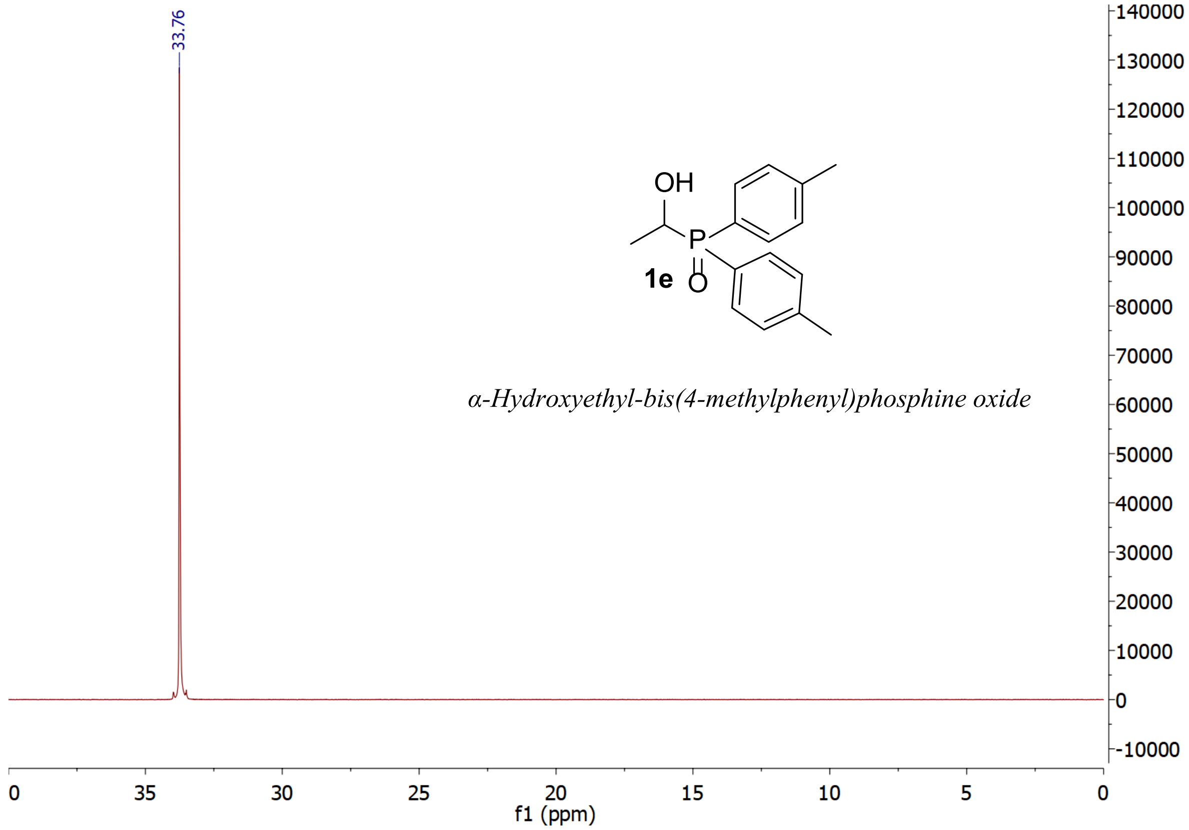

^{31}P {^{1}H} NMR (202 MHz, $CDCl_3$) spectra for 1f

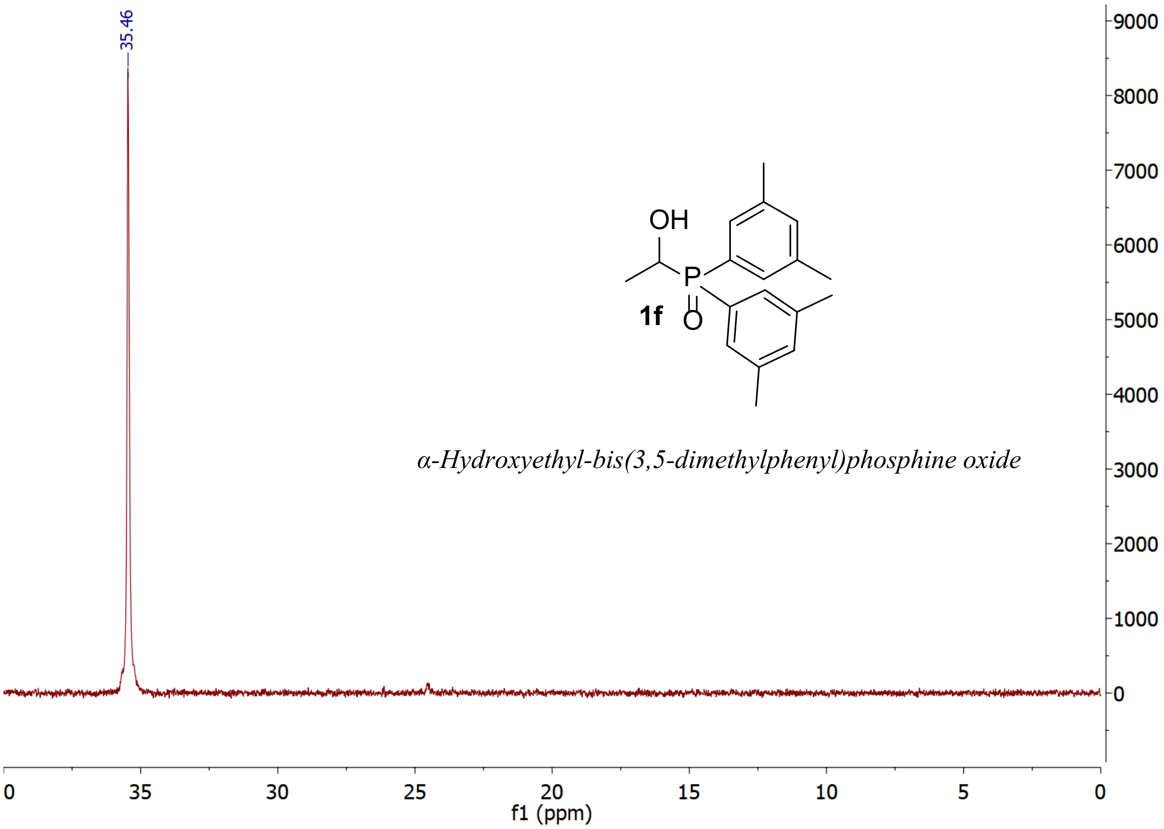

^{31}P {^{1}H} NMR (202 MHz, $CDCl_3$) spectra for 2a

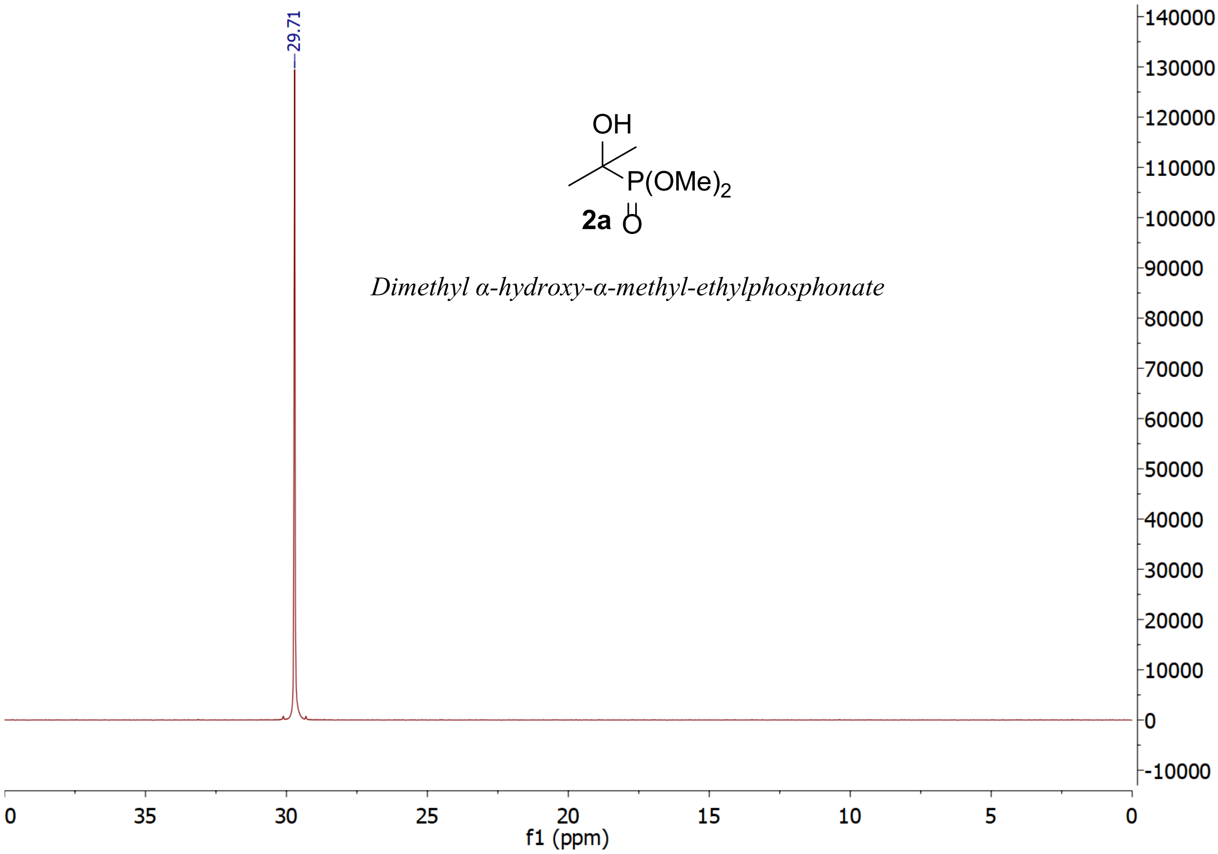

$^{31}P\ \{^1H\}$ NMR (202 MHz, $CDCl_3$) spectra for 2b

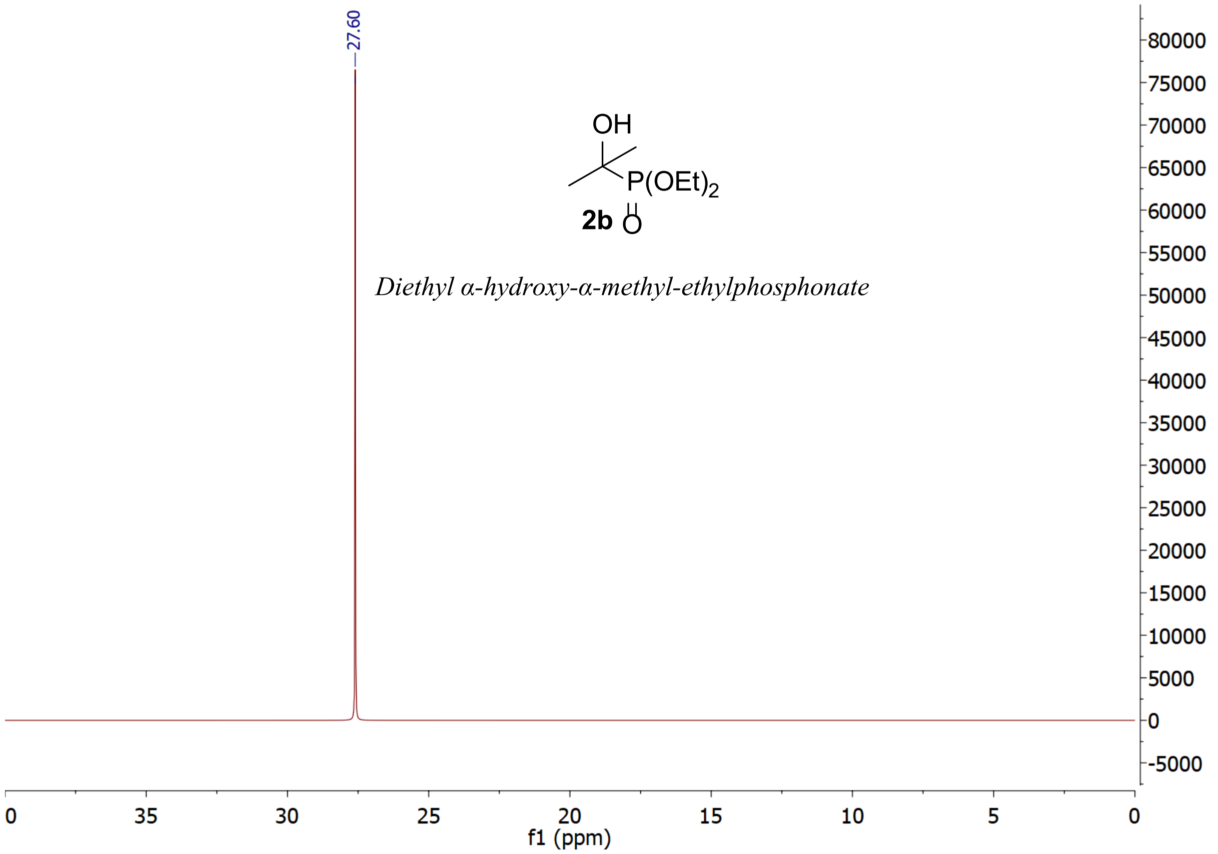

$^{31}P\ \{^1H\}$ NMR (202 MHz, $CDCl_3$) spectra for 2c

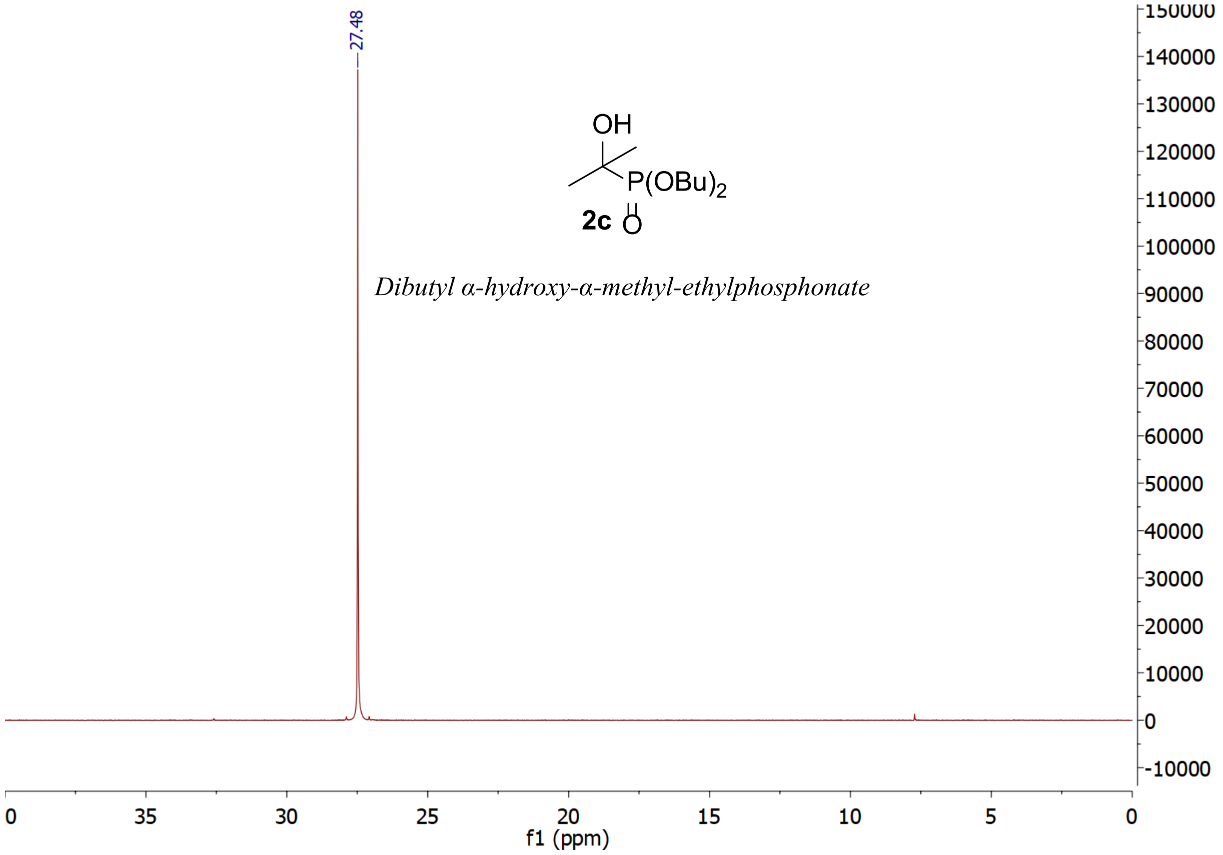

^{31}P {^{1}H} NMR (202 MHz, $CDCl_3$) spectra for 2d

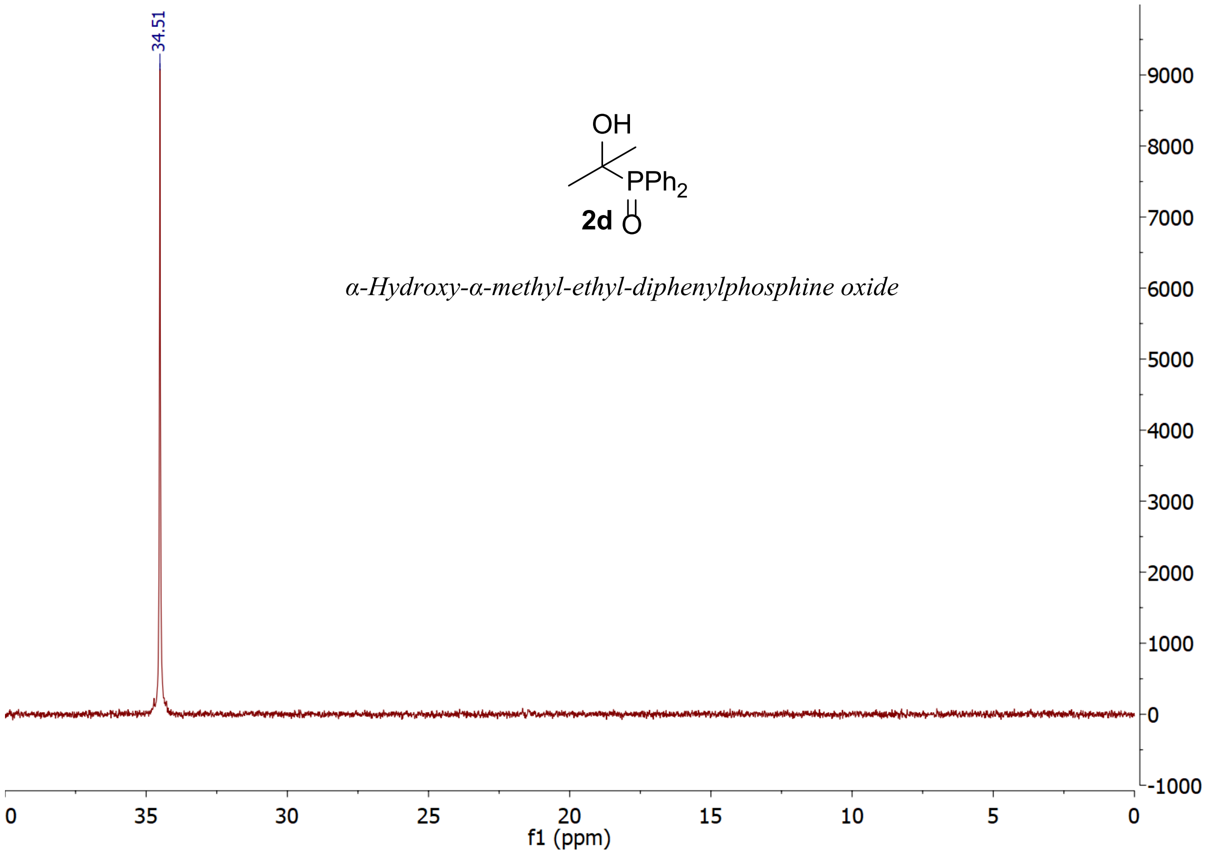

^{31}P {^{1}H} NMR (202 MHz, $CDCl_3$) spectra for 2e

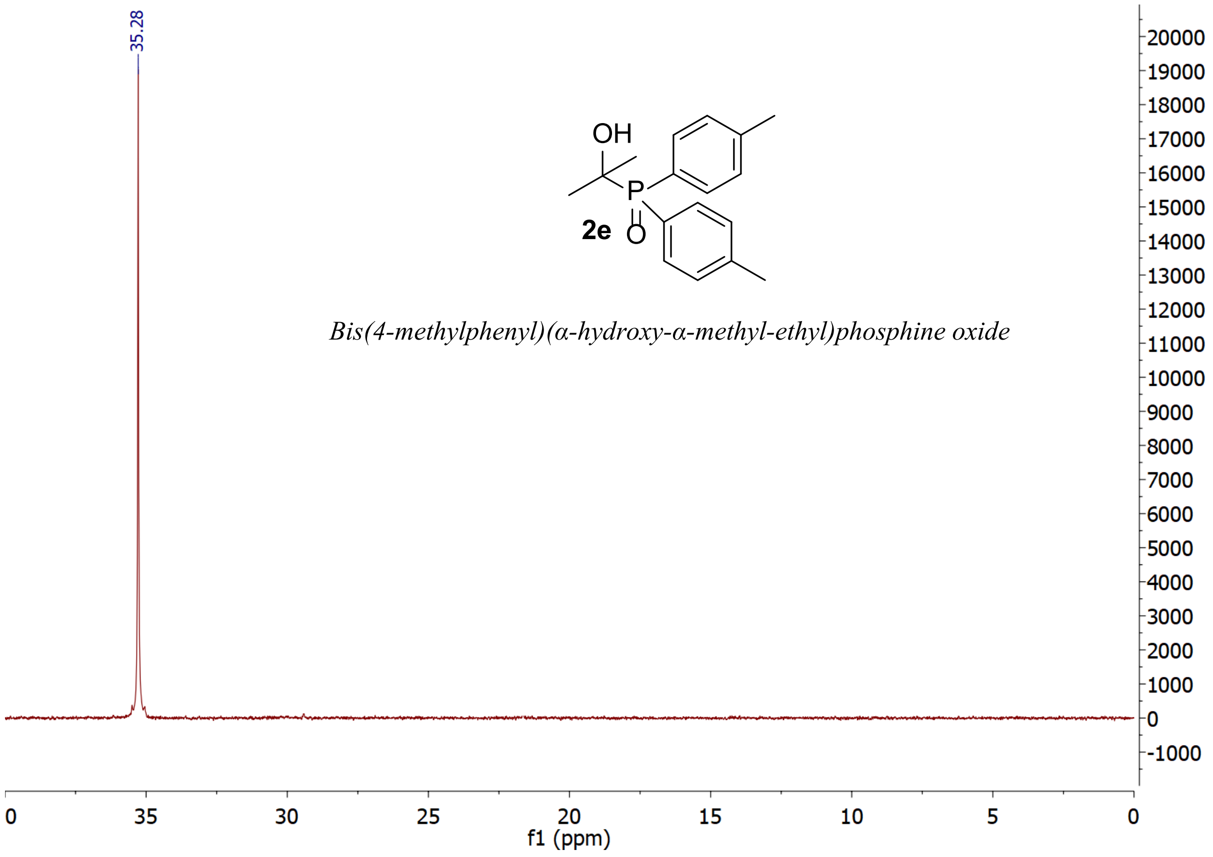

^{31}P {^1H} NMR (202 MHz, $CDCl_3$) spectra for 2f

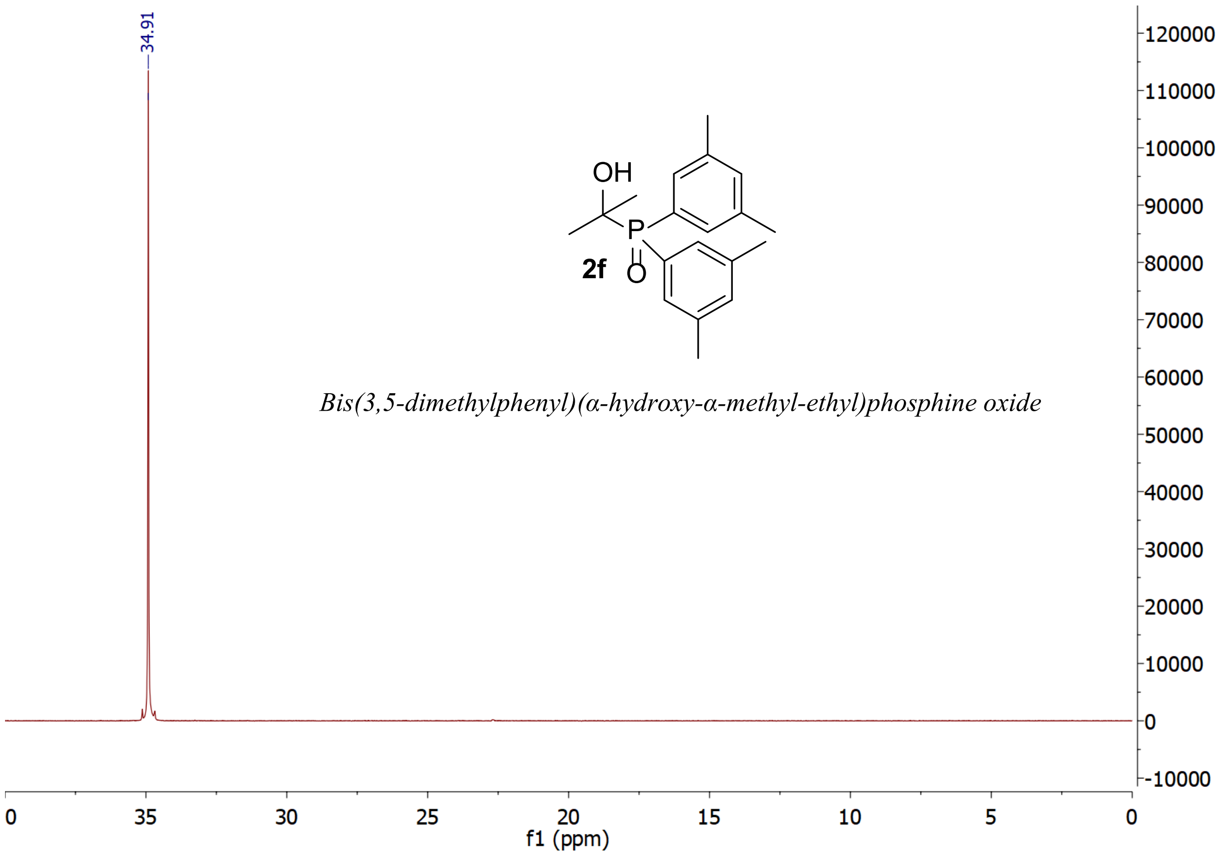

^{13}C {^1H} NMR (126 MHz, $CDCl_3$) spectra for 2f

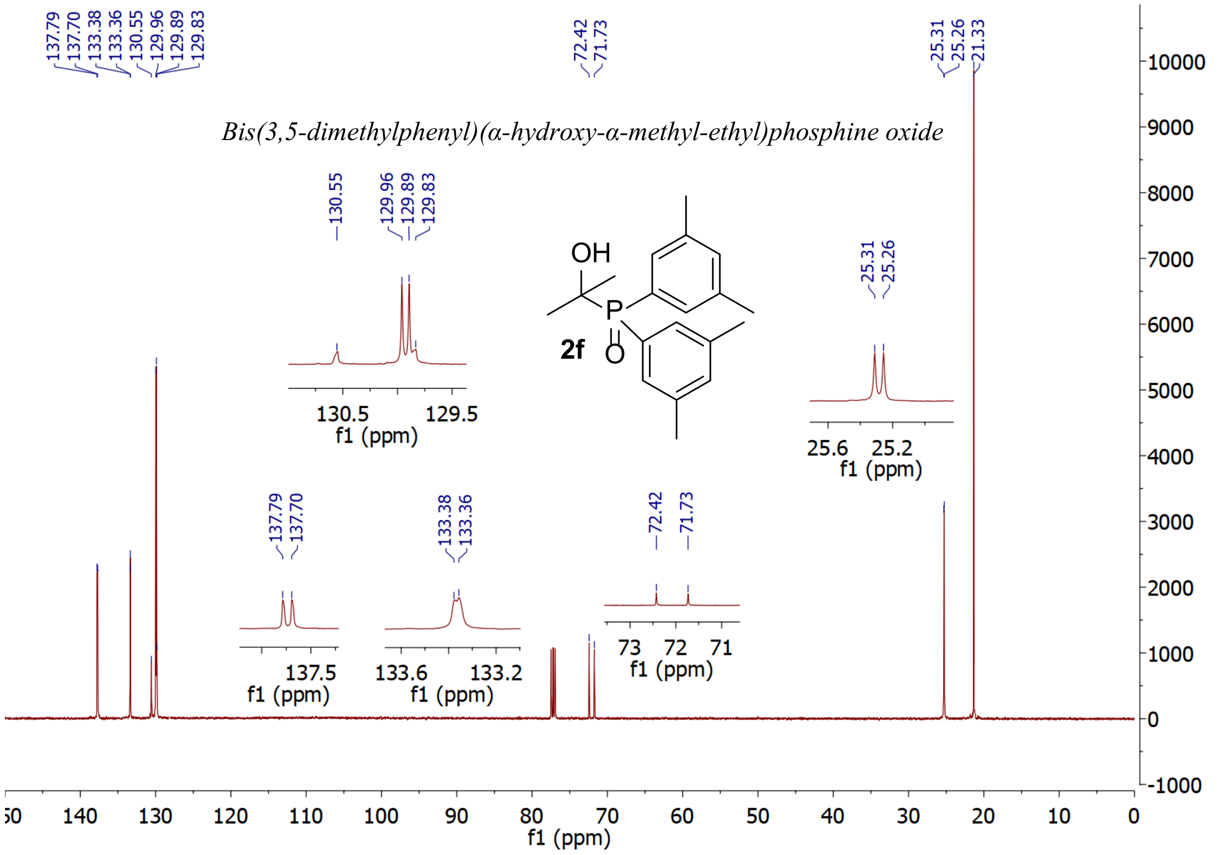

^{1}H NMR (500 MHz, $CDCl_3$) spectra for 2f

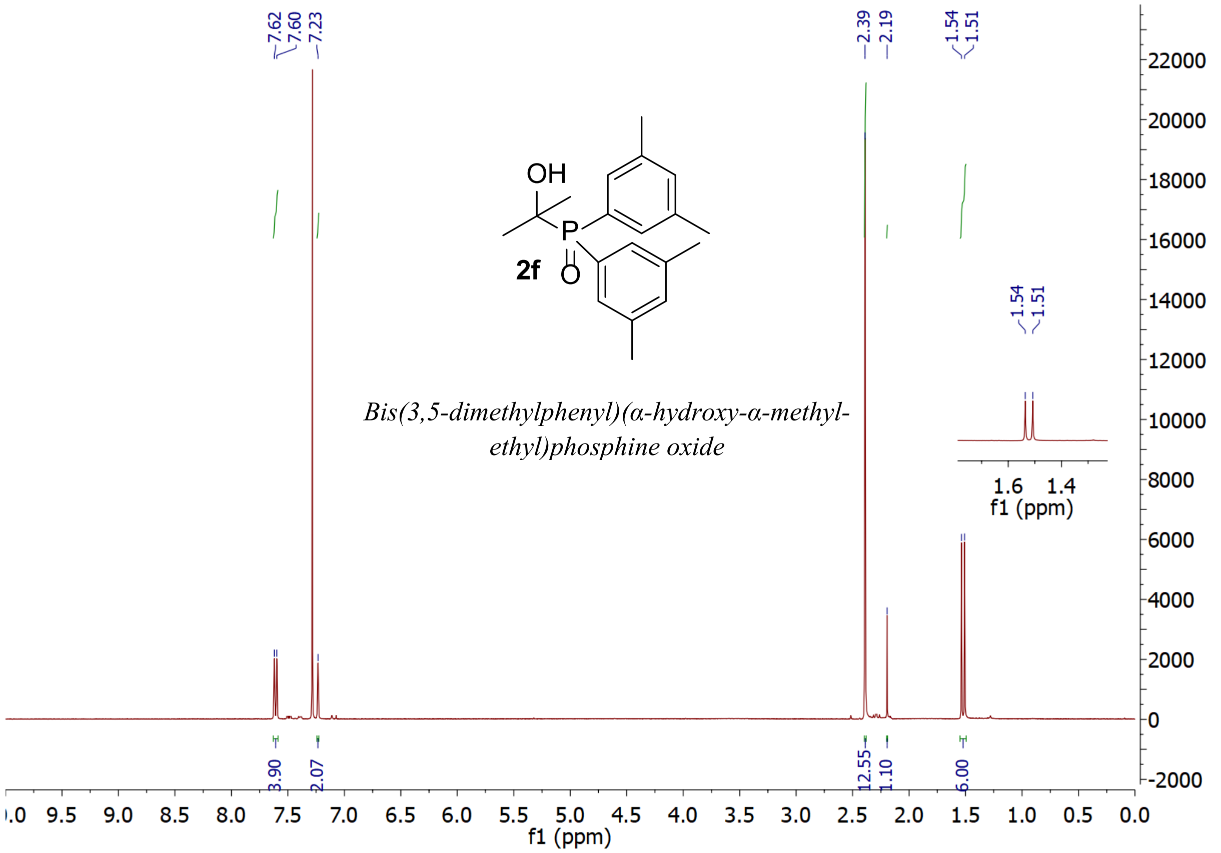

^{31}P {^{1}H} NMR (202 MHz, $CDCl_3$) spectra for 3Ab

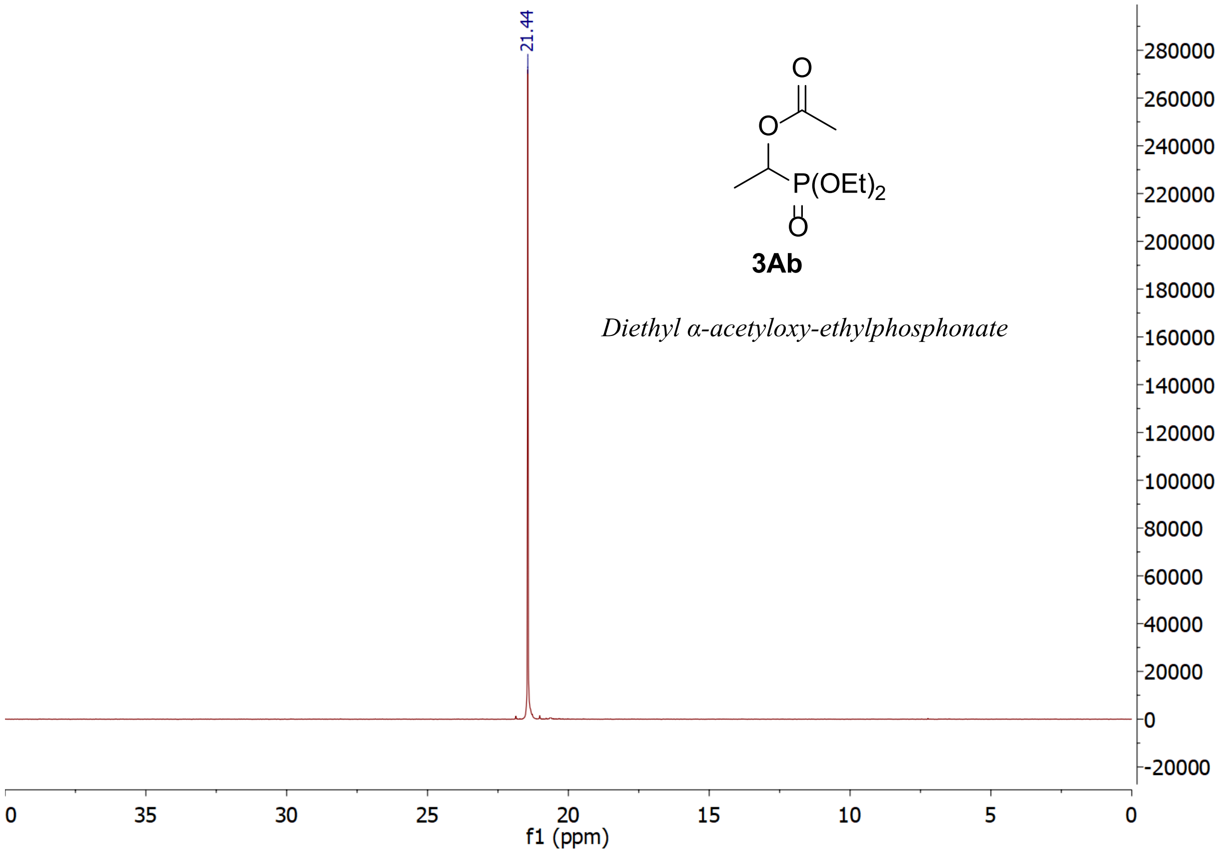

$^{31}P\{^1H\}$ NMR (202 MHz, $CDCl_3$) spectra for 3Bb

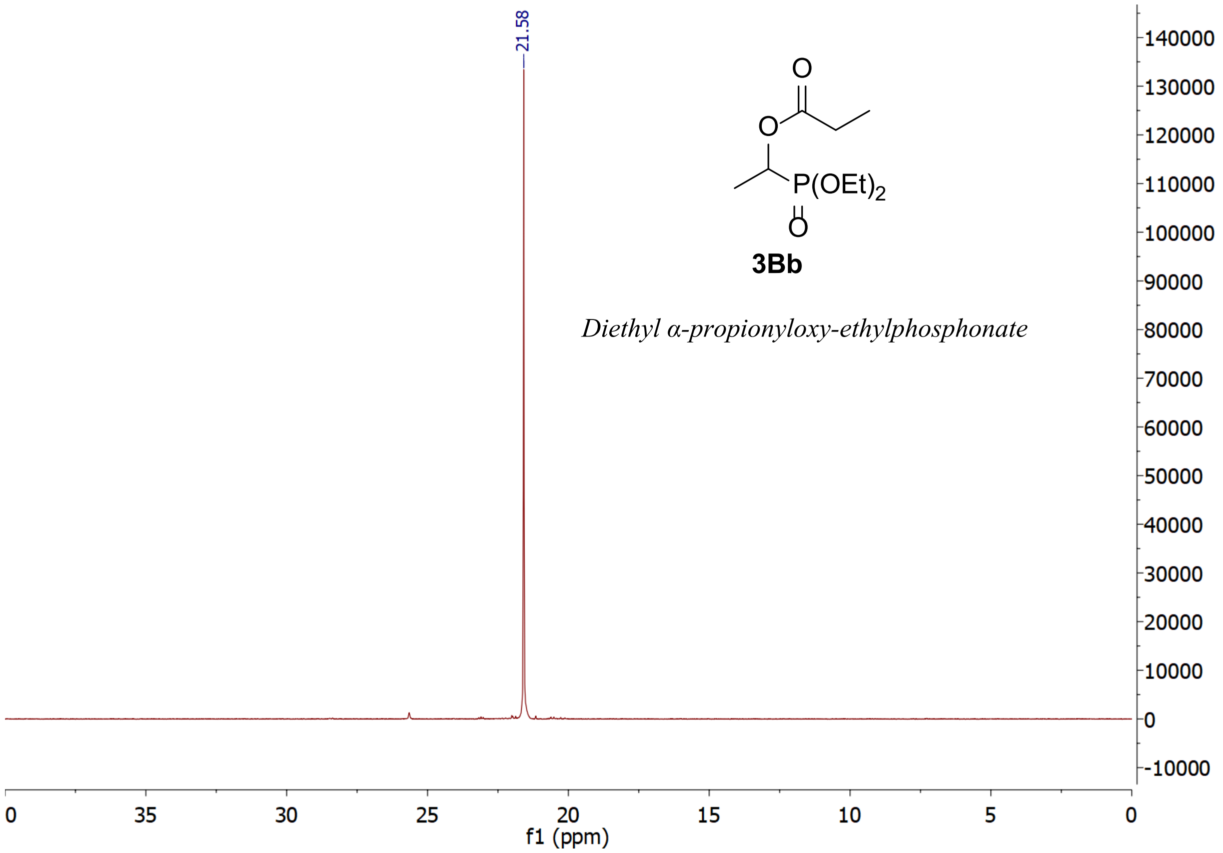

$^{13}C\{^1H\}$ NMR (126 MHz, $CDCl_3$) spectra for 3Bb

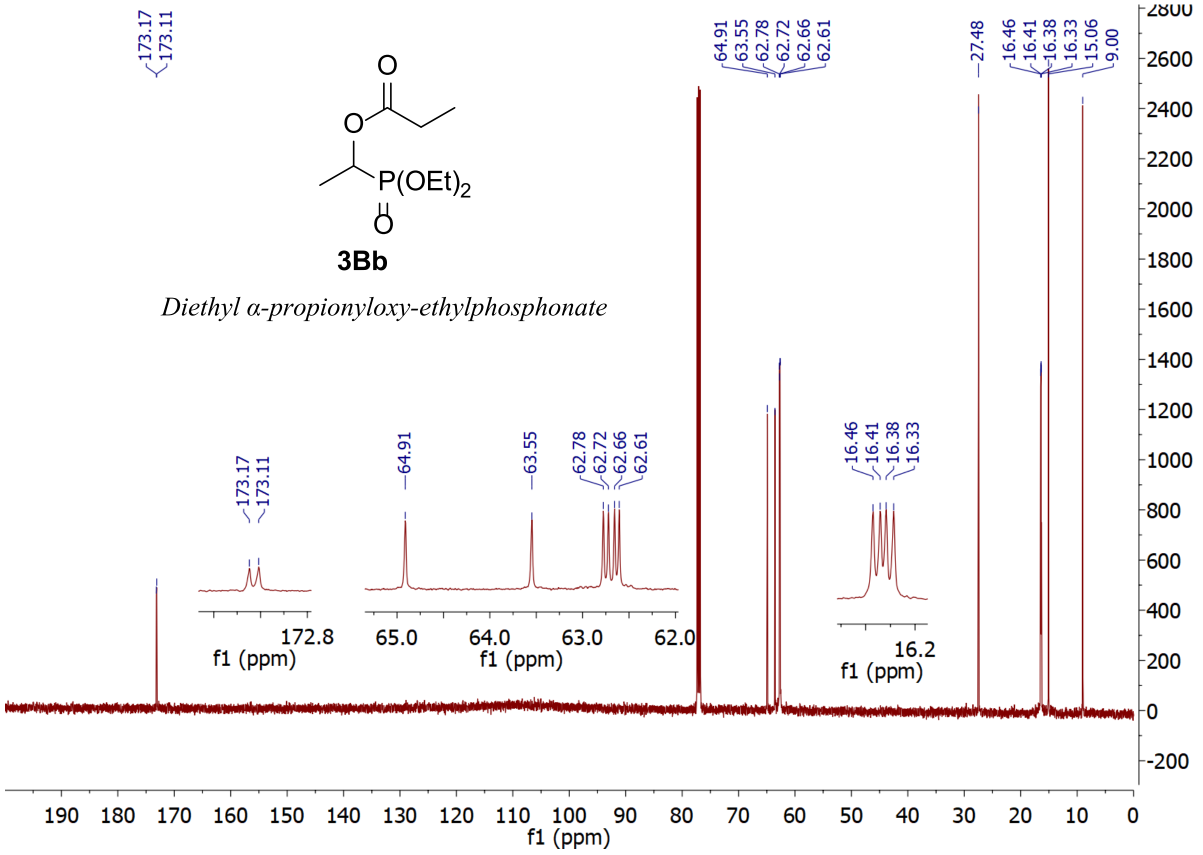

^{1}H NMR (500 MHz, $CDCl_3$) spectra for 3Bb

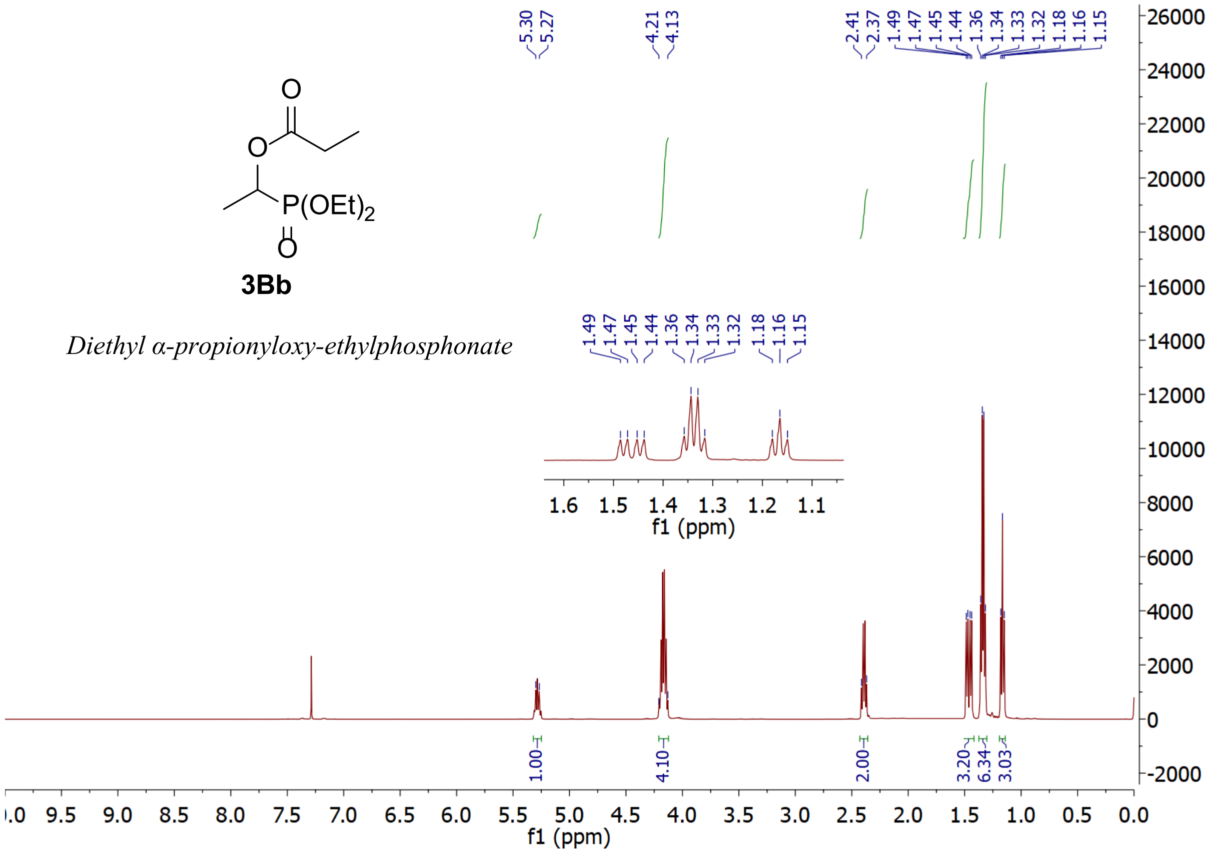

3Bb

Diethyl α-propionyloxy-ethylphosphonate

^{31}P {^{1}H} NMR (202 MHz, $CDCl_3$) spectra for 3Cb

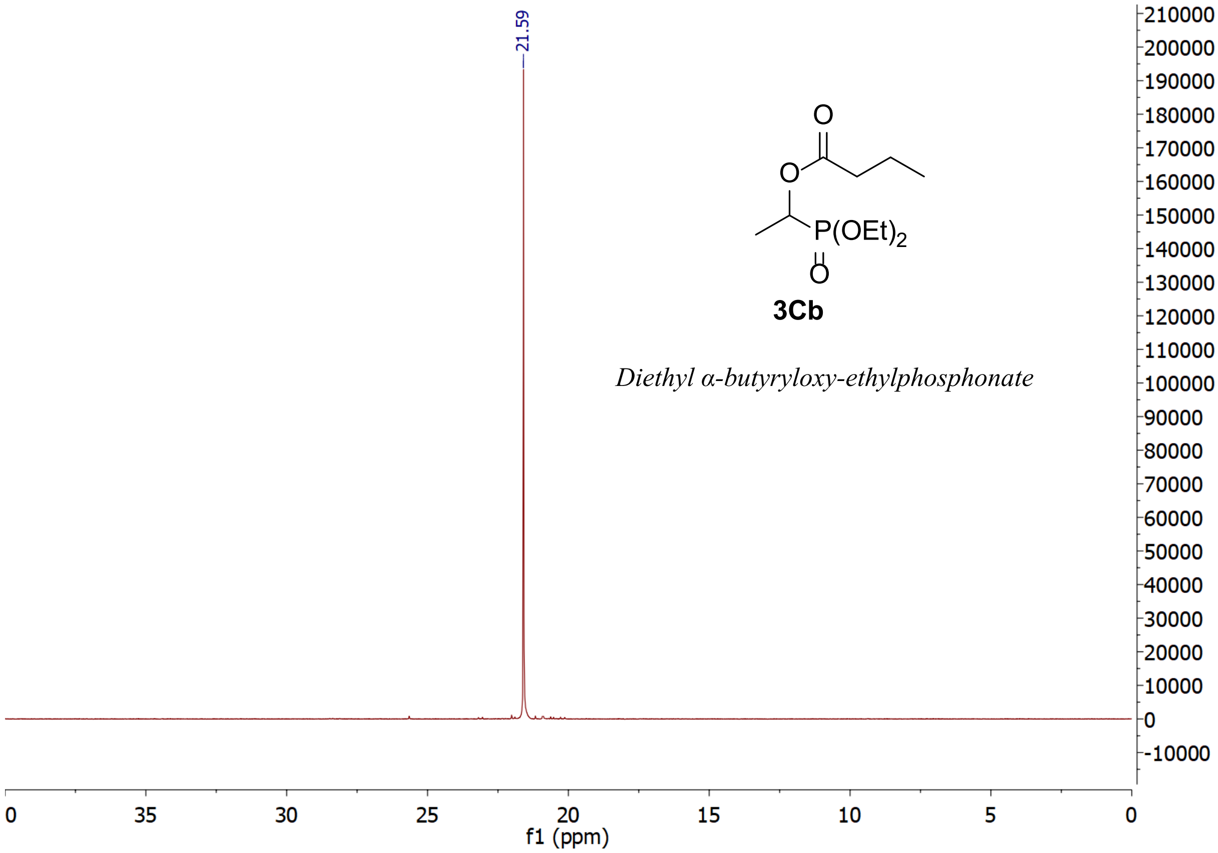

3Cb

Diethyl α-butyryloxy-ethylphosphonate

^{13}C {^{1}H} NMR (126 MHz, $CDCl_3$) spectra for 3Cb

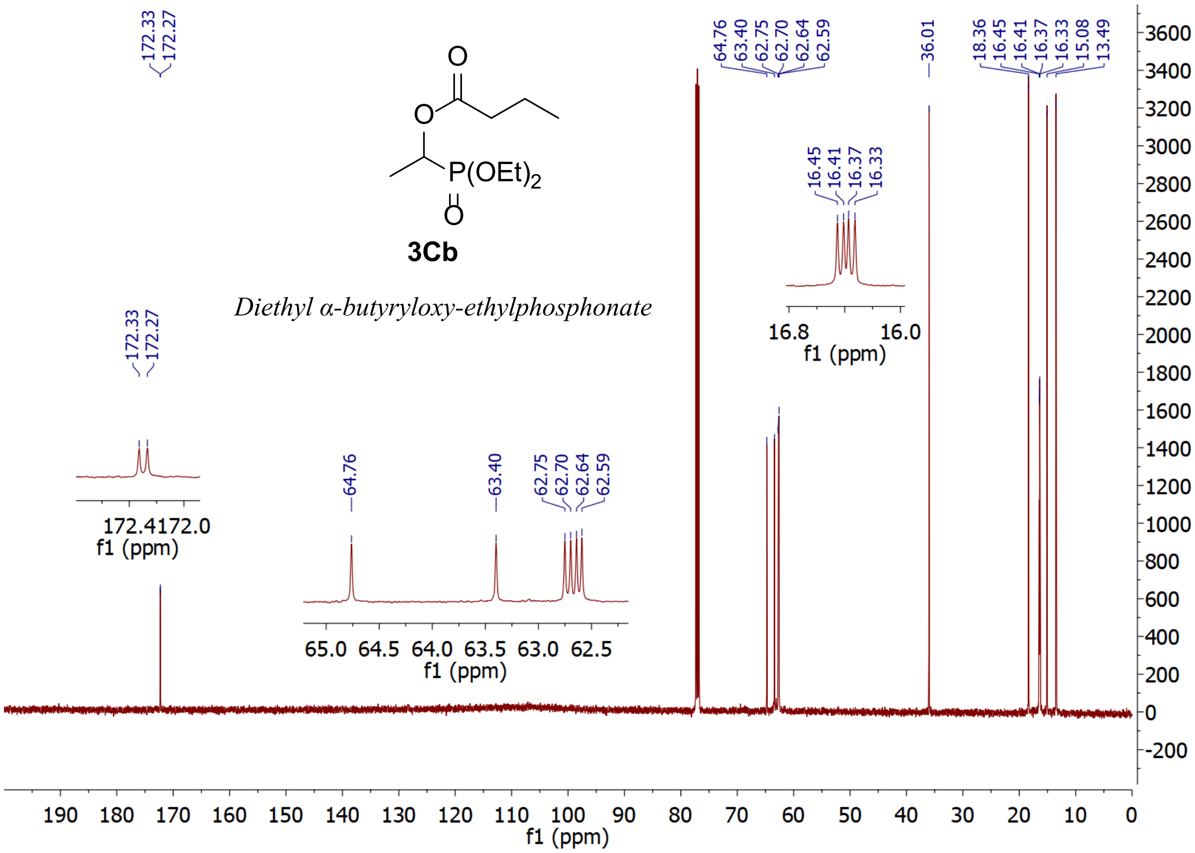

^{1}H NMR (500 MHz, $CDCl_3$) spectra for 3Cb

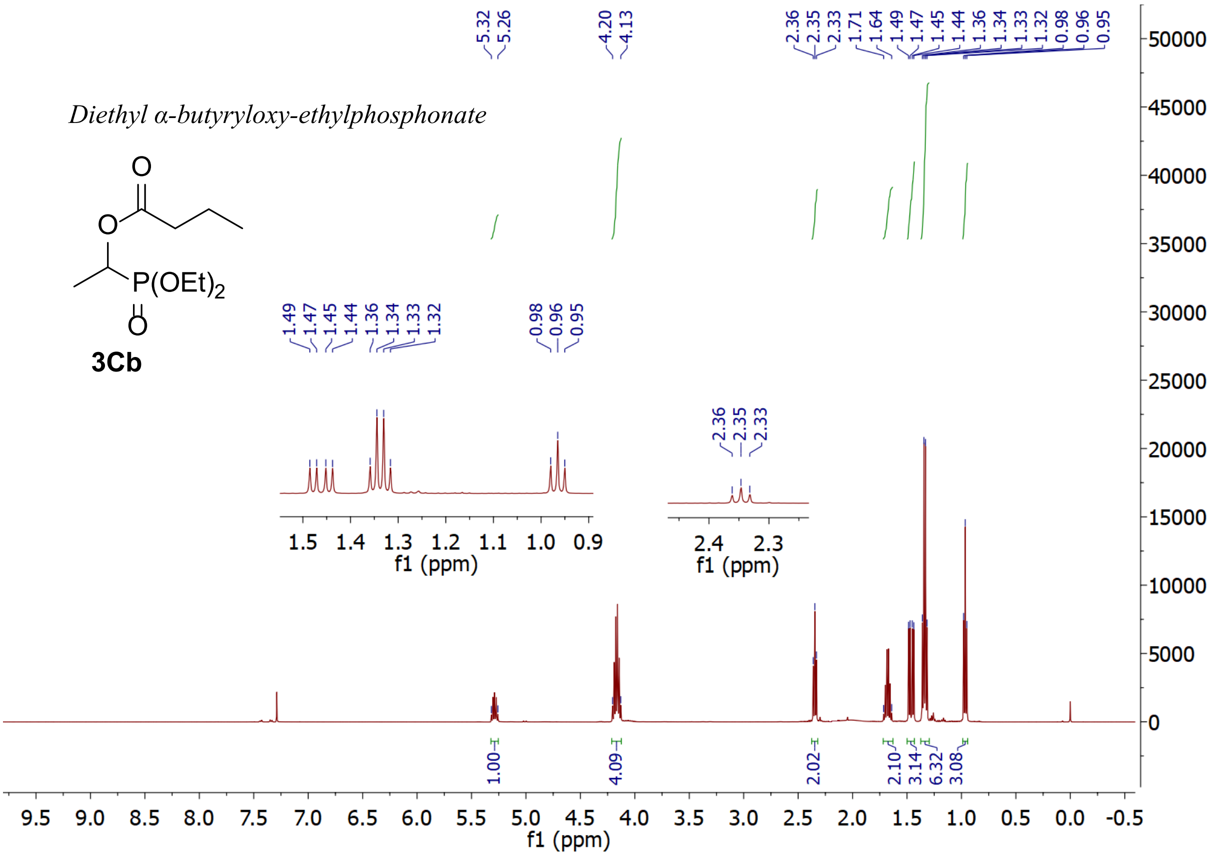

^{31}P {^{1}H} NMR (202 MHz, $CDCl_3$) spectra for 3Db

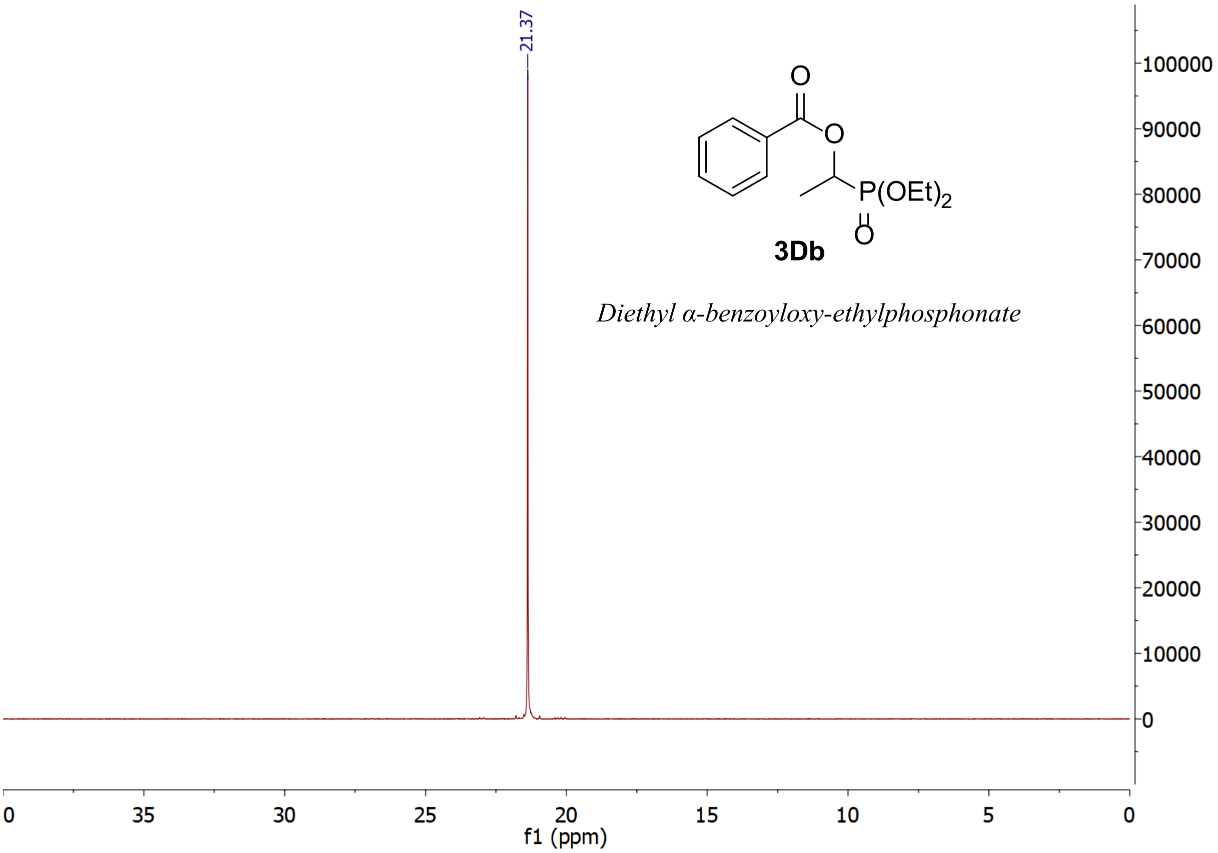

^{31}P {^{1}H} NMR (202 MHz, $CDCl_3$) spectra for 3Ac

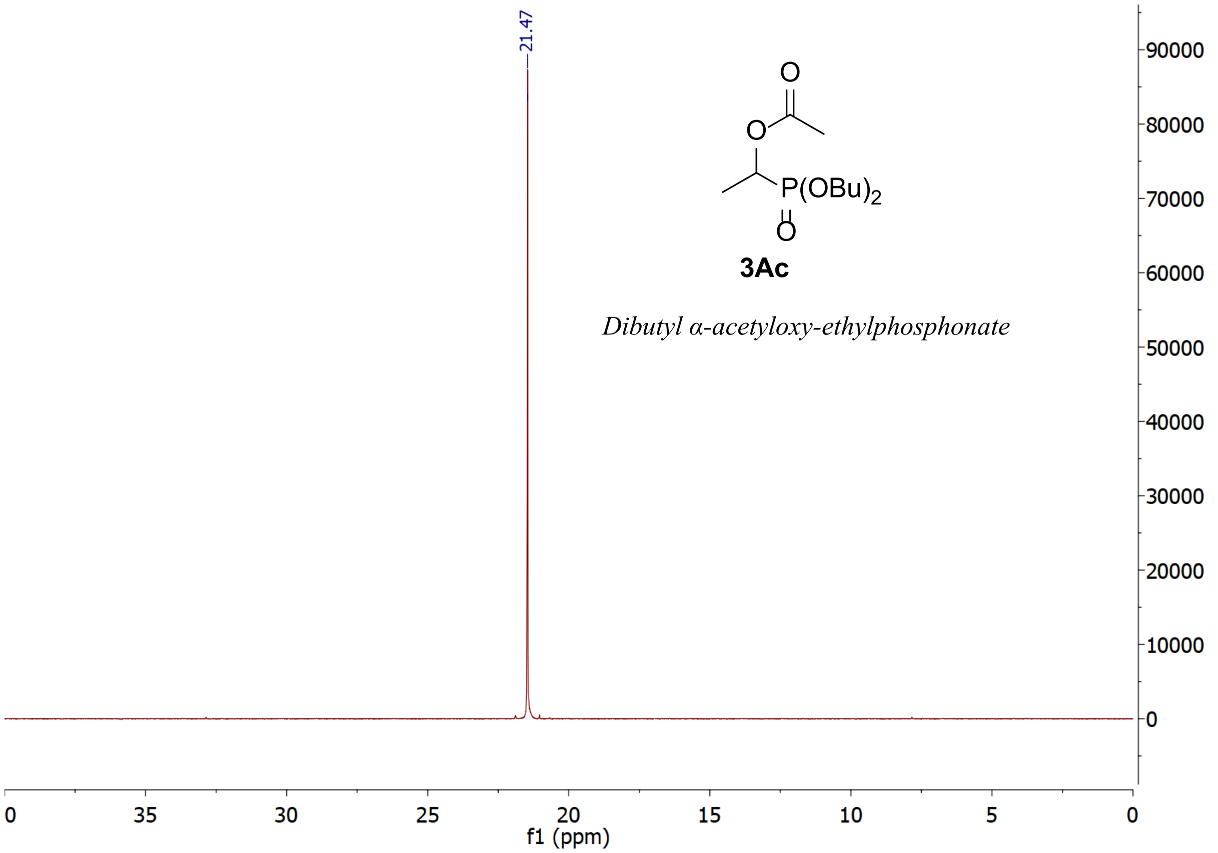

^{13}C {^{1}H} NMR (126 MHz, $CDCl_3$) spectra for 3Ac

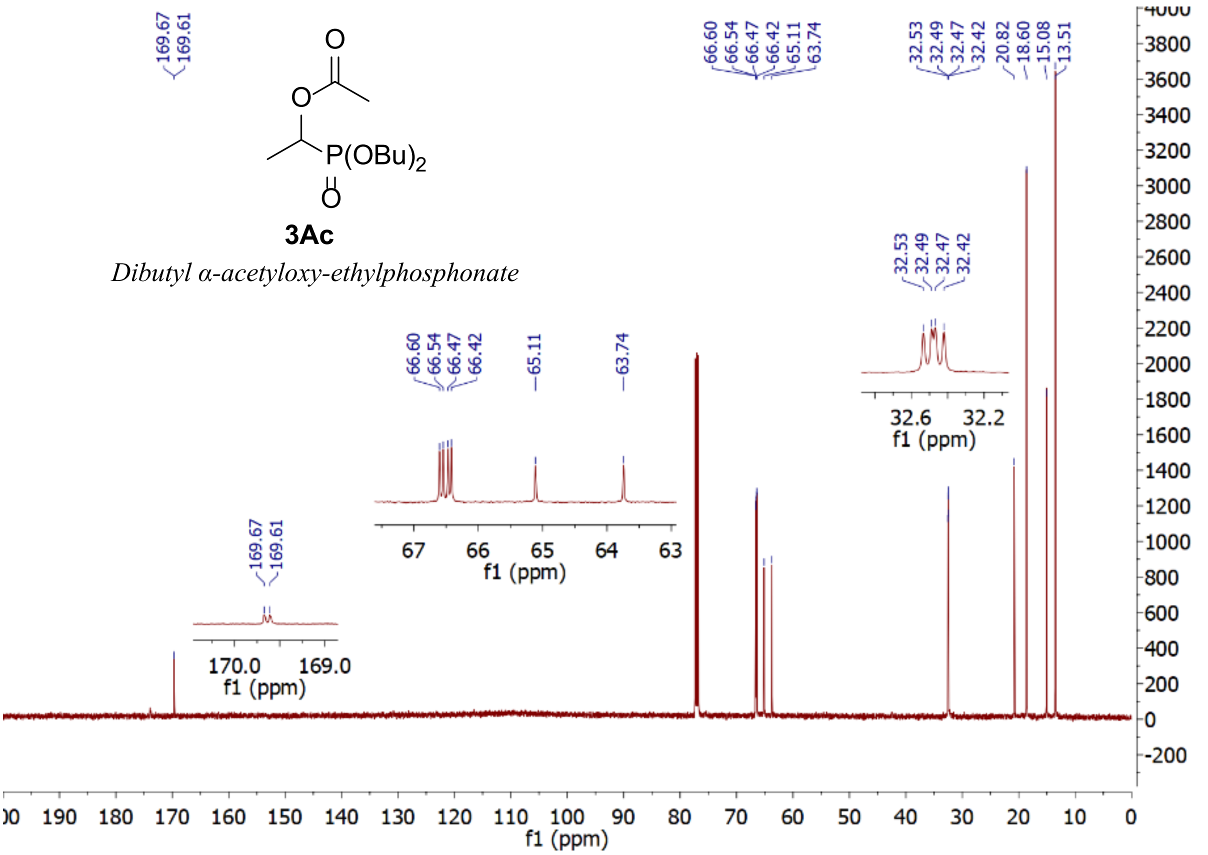

^{1}H NMR (500 MHz, $CDCl_3$) spectra for 3Ac

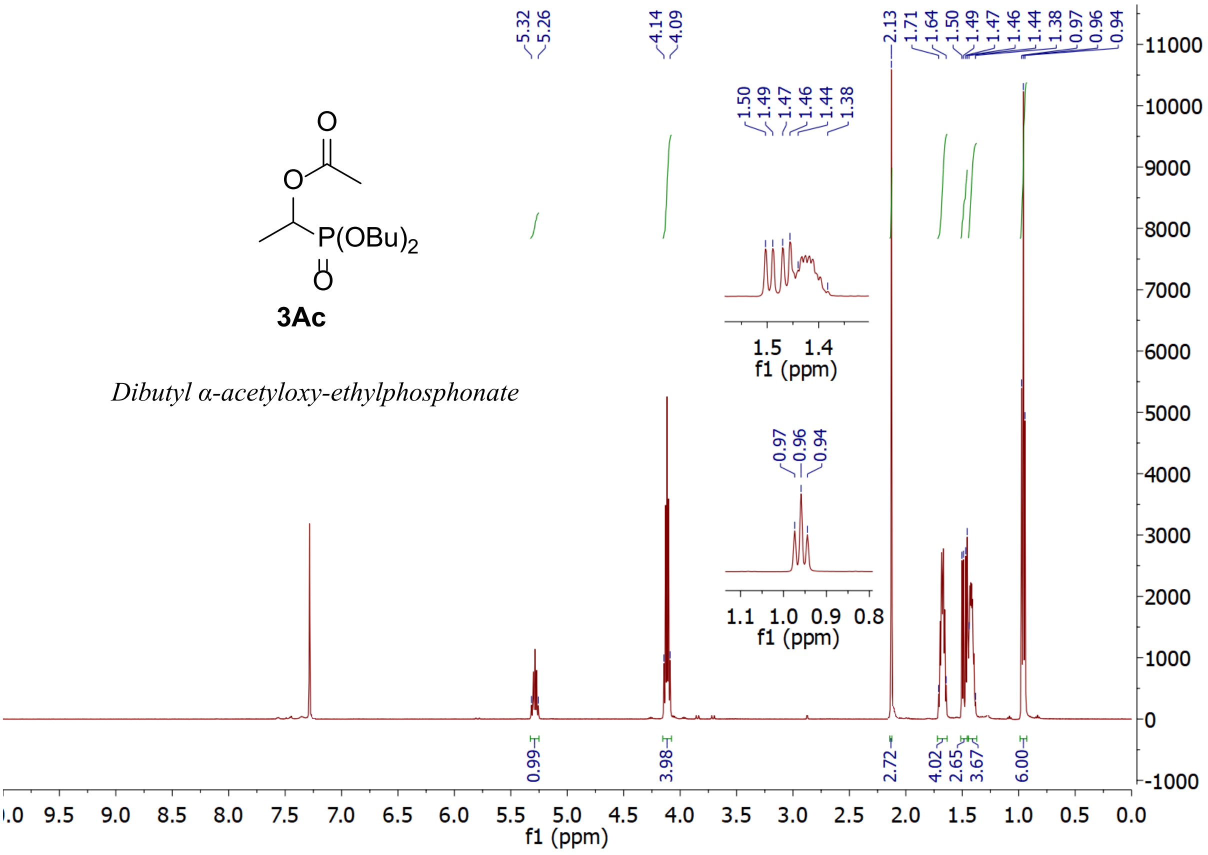

^{31}P {^{1}H} NMR (202 MHz, $CDCl_3$) spectra for 3Ad

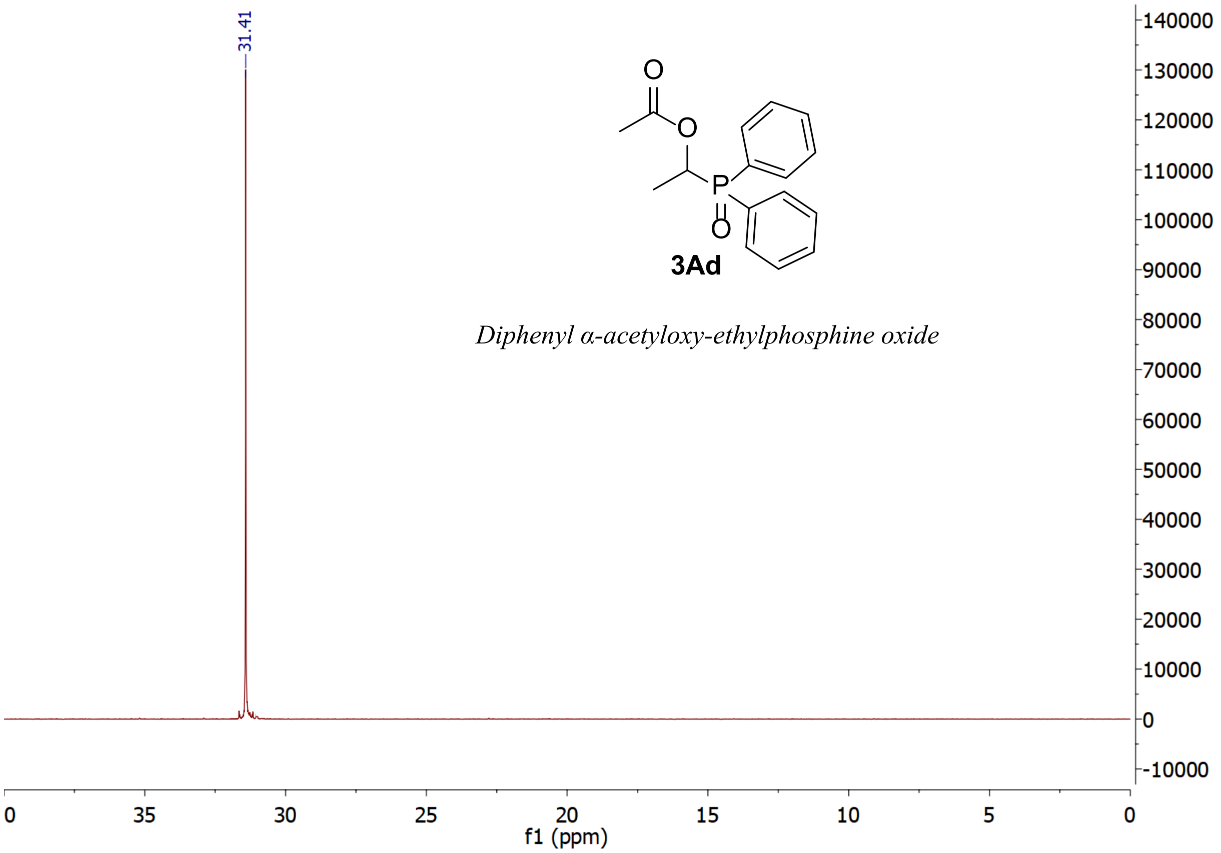

^{13}C {^{1}H} NMR (126 MHz, $CDCl_3$) spectra for 3Ad

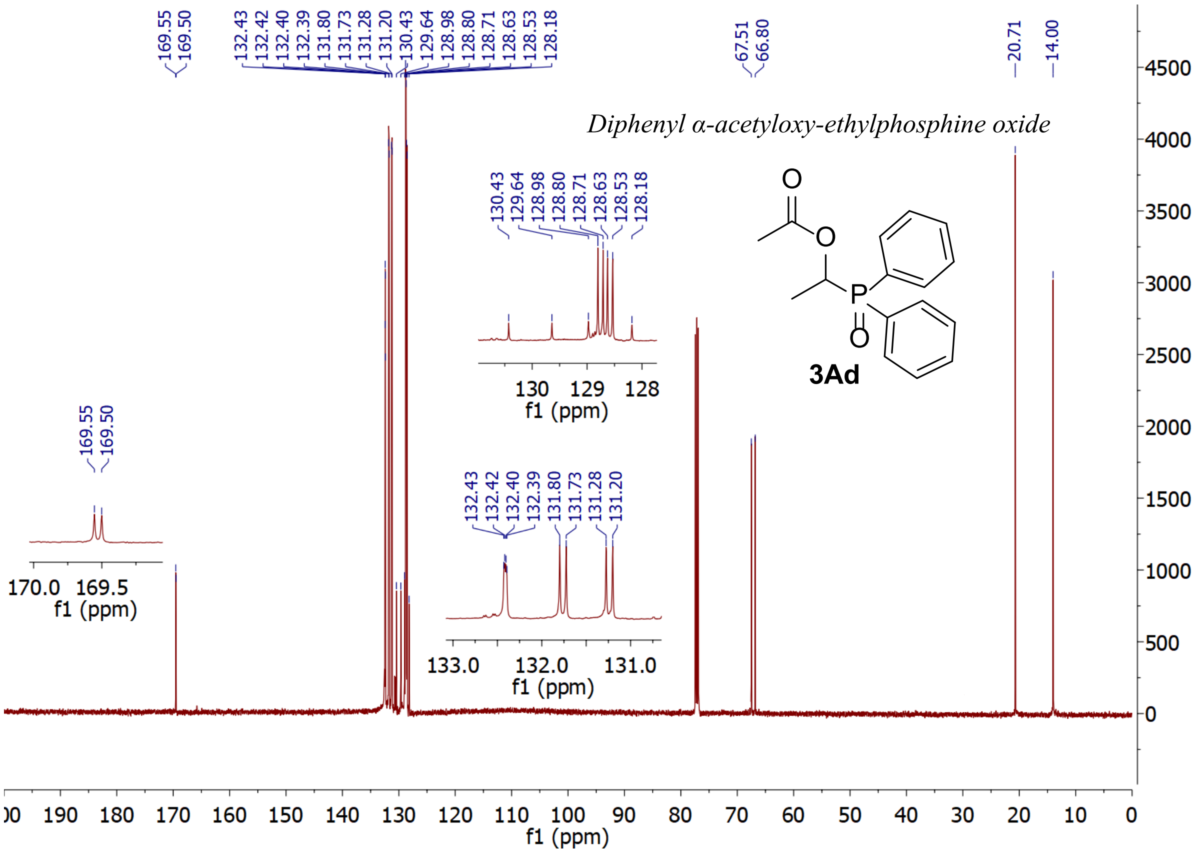

^{1}H NMR (500 MHz, $CDCl_3$) spectra for 3Ad

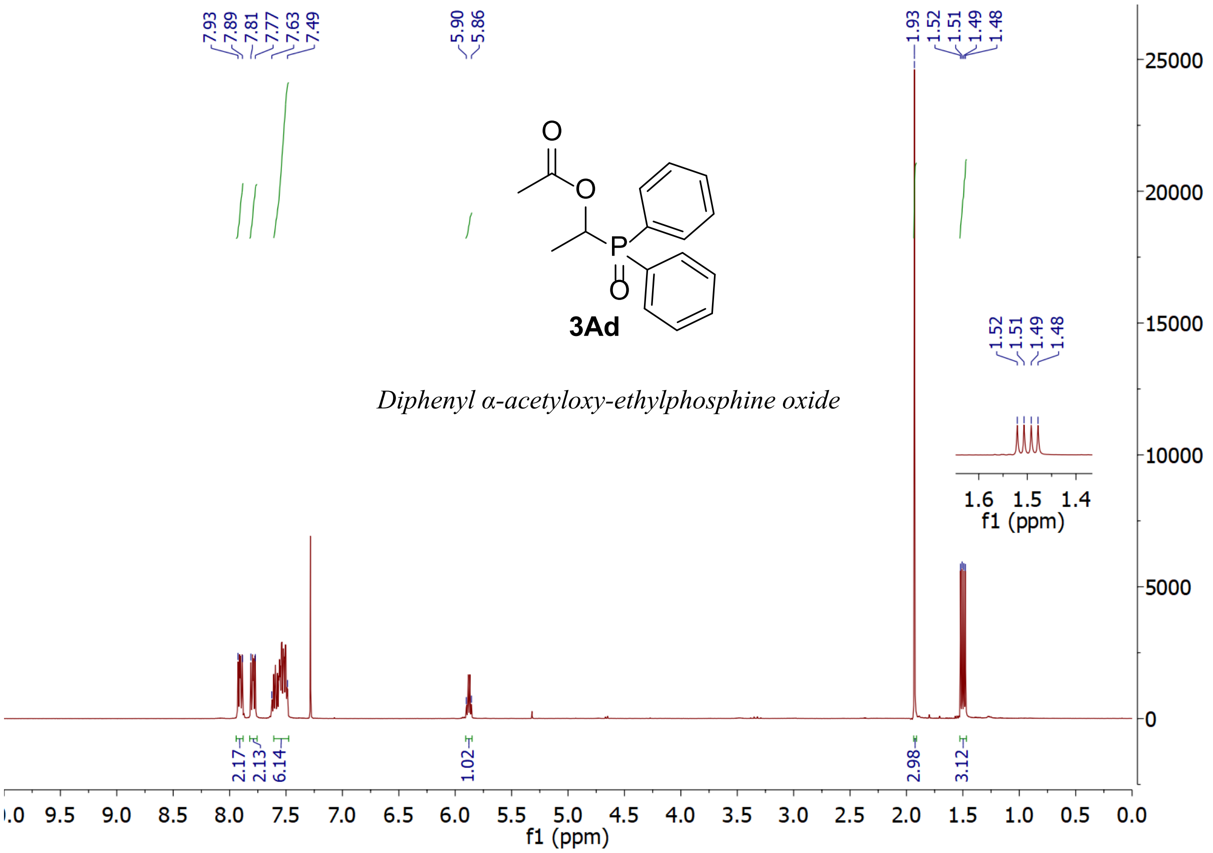

^{31}P {^{1}H} NMR (202 MHz, $CDCl_3$) spectra for 4Ab

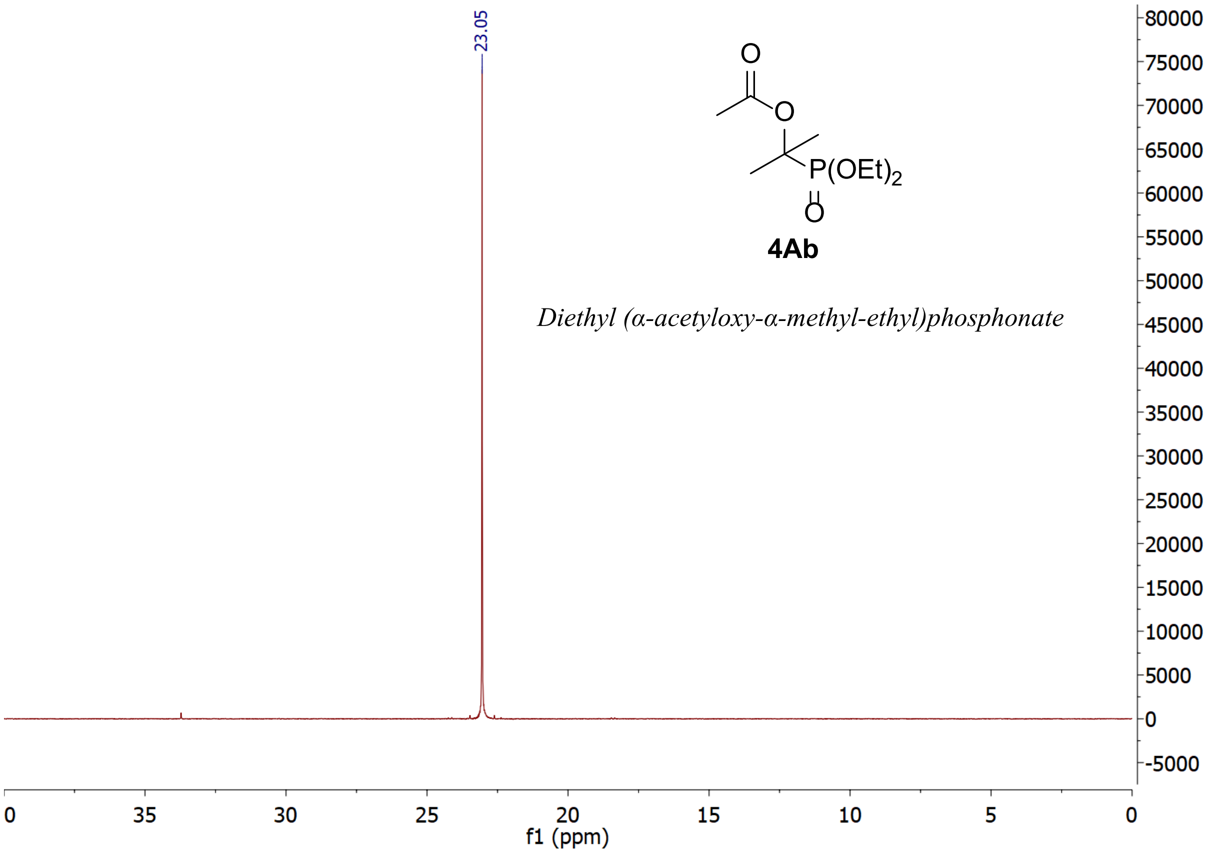

^{13}C {^{1}H} NMR (126 MHz, $CDCl_3$) spectra for 4Ab

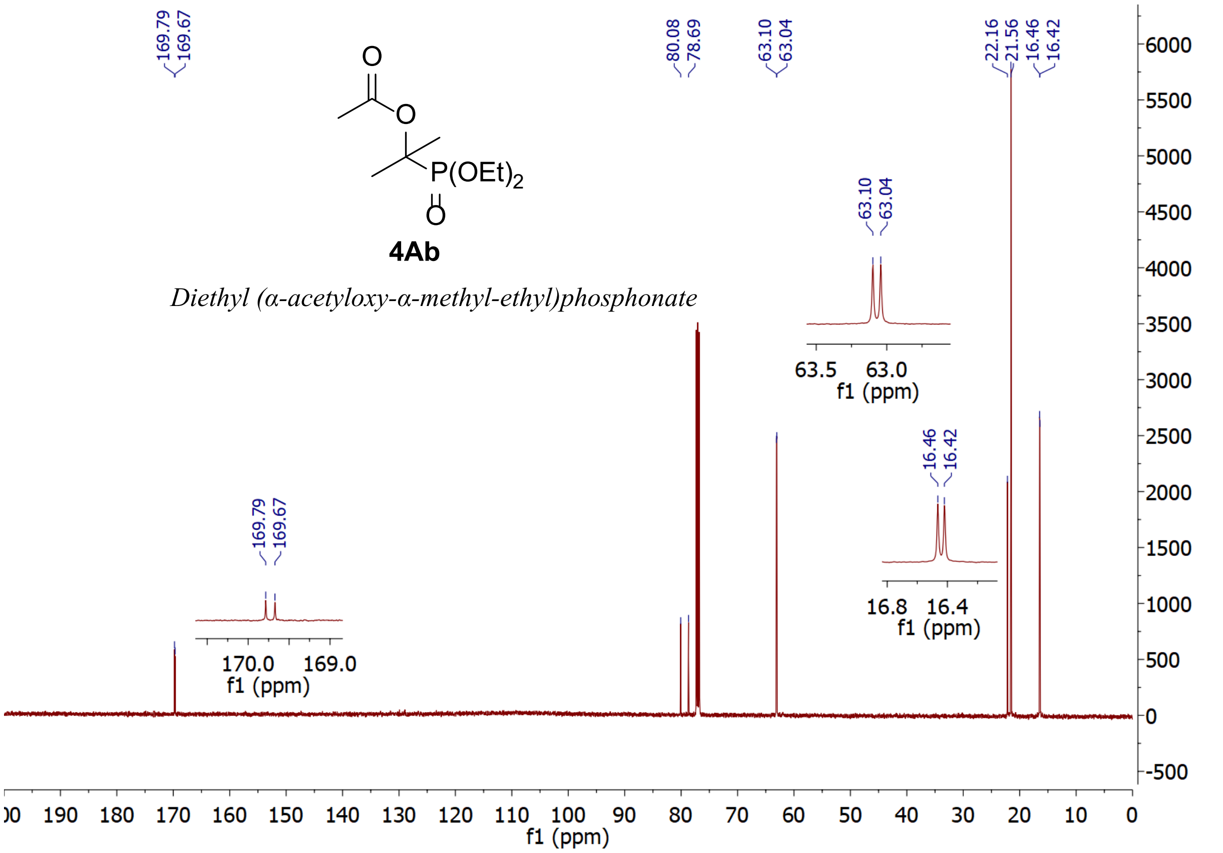

^{1}H NMR (500 MHz, $CDCl_3$) spectra for 4Ab

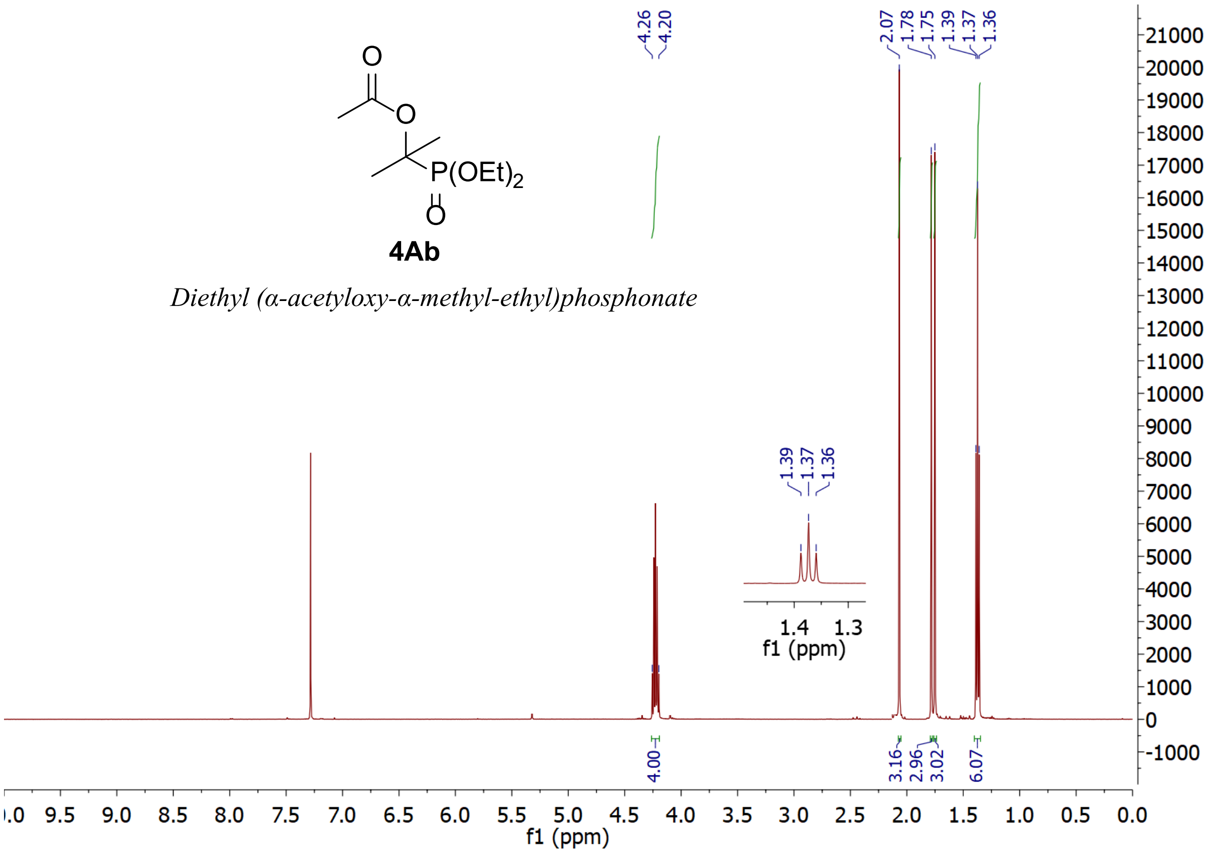

^{31}P {^1H} NMR (202 MHz, $CDCl_3$) spectra for 4Bb

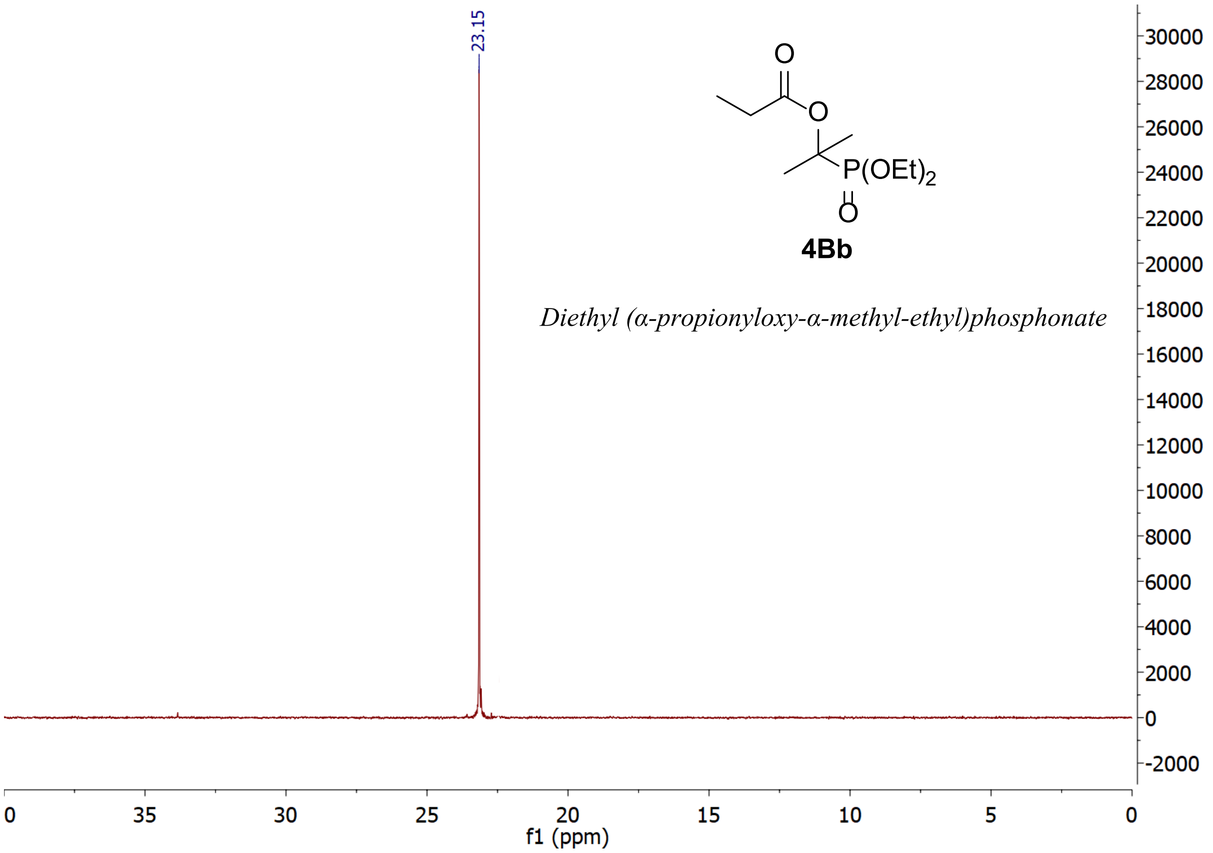

^{13}C {^1H} NMR (126 MHz, $CDCl_3$) spectra for 4Bb

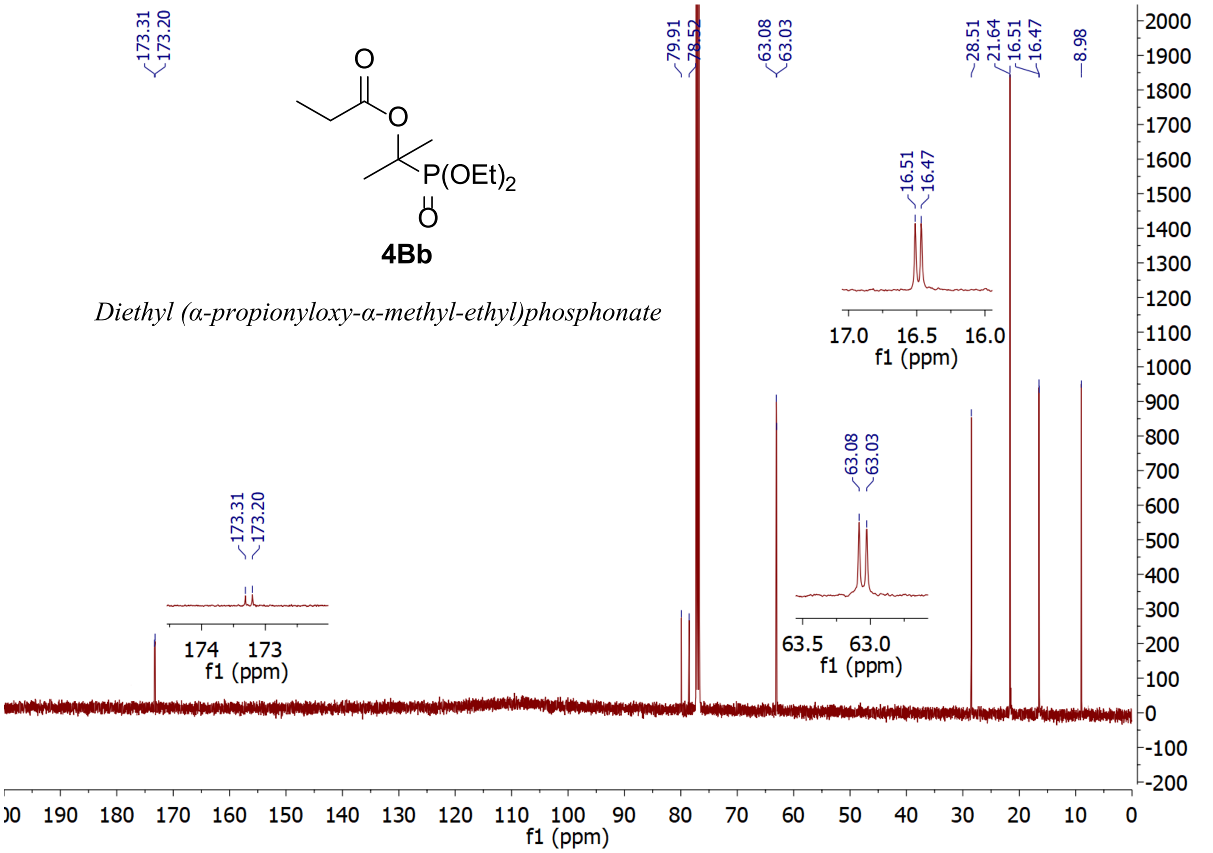

^{1}H NMR (500 MHz, $CDCl_3$) spectra for 4bB

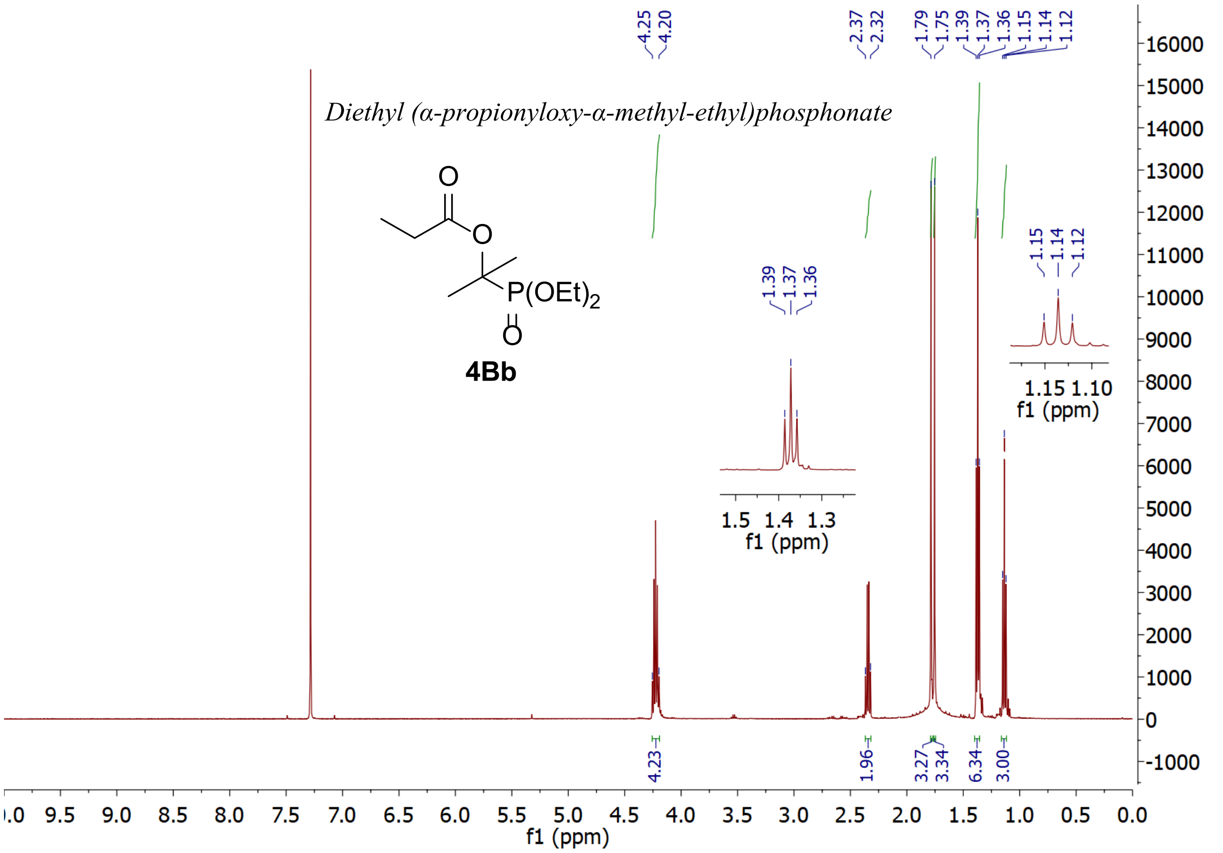

^{31}P {^{1}H} NMR (202 MHz, $CDCl_3$) spectra for 4Cb

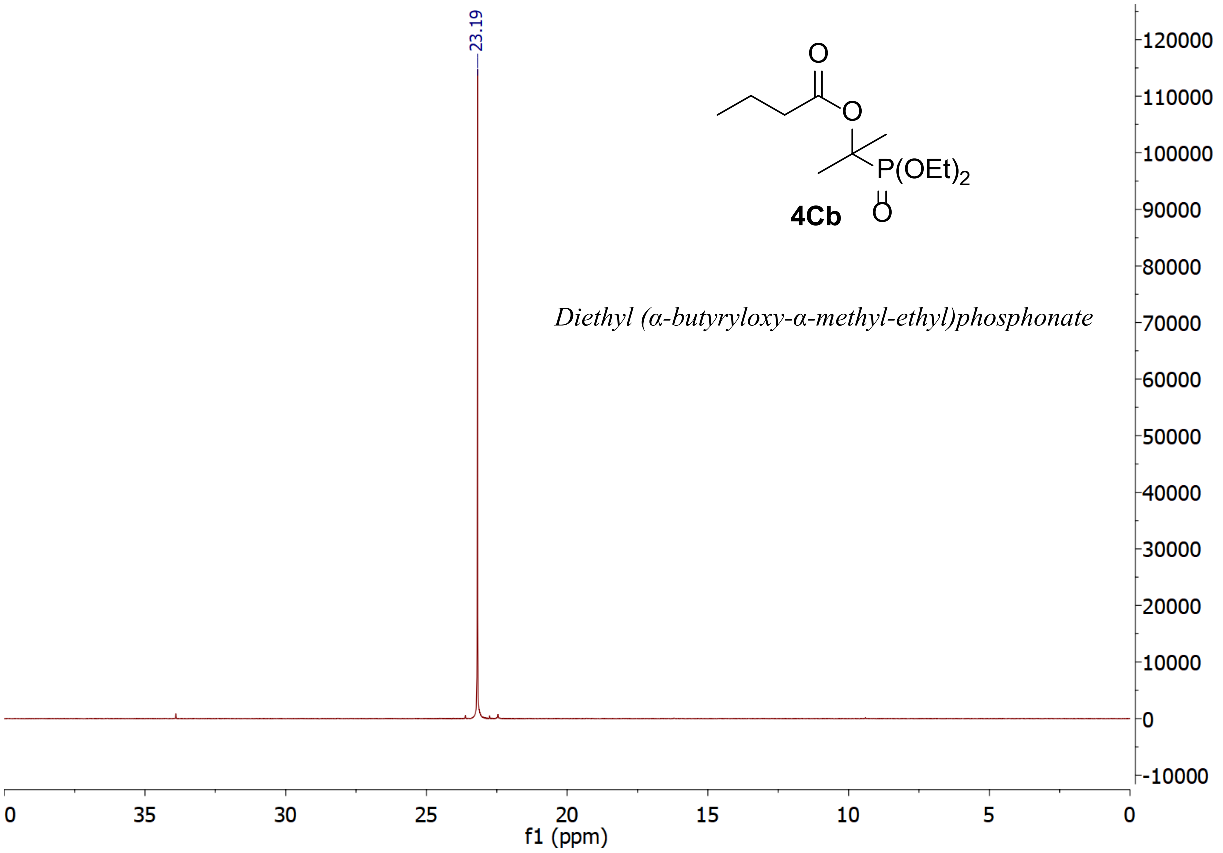

^{13}C {^{1}H} NMR (126 MHz, $CDCl_3$) spectra for 4Cb

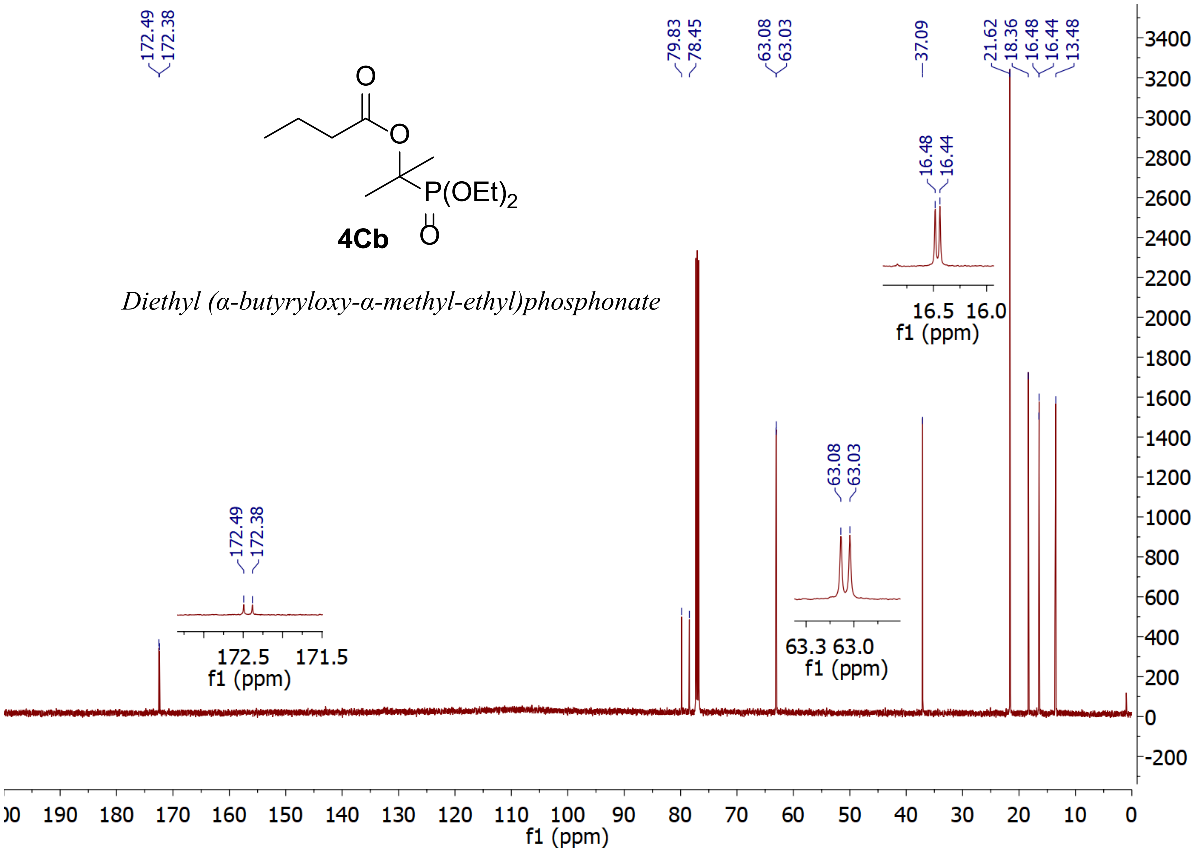

^{1}H NMR (500 MHz, $CDCl_3$) spectra for 4Cb

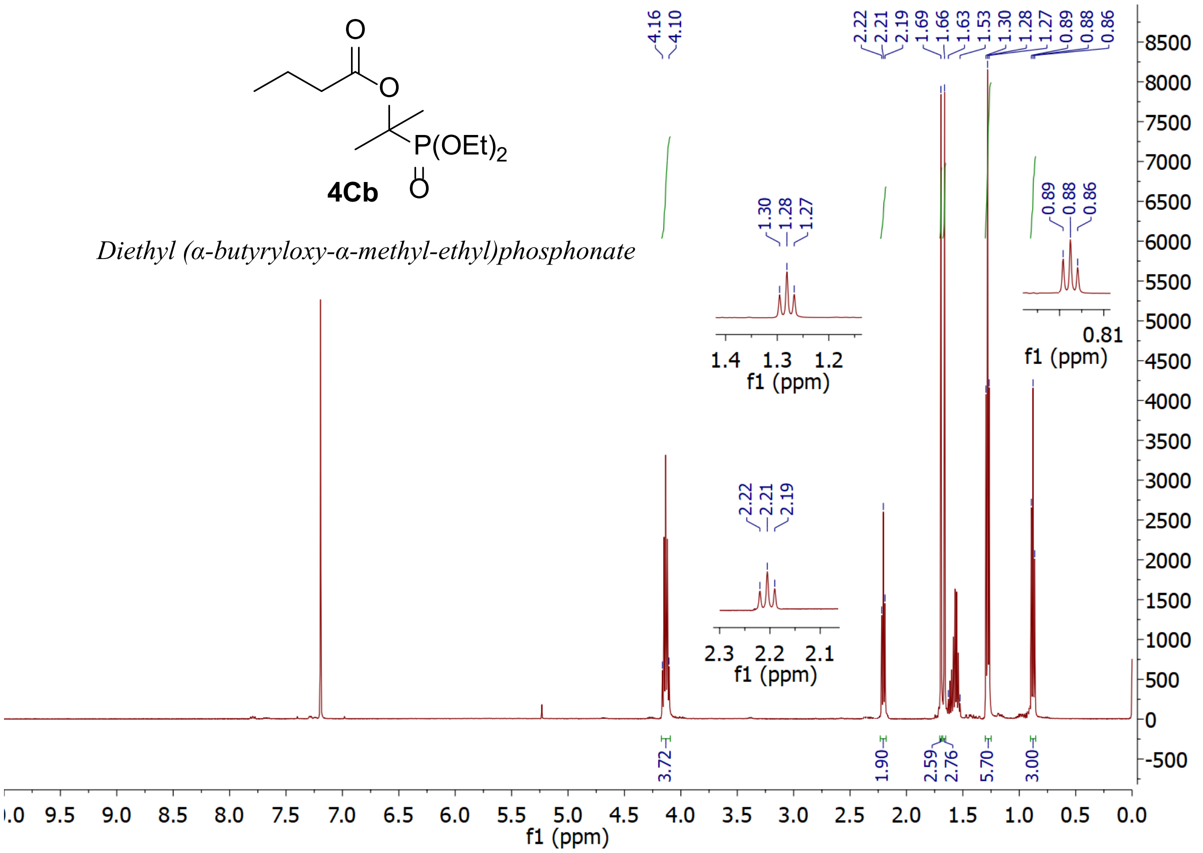

^{31}P {^{1}H} NMR (202 MHz, $CDCl_3$) spectra for 4Ac

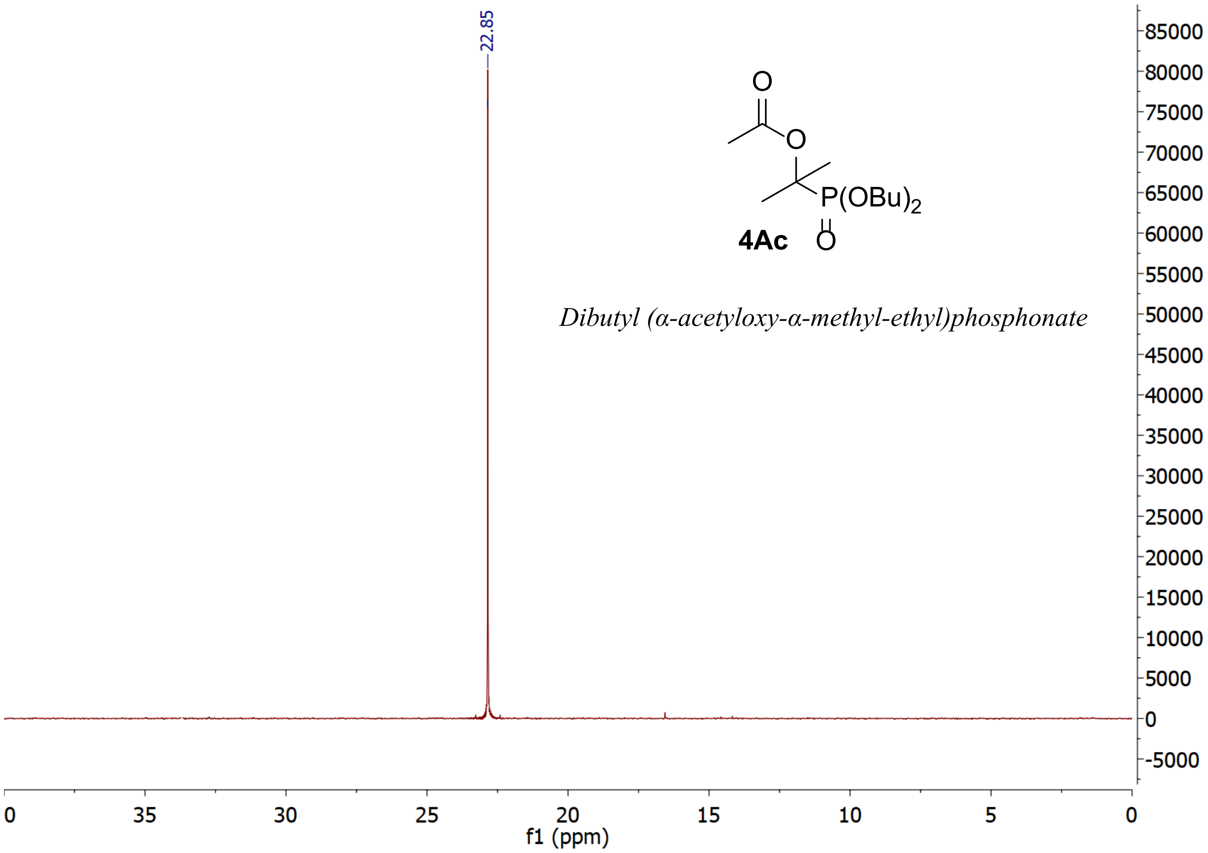

^{13}C {^{1}H} NMR (126 MHz, $CDCl_3$) spectra for 4Ac

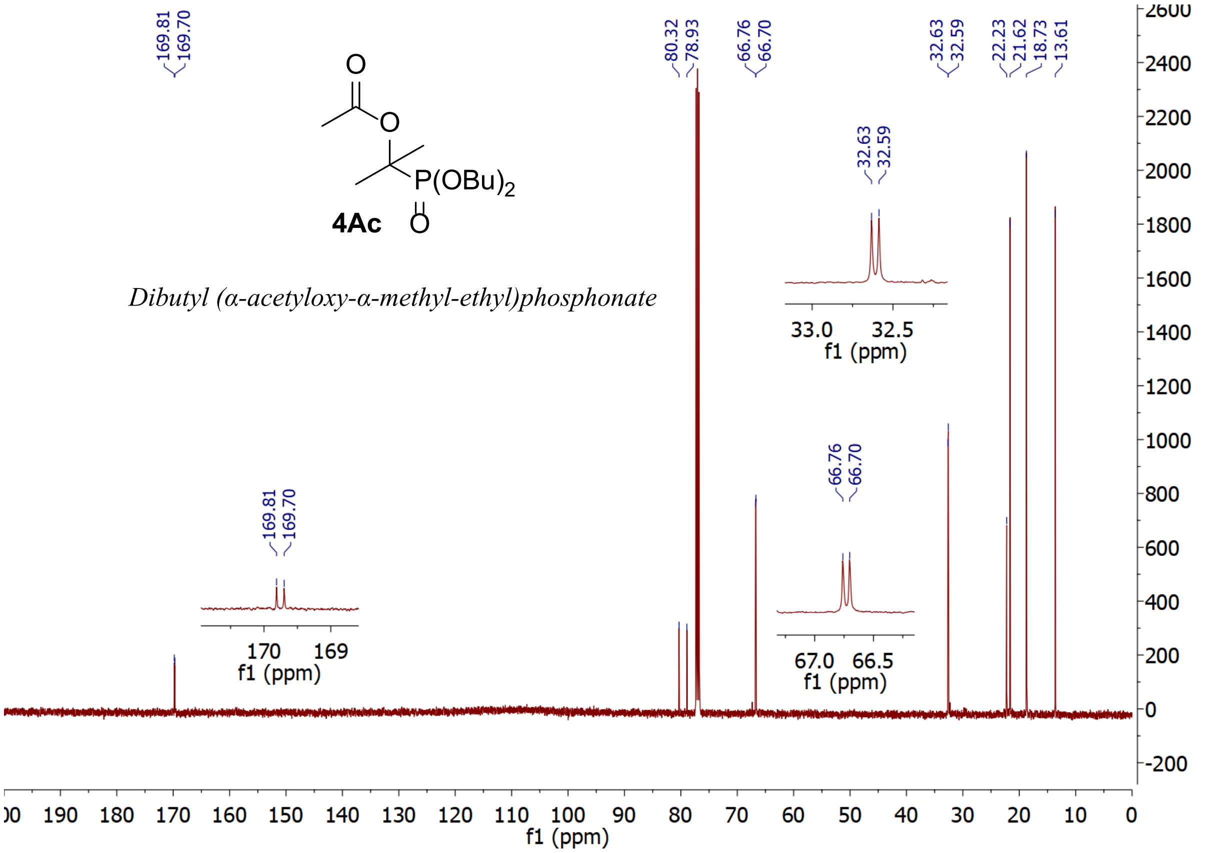

^{1}H NMR (500 MHz, $CDCl_3$) spectra for 4Ac

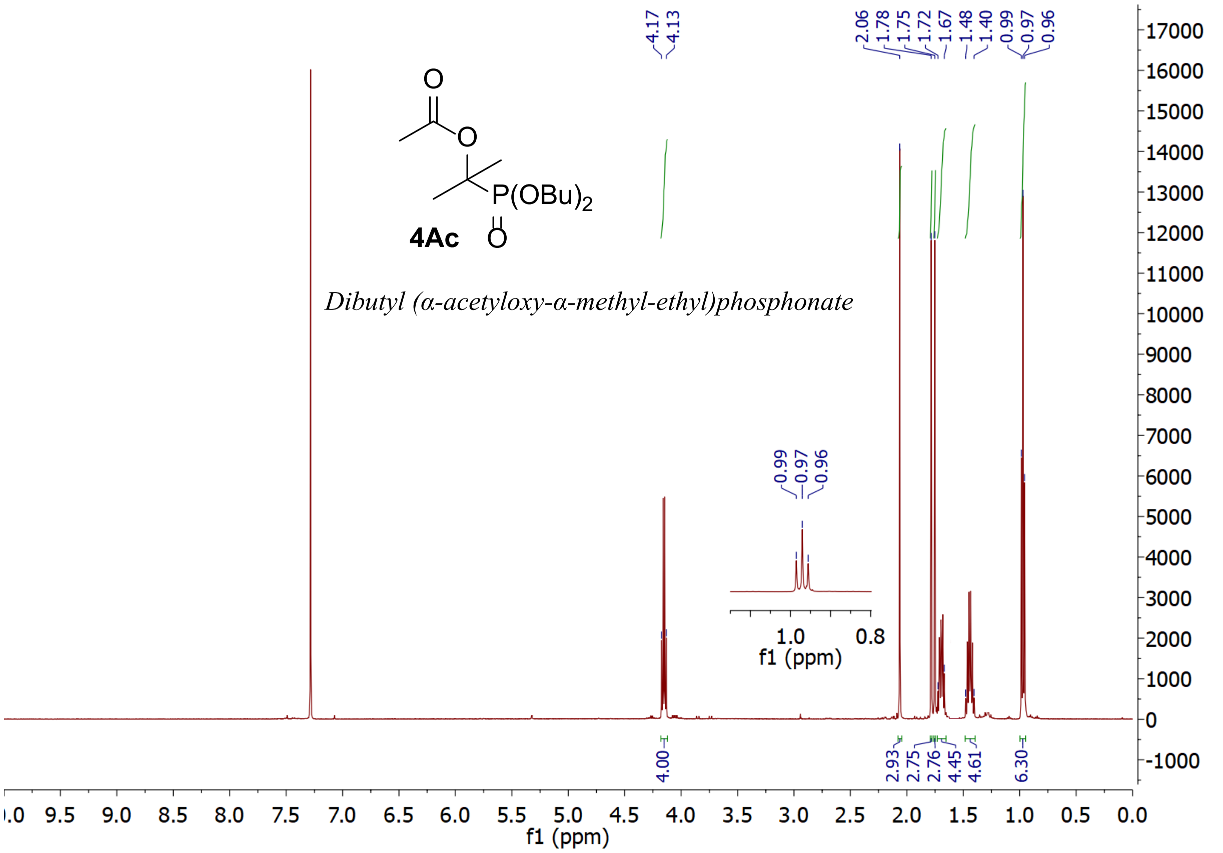

^{31}P {^{1}H} NMR (202 MHz, $CDCl_3$) spectra for 5

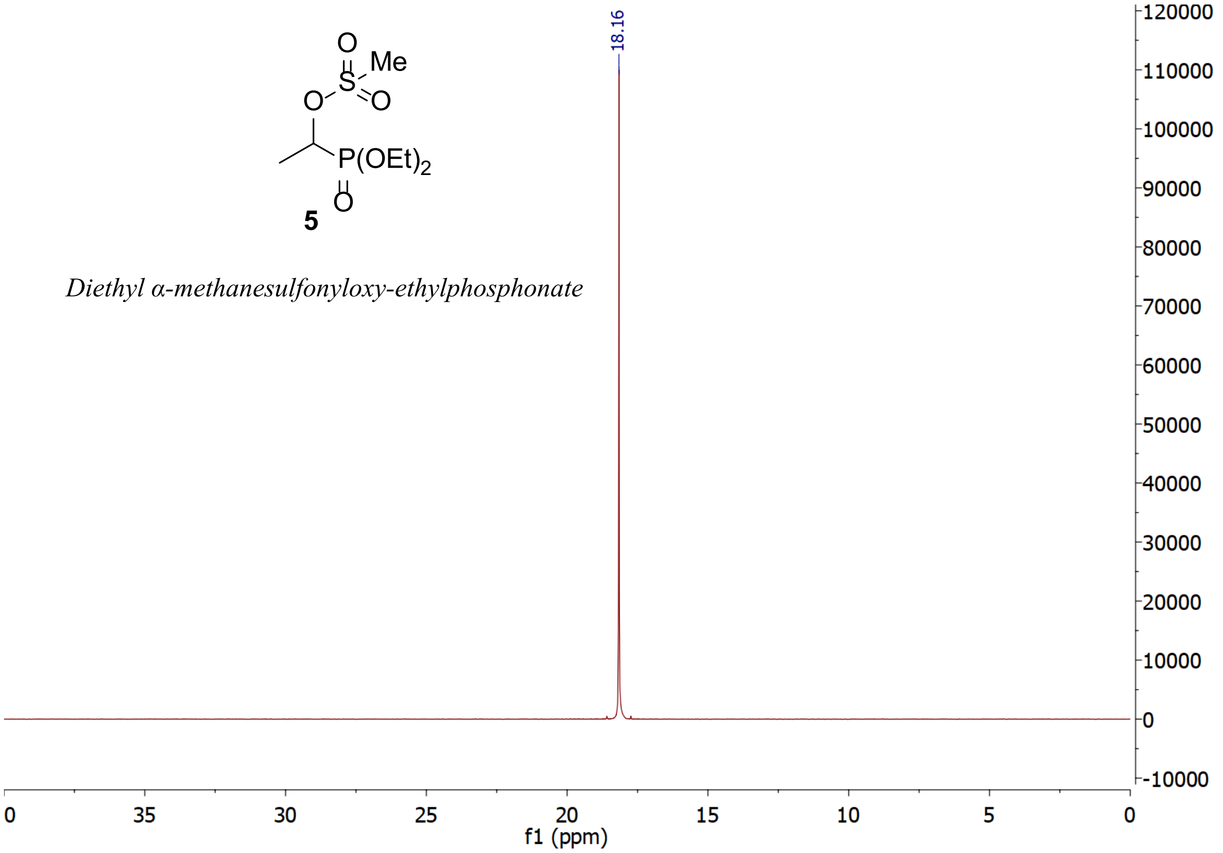

$^{13}C\{^1H\}$ NMR (126 MHz, $CDCl_3$) spectra for 5

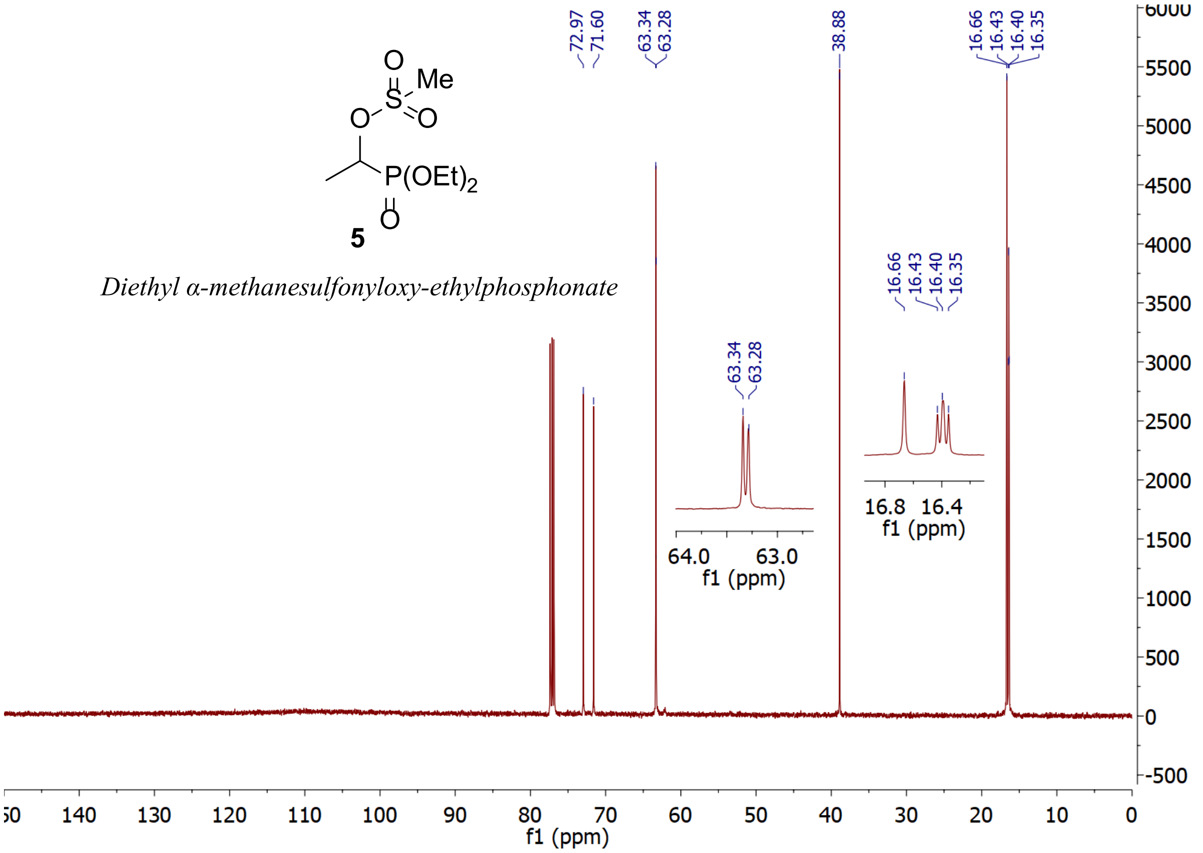

Diethyl α-methanesulfonyloxy-ethylphosphonate

^1H NMR (500 MHz, $CDCl_3$) spectra for 5

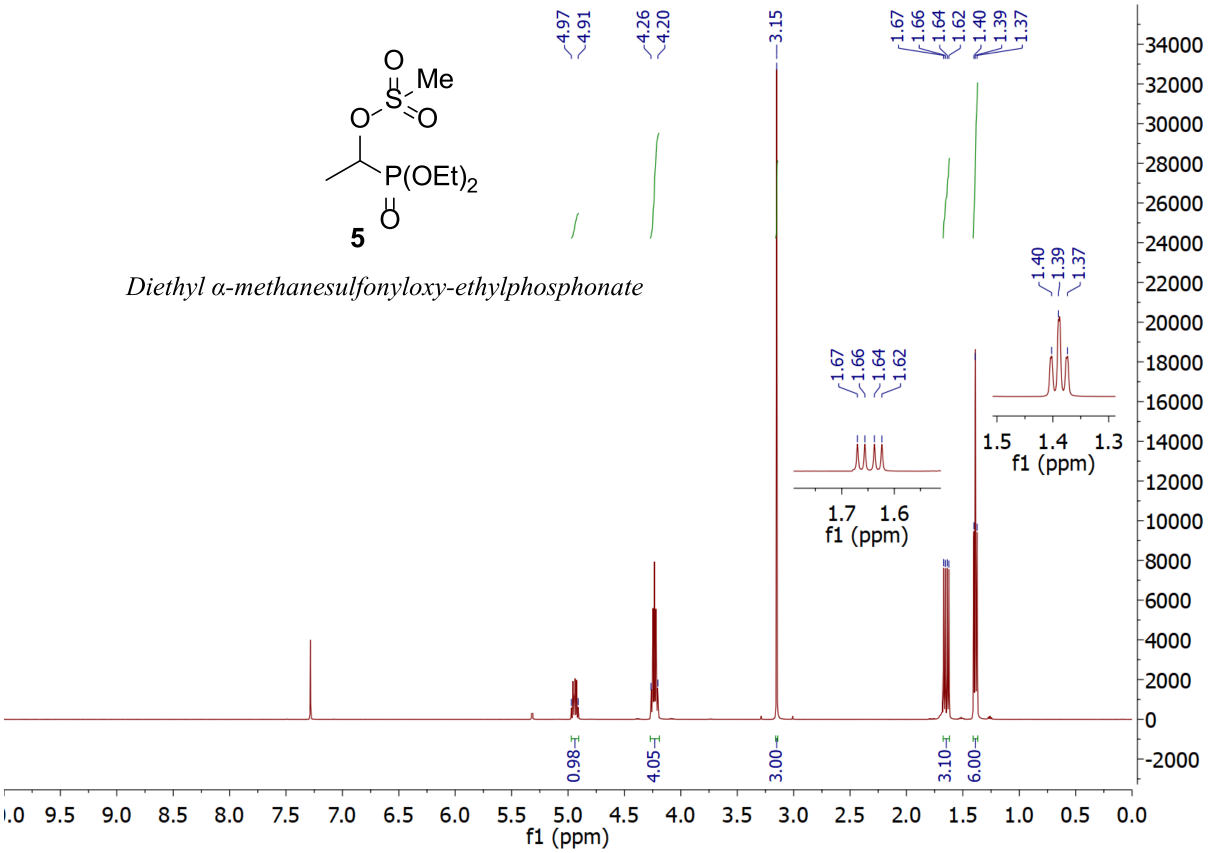

Diethyl α-methanesulfonyloxy-ethylphosphonate

^{31}P {^{1}H} NMR (202 MHz, $CDCl_3$) spectra for 6a

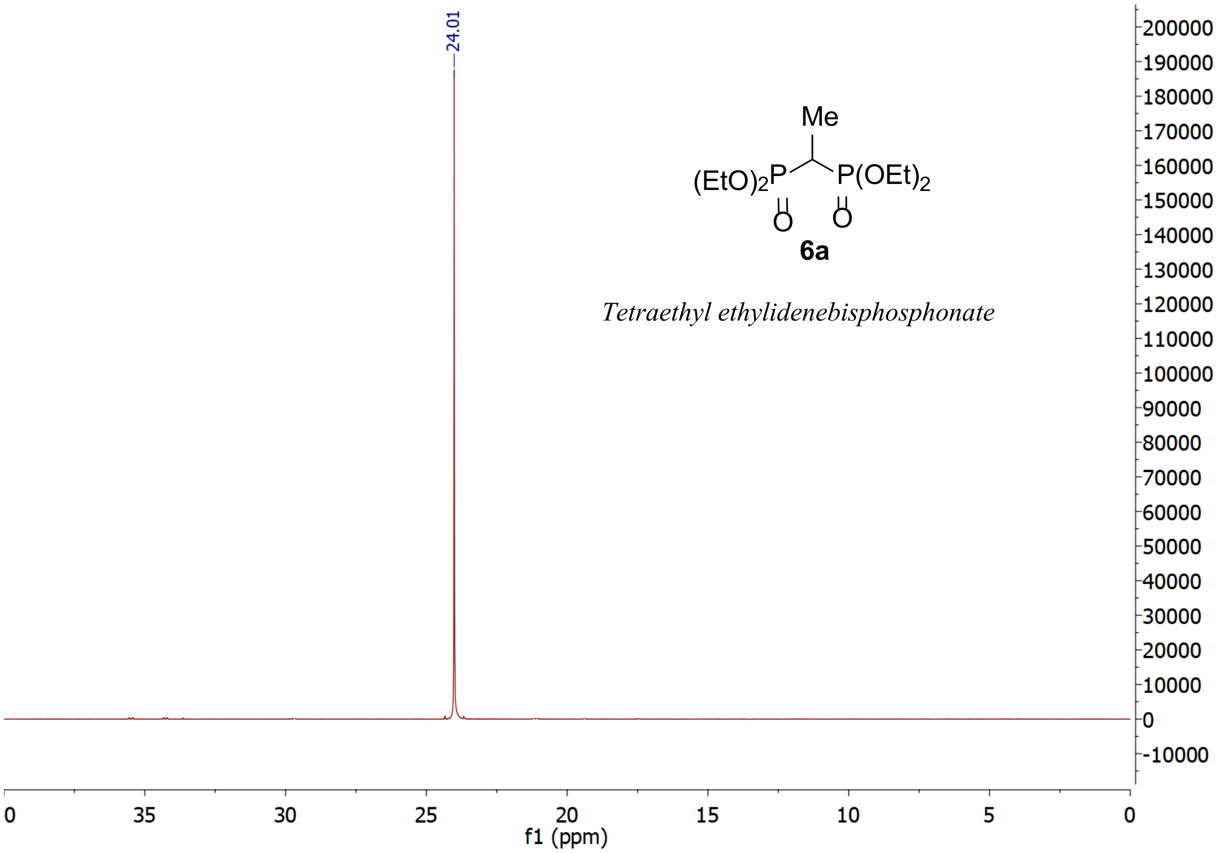

^{31}P {^{1}H} NMR (202 MHz, $CDCl_3$) spectra for 6b

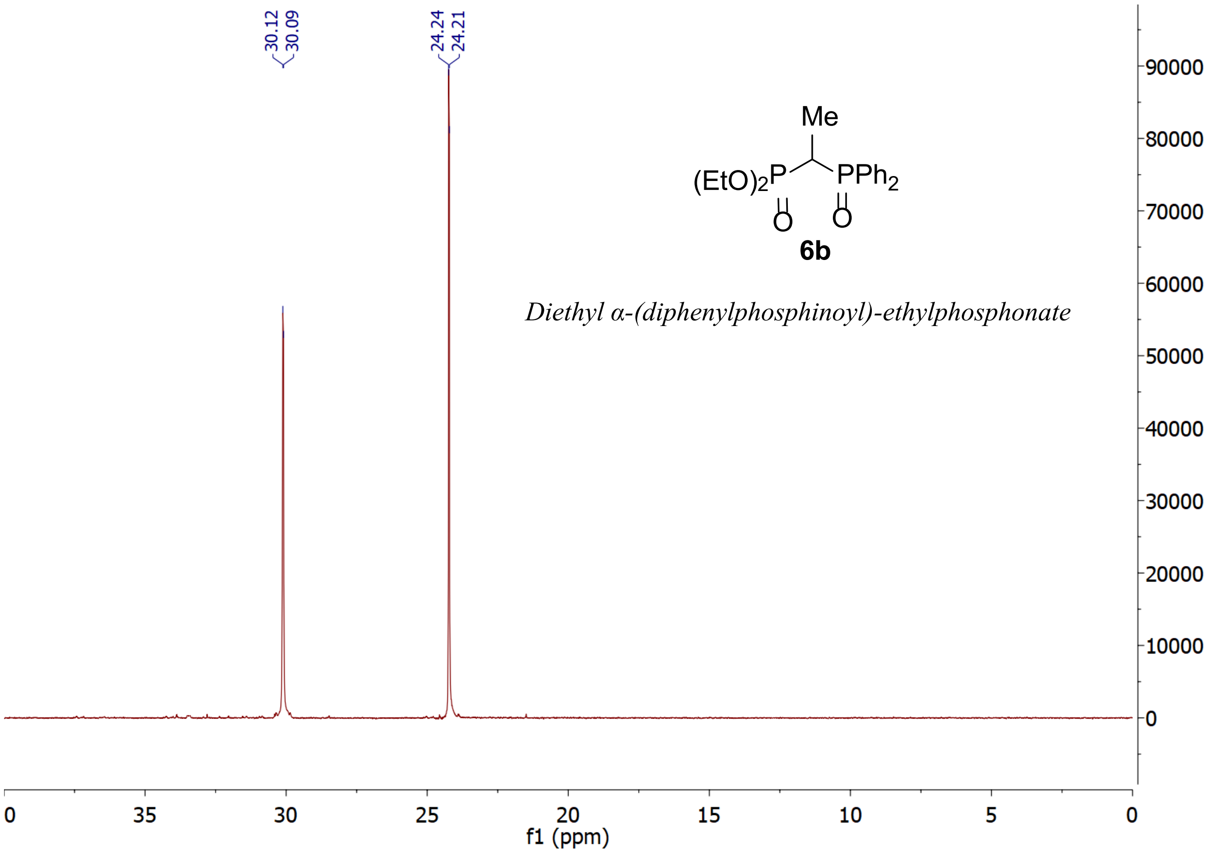

Supplement: Supplementary file 1 [file molecules-30-00428-s001.zip › molecules-3396003-supplementary.pdf]
